# A fluorobenzene-bound dysprosium half-sandwich dication single-molecule magnet†

**DOI:** 10.1039/d4sc06661h

**Published:** 2024-12-04

**Authors:** Sophie C. Corner, William J. A. Blackmore, Gemma K. Gransbury, Andrea Mattioni, George F. S. Whitehead, Nicholas F. Chilton, David P. Mills

**Affiliations:** a Department of Chemistry, The University of Manchester Oxford Road Manchester M13 9PL UK nicholas.chilton@anu.edu.au david.mills@manchester.ac.uk; b Research School of Chemistry, The Australian National University Building 137, Sullivans Creek Road Canberra ACT 2601 Australia

## Abstract

Dysprosium single-molecule magnets (SMMs) with two mutually *trans*-anionic ligands have shown large crystal field (CF) splitting, giving record effective energy barriers to magnetic reversal (*U*_eff_) and hysteresis temperatures (*T*_H_). However, these complexes tend to be bent, imposing a transverse field that reduces the purity of the *m*_*J*_ projections of the CF states and promotes magnetic relaxation. A complex with only one charge-dense anionic ligand could have more pure CF states, and thus high *U*_eff_ and *T*_H_. Here we report an SMM with this topology, a half-sandwich Dy(iii) complex [Dy(Cp*)(FPh)_6_][{Al[OC(CF_3_)_3_]_3_}_2_(μ-F)]_2_ (1-Dy; Cp* = C_5_Me_5_), and its Y(iii) analogue 1-Y; 1-Dy exhibits *U*_eff_ = 545(30) cm^−1^ and *T*_H_ = 14 K at sweep rates of 22 Oe s^−1^. The Cp* ligand imposes a strong axial CF, which is assisted by one axial fluorobenzene; the five equatorially-bound neutral fluorobenzenes present only weak transverse interactions to give a *pseudo*-pentagonal bipyramidal geometry. The salt metathesis reaction of 1-Y with KCp′′′ (Cp′′′ = {C_5_H_2_(SiMe_3_)_3_-1,2,4}) gave the sandwich complex [Y(Cp′′′)(Cp*)(FPh)_2_][{Al[OC(CF_3_)_3_]_3_}_2_(μ-F)] (4-Y), showing that the fluorobenzenes of 1-Y are easily displaced. We envisage that these methodologies could be adapted in future to prepare high-performance axial Dy SMMs with ligands that are more sterically demanding than Cp*.

## Abstract 2

We show that fluorobenzene-bound dicationic lanthanide complexes containing a single anionic ligand can show interesting single-molecule magnet properties, and can be useful starting materials to heteroleptic complexes containing bulky ligands.

## Graphical abstract

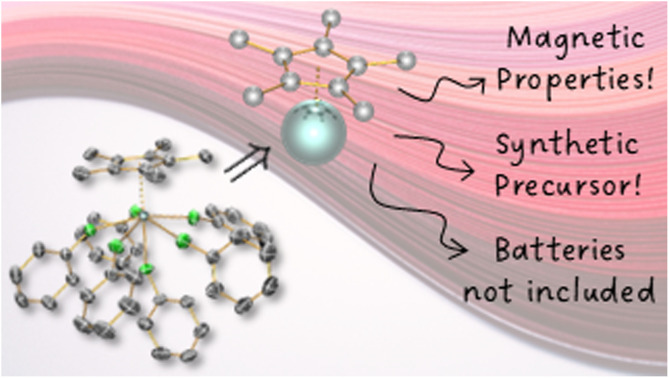

## Introduction

Single-molecule magnets (SMMs) show magnetic remanence,^1^ and lanthanide (Ln) SMMs have provided the largest effective energy barriers to magnetic reversal (*U*_eff_) and hysteresis temperatures (*T*_H_) to date.^2–4^ These advances have been driven by classical electrostatic design criteria to increase magnetic anisotropy, where oblate Dy(iii) and Tb(iii) ions with two mutually *trans*-anionic ligands and maximised crystal fields (CFs) show the largest *U*_eff_ from stabilising the largest ±*m*_*J*_ projections in the ground state and destabilising the smaller ±*m*_*J*_ states.^5–7^ Perfectly linear (*i.e.* with a C_∞_ axis) Dy(iii) and Tb(iii) complexes would have pure ±*m*_*J*_ states, which would suppress two-phonon Raman and quantum tunnelling of magnetization (QTM) relaxation processes, giving high *T*_H_ values. However, any deviation from linearity introduces transverse fields that mix the *m*_*J*_ states, reducing *T*_H_. Given the difficulty of isolating linear Ln(iii) complexes, we considered that a Dy(iii) complex with only a single anionic ligand may show purer ±*m*_*J*_ states than a bent bis-anionic complex and hence show relatively high *T*_H_. Indeed, the theoretical [DyO]^+^ cation has been predicted to have *U*_eff_ > 2084 cm^−1^,^6^ but the isolation of such a coordinatively unsaturated complex is inherently challenging due to a combination of large Ln cations and predominantly ionic bonding regimes favouring high coordination numbers.^8^ The closest molecular analogues of [DyO]^+^ are endohedral fullerenes containing {Dy_2_O} fragments,^9–12^ and these compounds show favourable SMM properties, but magnetic relaxation is promoted by interactions of Dy(iii) ions with the anionic cages.

The first Dy(iii) complex containing no equatorially-bound ligands, the sandwich complex [Dy(Cp^ttt^)_2_][B(C_6_F_5_)_4_] (Cp^ttt^ = C_5_H_2_^*t*^Bu_3_-1,2,4), was reported in 2017; this SMM showed *U*_eff_ = 1223(15) cm^−1^ and *T*_H_ = 60 K due to a combination of the axial ligand field and rigid aromatic Cp^R^ (substituted cyclopentadienyl) rings hindering both Orbach and Raman relaxation.^13^ Related Ln SMMs with higher *U*_eff_ and *T*_H_ values have since been achieved by decreasing Ln–L (L = ligand) distances and increasing L–Ln–L angles,^14–23^ and in an extension of this methodology the dinuclear complex [{Dy(C_5_^i^Pr_5_)}_2_(μ-I)_3_] shows *T*_H_ = 80 K and *U*_eff_ = 1631(25) cm^−1^.^24^ Although smaller Cp^R^ ligands allow shorter Ln–L distances (and thus could improve *U*_eff_), they also permit less bulky [Dy(Cp^R^)_2_]^+^ cations to have more bent geometries, which leads to lower purity ±*m*_*J*_ states, faster Raman relaxation, and lower *T*_H_.^25,26^ Indeed, one of the smallest possible Cp^R^ ligands (Cp* = C_5_Me_5_) tends to form bent Dy(iii) bis-Cp* complexes with equatorially-bound ligands, which have all showed lower *U*_eff_ (<930(6) cm^−1^) and *T*_H_ (<12 K) values.^27,28^ Recently, we have explored the effect of weak equatorial binding of neutral halobenzenes to bent [Dy(Cp^R^)_2_]^+^ cations, finding that these ligands provide only minor disruption to the axiality if the complex geometry is preserved.^28,29^ We also found that the propensity for C–X (X = F, Cl, Br) bond activation of halobenzenes by [Dy(Cp^R^)_2_]^+^ cations increases with halogen size, indicating that fluoroarenes will provide the most stable haloarene-bound Ln cations.^28,29^ We hypothesised that a fluorobenzene adduct of a *mono*-Cp* Dy(iii) half-sandwich dication [Dy(Cp*)(FPh)_*n*_]^2+^ would show reduced *U*_eff_ due to having only one Cp^R^ ligand, but should have increased purity of low-lying ±*m*_*J*_ states *vs.* bent bis-Cp* cations and thus achieve a relatively high *T*_H_. Aside from magnetic properties, rare earth alkyl dications can be effective olefin polymerisation catalysts,^30^ but to the best of our knowledge the only previous structurally authenticated Ln(iii) mono-cyclopentadienyl dication piano stool complex with six “legs”^31^ was seen in [Tm(Cp*)(NCMe)_6_][I]_2_.^32^

The SMM properties of Ln sandwich complexes have been investigated extensively but half-sandwich Ln SMMs are comparatively rare.^22,23^ Examples of half-sandwich Dy(iii) Cp^R^ SMMs include [Dy(C_6_Me_6_)(Cl_4_Al)_3_],^33^ [Dy(C_7_H_8_)(X_4_Al)_3_] (X = Cl, Br),^34^ [Dy(Cp^R^)(DBM)_2_(THF)] (Cp^R^ = Cp*, C_5_^n^Pr_4_Ph or C_5_Me_4_SiMe_3_; DBM = dibenzoylmethanoate),^35^ [{Dy(Cp*)}_6_Cl_16_K_4_(THF)_6_],^36^ [Dy(Cp*)(DAD)(THF)] and [Dy(Cp*)(DAD){ClLi(THF)_3_}] (DAD = {(NArCMe)_2_}; Ar = Dipp, C_6_H_3_^i^Pr_2_-2,6);^37^ half-sandwich Dy(iii) complexes with other charge-dense aromatic rings include [{Dy(BH_4_)_2_(THF)}_2_(Fv^tttt^)] (Fv^tttt^ = 1,1′,3,3′-tetra-(*tert*-butyl)fulvalenyl),^38^ [Dy(^*t*^Bu_4_Carb)(*o*-CH_2_C_6_H_4_NMe_2_)_2_] (^*t*^Bu_4_Carb = 1,3,6,8-tetra-*tert*-butyl-9*H*-carbazole)^39^ and [Dy(BH_4_)_2_(THF){C_4_(SiMe_3_)_4_}M] (M = Na or K).^40^ Relatively charge-diffuse ligands such as cyclooctetraenyl (COT) are generally better-suited to Ln SMMs containing prolate Ln(iii) ions, such as in the mononuclear half-sandwich complexes [Tm(COT)(I)(THF)_2_],^41,42^ [Er{C_8_H_6_(Si^i^Pr_3_)_2_-1,4}(I)(THF)_2_],^43^ and [Er(COT)(X)(THF)_2_] (X = I, CH_2_Ph).^44^ Conversely, Gao and co-workers have shown that a combination of COT and monodentate ligands can give complex Ln CFs that may better-stabilise either prolate Er(iii) or oblate Dy(iii) ions in [Ln(COT)(BH_4_)(THF)_2_], [Ln(COT)(THF)_4_][BPh_4_], [Ln(COT)(OAr)(THF)_*n*_] (Ar = {C_6_H_2_(CHPh_2_)_2_-2,6-Me-4}, *n* = 2; Ar = {C_6_H_2_(Ad)_2_-2,6-Me-4}, Ad = adamantyl, *n* = 1) and [Ln{N(Si^i^Pr_3_)_2_}(COT)Na{N(Si^i^Pr_3_)_2_}].^45^ While this paper was under review,^46^ Gao, Wang and co-workers reported a series of related Dy(iii) mono-(imidazolin-2-iminato) complexes [Dy{N=C(NRCH)_2_}(sol)_5_][BPh_4_]_2_ (R = Dipp, sol = THF or pyridine; R = adamantyl, sol = THF).^47^

Here we report the half-sandwich Ln(iii) complexes [Ln(Cp*)(FPh)_6_] [{Al[OC(CF_3_)_3_]_3_}_2_(μ-F)]_2_ (1-Ln; Ln = Y, Dy) and their characterisation by elemental analysis, NMR and ATR-IR spectroscopy, powder and single crystal XRD, SQUID magnetometry, and density functional theory (DFT) and complete active space self-consistent field spin–orbit (CASSCF-SO) calculations. Despite the modest *U*_eff_ = 545(30) cm^−1^ for 1-Dy, its *T*_H_ = 14 K is similar to the best-performing Dy(iii) bis-Cp* complexes reported to date.^27,28^ These properties arise due to the relatively high CF splitting and purity of the most magnetic ±*m*_*J*_ states from one of the fluorobenzenes being effectively *trans*- to the Cp* and the other five being arranged nearly equally about the equatorial plane. Finally, we show that the salt metathesis reaction of 1-Y with KCp′′′ ({C_5_H_2_(SiMe_3_)_3_-1,3}) proceeds cleanly at room temperature. This provides proof-of-concept that 1-Ln and related complexes can also be useful starting materials to heteroleptic Ln complexes as weakly-bound haloarenes are easily displaced.^48^

## Results

### Synthesis

**Fig. 1:**
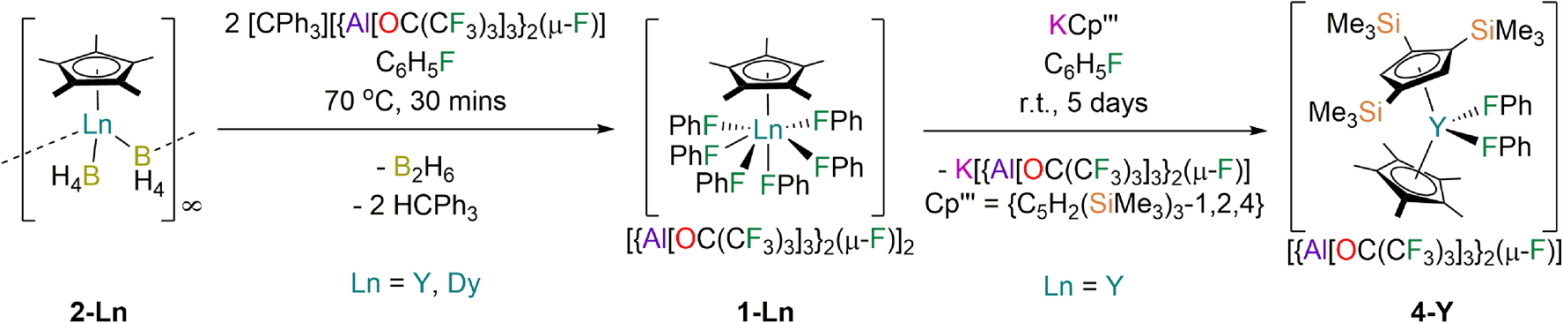
Synthesis of 1-Ln (Ln = Y, Dy) by the separate reactions of parent 2-Ln with [CPh_3_][{Al[OC(CF_3_)_3_]_3_}_2_(μ-F)] in fluorobenzene and synthesis of 4-Y by the reaction of 1-Y with KCp′′′ in fluorobenzene.

The solvent-free Ln(iii) Cp* borohydride precursors [Ln(Cp*)(μ-BH_4_)_2_]_∞_ (2-Ln; Ln = Y, Dy) were synthesised in 64–73% yields by the respective stoichiometric salt metathesis reactions of parent [Ln(BH_4_)_3_(THF)_3_]^49^ with KCp*^50^ in THF, followed by desolvation under reduced pressure (150 °C, 10^−3^ mbar) and recrystallisation from benzene or toluene. The abstraction of two hydrides from 2-Ln with concomitant installation of two weakly coordinating anions (WCAs) and elimination of diborane using two equivalents of [CPh_3_][{Al[OC(CF_3_)_3_]_3_}_2_(μ-F)]^51^ in fluorobenzene at 70 °C proceeded to completion in 30 min; the target complexes 1-Ln were isolated in 58–64% yields following work-up and recrystallisation from fluorobenzene solutions layered with hexane (Fig. 1). The relatively high thermal stability of 1-Ln towards C–F bond activation is remarkable. The more bulky WCA [{Al[OC(CF_3_)_3_]_3_}_2_(μ-F)]^−^ was required to stabilise the [Dy(Cp*)(FPh)_6_]^2+^ dication rather than the more commonly used WCA [Al{OC(CF_3_)_3_}_4_]^−^, as initial attempts to synthesise [Dy(Cp*)(FPh)_6_][Al{OC(CF_3_)_3_}_4_]_2_ from 2-Dy and [CPh_3_][Al{OC(CF_3_)_3_}_4_]^52^ under analogous conditions gave several crystals of a Dy(iii) bis-alkoxide byproduct, [Dy{OC(CF_3_)_3_}_2_(FPh)_5_][{Al[OC(CF_3_)_3_]_3_}_2_(μ-F)] (3-Dy); the mechanism of formation of 3-Dy is unknown but likely proceeds *via* the abstraction of {OC(CF_3_)_3_} from [Al{OC(CF_3_)_3_}_4_]^−^ to generate the WCA, promoted by the Lewis acidic Dy(iii) centre. As only a trace amount of 3-Dy was isolated its characterisation was limited to single crystal XRD. We have previously investigated the substitution of different monohalobenzenes in the Dy(iii) bent cations, [Dy(Cp^R^)_2_(XPh)_*n*_]^+^ (Cp_2_^R^ = Cp^ttt^/Cp*, X = F, Cl, Br, *n* = 1; Cp^R^_2_ = Cp*_2_, X = F, Cl, *n* = 2).^28,29^ As the propensity for decomposition by C–X bond activation in these complexes was found to increase with halogen size we assumed that similar processes will occur for heavier halobenzenes for more reactive [Dy(Cp*)(XPh)_*n*_]^2+^ dications; this precludes the synthesis of mixed halobenzene complexes, and we therefore limit our study here to fluorobenzene only.

We envisaged that 1-Ln could be useful precursors to novel heteroleptic Ln complexes as the coordinated fluorobenzenes will be easily displaced in salt metathesis reactions,^48^ allowing a second bulky anionic ligand to be installed without the need for forcing conditions that can lead to byproduct formation. *e.g.* by cyclometallation.^53^ We have previously shown that Ln complexes coordinated by Cp′′′ are prone to decomposition reactions with anion abstraction reagents due to facile cleavage of the weak C–Si bonds, thus isolated Ln metallocenium cations were previously unknown for this ligand.^54^ Gratifyingly, the salt metathesis reaction of 1-Y with one equivalent of KCp′′′^55^ in fluorobenzene proceeded at room temperature to give [Y(Cp′′′)(Cp*)(FPh)_2_][{Al[OC(CF_3_)_3_]_3_}_2_(μ-F)] (4-Y) in 57% yield following work-up and recrystallisation from a mixture of fluorobenzene and *n*-hexane (Fig. 1). It is evident that a larger Cp^R^ ligand than Cp* would be needed to prevent halobenzene coordination to give a high-performance SMM such as a solvent-free [Dy(Cp^R^)_2_]^+^ cation, but the reaction to form 4-Y provides proof-of-concept that this synthetic approach provides a useful alternative to salt metathesis reactions with an inner-sphere halide or borohydride anion.

### Bulk solid-state characterisation

Lower carbon values than expected were reproducibly obtained in elemental analysis results of 1-Ln and 2-Ln. This was ascribed to a combination of issues intrinsic to this technique,^56^ and the experimental conditions employed leading to carbide formation,^57^ which is particularly common for fluorine-rich complexes;^58^ the lower than expected hydrogen values seen for 1-Y was also attributed to its high fluorine content. The other analytical data collected for 1-Ln and 2-Ln are in accord with the bulk purity of these samples. The ATR-IR spectra of 1-Ln are essentially superimposable and are dominated by signals corresponding to the WCA (see ESI Fig. S1 and S2†). In contrast the ATR-IR spectra of 2-Dy and 2-Y are dissimilar due to the presence of crystals of 2-Dy·0.33C_6_H_5_CH_3_ identified in batches of the former, but characteristic bridging and terminal B–H stretching modes could be assigned (see ESI Fig. S3 and S4†).^59^ The ATR-IR spectrum of 4-Y is unremarkable (see ESI Fig. S5†), with the majority of peaks being attributable to the WCA.

The DFT-calculated spectrum of 1-Y shows that the expected C–F stretches (1106, 767 and 569 cm^−1^) are all observed (see ESI Fig. S6†). The bulk phase purity of 1-Dy was further demonstrated by powder XRD (see ESI Fig. S7, S8 and Table S2†), as the diffraction pattern observed was in excellent agreement with that expected from single crystal XRD data (see below). Le Bail refinement gave reduced unit cell parameters, indicating that one molecule of hexane in the lattice per unit cell is lost upon preparation of samples of 1-Dy for analysis; this could further account for the comparatively low carbon and hydrogen values obtained, but due to experimental uncertainties we do not adjust formula weights for the analysis of magnetic data (see below).

### Solution characterisation

Multinuclear NMR spectroscopic data was collected on solutions of 1-Ln, 2-Ln and 4-Y (see ESI Fig. S9–S19†). Complexes 1-Ln spontaneously form biphasic solutions in fluorobenzene, and this thwarted the collection of meaningful solution NMR data as other weakly coordinating polar solvents that can dissolve 1-Ln including other halobenzenes will coordinate to the Ln(iii) ions and displace the coordinated fluorobenzene.^29^ Attempts were made to collect NMR data on fluorobenzene solutions of 1-Ln at elevated temperatures (>70 °C) but this led to sample decomposition, presumably due to C–F activation.

The ^1^H NMR spectrum of 2-Y in C_6_D_6_ contains a singlet at 2.06 ppm integrating to 15H for the methyl groups, and a broad quartet at 0.66 ppm for the 8H corresponding to the borohydrides; the ^13^C{^1^H} NMR spectra also only shows two signals for the CH_3_ (11.7 ppm) and quaternary C (123.5 ppm) environments, with no residual THF observed. The borohydride signal in the ^1^H NMR spectrum is a broad quartet (^1^*J*_BH_ = 84.6 Hz) from coupling to 80.1% abundant *I* = 3/2 ^11^B nuclei, with the coupling to 100% abundant *I* = 1/2 ^89^Y nuclei not resolved; these assignments were confirmed by ^1^H{^11^B}, ^11^B{^1^H} and ^11^B NMR spectra, with the latter showing the expected pentet at −22.0 ppm from coupling to four ^1^H nuclei. The NMR spectra of 2-Y are comparable to those of [Y(Cp*)_2_(μ-BH_4_)]_∞_,^28^ indicating that there is dynamic exchange on the NMR timescale. The paramagnetism of 2-Dy precluded the observation of signals by ^11^B, ^11^B{^1^H} and ^13^C{^1^H} NMR spectroscopy, whilst only one broad signal was observed in the ^1^H NMR spectrum of a C_6_D_6_ solution at −57.65 ppm (FWHM ≈ 5100 Hz) that we tentatively assign to the Cp* protons. The Evans method^60^ was used to determine the magnetic susceptibility of 2-Dy in C_6_D_6_ solution (10.46 *μ*_B_), which is close to the expected value for a Dy(iii) ion (10.50 *μ*_B_).^61^ The NMR data for 4-Y are in accord with the expected spectra for this complex.

### Single crystal XRD

**Fig. 2:**
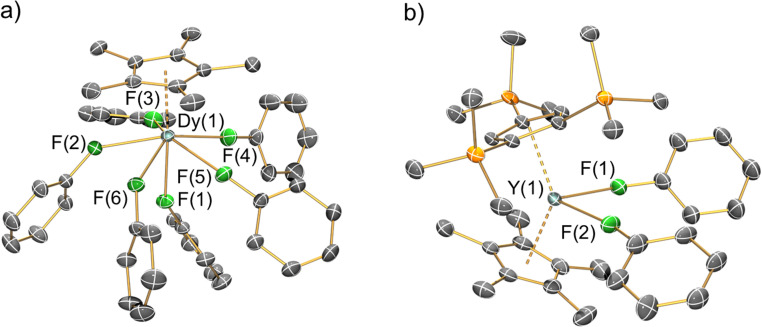
Single crystal XRD structures of (a) the dication of 1-Dy and (b) the cation of 4-Y with selected atom labelling (Y/Dy: cyan, C: grey, F: green, Si: orange). Displacement ellipsoids set at 20% probability levels; hydrogen atoms, the lattice solvents and the counteranions have been omitted for clarity. See ESI† for selected bond lengths and angles.

The solid-state structures of 1-Ln, 2-Ln, 3-Dy and 4-Y were determined by single crystal XRD (see Fig. 2 for depictions of the dication in 1-Dy and the cation in 4-Y; the structures of 1-Y, 2-Ln and 3-Dy are compiled in the ESI Fig. S20–S24,† together with selected crystallographic parameters in Tables S3–S6†). In the solid state the mono-ring complexes 2-Ln are bridged by BH_4_ groups to give oligomers that are comparable to [Y(Cp*)_2_(μ-BH_4_)]_∞_;^29^ hexameric 2-Dy·0.33C_6_H_5_CH_3_ was also identified and the structure is reminiscent of [La(Cp^ttt^)(μ-BH_4_)_2_]_6_ ^62^ and [Ln(C_5_Me_4_^n^Pr)(μ-BH_4_)_2_]_6_ (Ln = Nd, Sm).^63^ The {OC(CF_3_)_3_} substituents of the [{Al[OC(CF_3_)_3_]_3_}_2_(μ-F)]^−^ WCAs are highly disordered in all datasets, which strongly influences the statistics for the fit of the models, especially to the weaker higher angle data. This disorder cannot be fully modelled while ensuring an appropriate data to parameter ratio for the given resolution of the datasets, and so the overall fit to the data appears poor. This is a known problem for crystal systems containing this WCA and related examples such as [Al{OC(CF_3_)_3_}_4_]^−^, as evidenced by papers referenced herein.^51,52^ These previously reported datasets generally have high *R*_1_ and w*R*_2_ values, even when the data are modelled effectively and low goodness of fits are obtained. Plots of electron density maps (see ESI Fig. S25–S32†) derived from the data show that the models fit well and produce appropriate phases for the observed structure factors. Plots of 2*F*_o_ − *F*_c_ show good agreement of the model of the main molecule with the electron density map, with clear deficiencies around the WCAs. Plots of *F*_o_ − *F*_c_ difference maps confirm the main deficiencies are around the WCA. This is indicative that the model is appropriate, especially for the cations of interest, for the given resolution and size of the dataset. The noise around the heavy metal sites in the *F*_o_ − *F*_c_ difference maps is common when collecting data for compounds containing heavy metals using CuKα radiation; the small size of the crystals precluded the use of a lower brilliance shorter wavelength MoKα source when collecting the data. This also limits the maximum resolution of the dataset that can be obtained, limiting the extent that disorder of the WCAs can be modelled to improve the fit.

Despite the highly disordered WCAs in 1-Ln giving inherently high *R*-factors for these datasets, the dications are relatively well-resolved, as are the lattice fluorobenzene and hexane molecules, with minimal structural disorder. The [Ln(Cp*)(FPh)_6_]^2+^ dications in 1-Ln exhibit six-legged piano stool geometries, with a Cp* “seat” and six monodentate fluorobenzene “legs” each binding *via* F lone pairs. These half-sandwich dications can alternatively be described as showing distorted *pseudo*-pentagonal bipyramidal geometries, with one F atom essentially *trans*- to the Cp* centroid (Cp*_centroid_⋯Dy–F = 176.20(9)°) and the five equatorial F atoms forming a plane with mean F–Dy–F angles of 69.65(4)°; the deviation from ideal 72° is due to the Dy atom being 0.570(2) Å above the F_5_ plane. In this regard the structures of 1-Ln are reminiscent of archetypal pentagonal bipyramidal Dy(iii) SMMs, of which complexes with two apical alk-/aryl-oxides and five equatorial neutral ligands are the most prevalent;^64^ the Dy(iii) mono-aryloxide mono-halide complexes [Dy(OR)(X)(THF)_5_][BPh_4_] (R = CMe_3_, SiMe_3_, Ph; X = Cl, Br) are arguably the closest examples to compare with 1-Ln.^65^ There is a wide range of Cp*_centroid_⋯Dy–F_eq_ (101.65(10)–106.54(14)°), F_eq_–Dy–F_eq_ (73.94(12)–78.67(13)°), F_eq_–Dy–F_eq_ (68.43(12)–70.8(2)°) and Dy–F–C_*ipso*_ (139.3(3)–166.6(4)°) angles in 1-Dy, due to a combination of non-directional predominantly ionic bonding regimes and crystal packing effects.

The Dy⋯Cp*_centroid_ distance (2.2737(4) Å) and mean Dy–F (2.402(2) Å) and F–C_*ipso*_ (1.420(3) Å) bond lengths of 1-Dy are similar to those of the only two other structurally characterised fluorobenzene Dy complexes [Dy(Cp^ttt^)(Cp*)(FPh)][Al{OC(CF_3_)_3_}_4_] (Dy⋯Cp*_centroid_ = 2.315(2) Å; Dy–F = 2.429(2) Å; F–C_*ipso*_ = 1.414(4) Å)^29^ and [Dy(Cp*)_2_(FPh)_2_][Al{OC(CF_3_)_3_}_4_] (mean Dy⋯Cp*_centroid_ = 2.286(2) Å; mean Dy–F = 2.358(12) Å; mean F–C_*ipso*_ = 1.42(3) Å).^28^ The only previous structurally authenticated six-legged piano stool complex to our knowledge is [Tm(Cp*)(NCMe)_6_][I]_2_,^32^ which exhibits Cp*_centroid_⋯Tm–N (176.5(2)° for the axial NCMe; mean 102.5(2)° for the equatorial NCMe) and N–Tm–N (69.8(2)° mean between equatorial NCMe; 77.5(2)° between equatorial and axial NCMe) angles that are comparable to the respective Cp*_centroid_⋯Dy–F and F–Dy–F angles in 1-Dy. Finally, the solid-state structure of the cation of 4-Y is comparable to that seen in [Dy(Cp*)_2_(FPh)_2_][Al{OC(CF_3_)_3_}_4_], albeit with longer Ln⋯Cp^R^_centroid_ distances (Y⋯Cp′′′_centroid_: 2.3100(4) Å; Y⋯Cp*_centroid_: 2.3703(4) Å; Dy⋯Cp*_centroid_: 2.2763(8) and 2.2957(7) Å) and a more bent Cp^R^_centroid_⋯Ln⋯Cp^R^_centroid_ geometry (4-Y: 138.49(2)°; [Dy(Cp*)_2_(FPh)_2_][Al{OC(CF_3_)_3_}_4_]: 142.49(4)°).^28^

### Magnetism

**Fig. 3:**
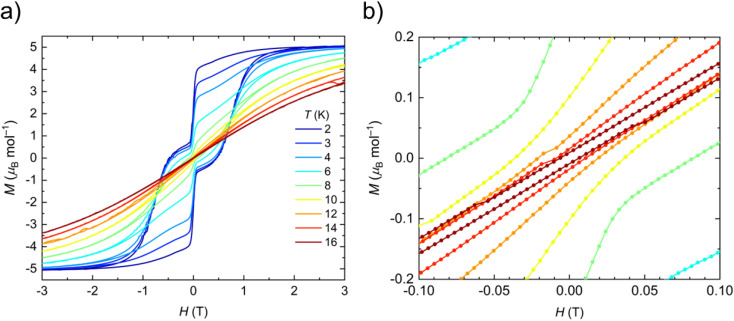
(a) Hysteresis loops of 1-Dy from 2 to 16 K and −3 T to +3 T, sweep rate is 22 Oe s^−1^. (b) Hysteresis loops of 1-Dy at 6–16 K between −3 and +3 T zoomed in between −0.1 and +0.1 T, sweep rate is 22 Oe s^−1^. Hysteresis is considered open until *T*_H_ = 14 K.

The static and dynamic magnetic properties of polycrystalline 1-Dy in the solid-state were determined by SQUID magnetometry (see ESI Fig. S33–S39 and Tables S7 and S8†). The molar magnetic susceptibility–temperature product *χT* is 14.0 cm^3^ K mol^−1^ at room temperature, similar to that predicted for a free Dy(iii) ion (^6^H_15/2_, *χT* = 14.2 cm^3^ K mol^−1^)^61^ and the CASSCF-SO-calculated value (see ESI† Fig. S33†). There is minimal variation in *χT* with temperature until 13 K, at which point a sharp decline is observed that is typical of magnetic blocking. Open magnetic hysteresis loops are present at 2 K and remain open up to *T*_H_ = 14 K at a sweep rate of 22 Oe s^−1^ in the zero-field region (Fig. 3), with a large drop at zero-field indicating efficient QTM. It is remarkable that 1-Dy with only one anionic ligand has similar *T*_H_ to the best-performing bis-Cp* Dy(iii) SMMs with weak equatorial ligands, *e.g.* [{Dy(Cp*)_2_(μ-(Me)_2_AlMeNEt_3_)}_2_][Al{OC(CF_3_)_3_}_4_]_2_ (12 K)^27^ and [Dy(Cp*)_2_(XPh)_2_][Al{OC(CF_3_)_3_}_4_] (X = F, 8 K; X = Cl, 10 K).^28^*T*_H_ values of 22–24 K were reported for the mono-halobenzene-bound Dy(iii) bis-Cp^R^ complexes [Ln(Cp^ttt^)(Cp*)(PhX-κ-X)][Al{OC(CF_3_)_3_}_4_] (X = F, Cl, Br)^29^ and *T*_H_ = 80 K was seen for the best-performing dysprosocenium SMM [Dy(C_5_^i^Pr_5_)(Cp*)][B(C_6_F_5_)_4_];^15^ a new record *T*_H_ of 100 K has recently been claimed for a Dy(iii) bis-amide complex.^66^ We posit that the comparable relaxation dynamics of 1-Dy to these literature examples is due to its ±*m*_*J*_ states being more pure from not having a second anionic ligand introducing strong transverse fields, despite the smaller axial CF imposed by only one Cp* ligand (see below). As stated above one of the fluorine donor atoms is effectively *trans*- to the Cp*_centroid_, and the five other fluorine donor atoms are arranged about the equatorial positions of a *pseudo*-pentagonal bipyramid; this arrangement of ligands is more symmetrical than in the bent bis-Cp* Dy(iii) complexes in the literature, which should lead to smaller transverse fields and relatively pure ±*m*_*J*_ states (see below). The *T*_H_ of 1-Dy also exceeds that of the aforementioned pentagonal bipyramidal Dy(iii) complexes [Dy(OR)(X)(THF)_5_][BPh_4_] (R = CMe_3_, SiMe_3_, Ph; X = Cl, Br), which show *T*_H_ values between 9 and 11 K depending on the substituents.^65^ As the hysteresis behaviour of 1-Dy is in accord with the extracted relaxation rates there is no need to perform magnetic studies on a dilute sample doped into a matrix of 1-Y to prove that this property is unimolecular rather than the result of cohesive intermolecular interactions.

**Fig. 4:**
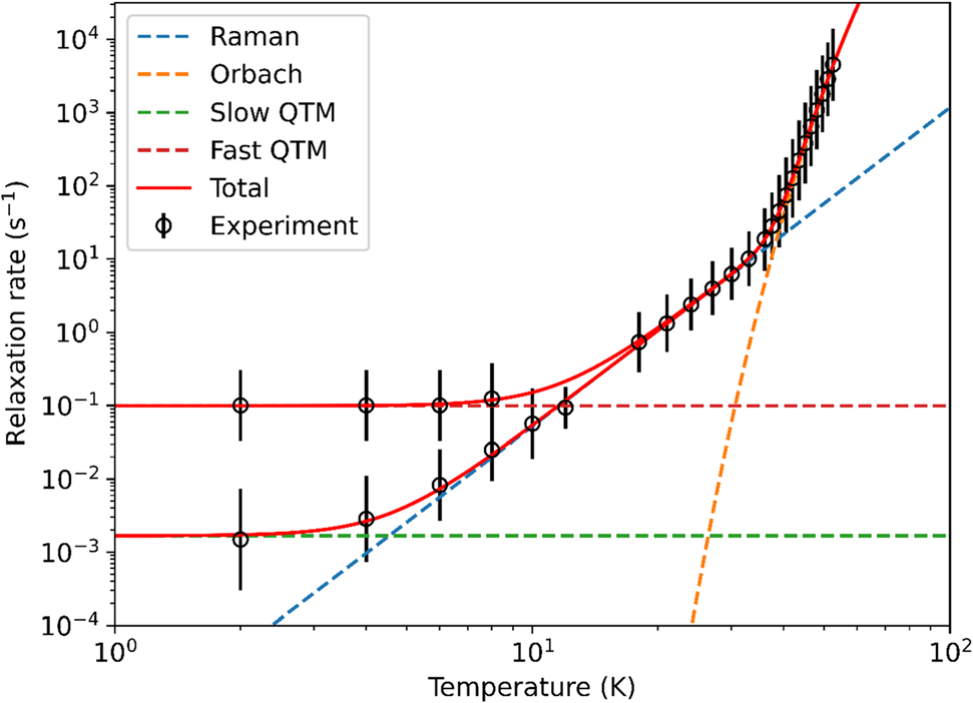
Fitting of 1-Dy relaxation profile, showing Orbach, Raman and QTM components derived from eqn (S5).† Error bars represent one ESD in the distribution of rates.

Peaks were observed in zero-field out-of-phase AC susceptibility data for 1-Dy at temperatures between 18–52.5 K, and these data fitted well to generalized Debye models (fit with CC-FIT2,^67,68^ see ESI Fig. S34, S35 and Table S7†). To determine the low temperature relaxation dynamics, magnetisation decay measurements of 1-Dy were performed in zero field between 2–12 K (see ESI Fig. S36 and S37†). The decays at *T* ≤ 10 K clearly indicate two different relaxation timescales in 1-Dy, necessitating a double stretched exponential model (eqn (S2)†),^69^ while data at *T* = 12 K exhibits only single stretched exponential decay (eqn (S3)†). To confirm the presence of two relaxation pathways we performed waveform measurements^70^ on 1-Dy. There are two distinct peaks in the extracted out-of-phase susceptibility data at 2 K (Fig. S38†). As the temperature increases the low frequency peak moves to the right and coalesces with the high frequency peak, which remains constant throughout, at 10 K (Fig. S38†). The data at *T* ≤ 8 K were modelled with a double generalised Debye model, while the data at *T* = 10, 12 K were modelled with a single generalised Debye model (Fig. S38†). The waveform data are in reasonable agreement with the rates extracted from DC decays (Fig. S39†), however showing slightly faster rates for the slower relaxation process with much smaller distributions. This is not uncommon for extremely long relaxation times where the DC decay method makes too many assumptions on the underlying data.^68^ The relaxation profile of 1-Dy (Fig. 4) shows clear exponential Orbach relaxation at high-temperatures, switching to a power-law Raman process below *τ*_switch_ = 18 s at 39 K.^71^ At low temperatures, there are two clear relaxation processes. The first has faster rates showing temperature-independent relaxation dynamics indicative of QTM. The second exhibits slower rates that follows the higher-temperature Raman profile before appearing to plateau around 2 K, indicating the onset of a QTM dominated regime with a slower rate. The fraction of the two processes is approximately 2.5 : 1 in favour of the slow relaxation rates. The relaxation profile was fitted to a model with Orbach 
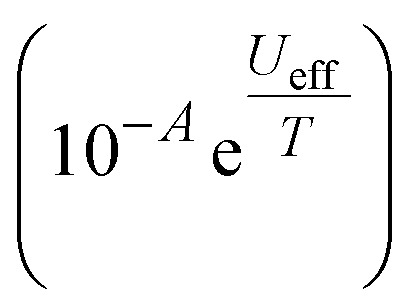
, Raman (10^R^*T*^*n*^) and QTM (10^−*Q*^) contributions (eqn (S5)†), with two different QTM contributions to account for the low temperature data. The resulting fitted parameters are *U*_eff_ = 545(30) cm^−1^, *A* = −10.12(19) log_10_[s], *R* = −5.63(7) log_10_[s^−1^ K^−*n*^], *n* = 4.35(6), *Q*_fast_ = 1.004(7) log_10_[s] and *Q*_slow_ = 2.77(6) log_10_[s], with the model and experimental data in excellent agreement. Again these values are comparable with those of the family of Dy(iii) mono-aryloxide complexes [Dy(OR)(X)(THF)_5_][BPh_4_] (R = CMe_3_, SiMe_3_, Ph; X = Cl, Br), where *U*_eff_ values vary between 354(34)–453(72) cm^−1^.^65^ The largest *U*_eff_ value for a Dy(iii) bis-Cp* complex to date (930(6) cm^−1^) was seen for [Dy(Cp*)_2_(FPh)_2_][Al{OC(CF_3_)_3_}_4_],^28^ with the current record-holding dysprosocenium SMM showing *U*_eff_ = 1541 cm^−1^.^15^

### *Ab initio* calculations

**Fig. 5:**
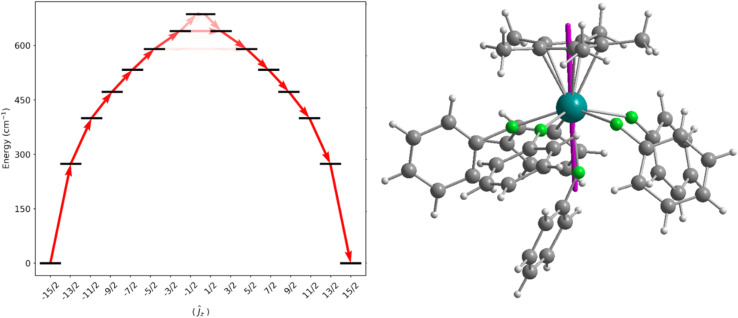
Energy barrier to magnetic relaxation for a model of [Dy(Cp*)(FPh)_6_]^2+^. Electronic states from CASSCF-SO calculations, labelled with their dominant *m*_*J*_ composition in the *J* = 15/2 basis. Arrows represent the Orbach relaxation pathway, where the opacity of the arrows is proportional to the transition probability approximated with the average matrix elements of magnetic moment connecting the states, *γ*_ij =_ (1/3)[|〈*i*|*μ*_*x*_|*j*〉|^2^ + |〈*i*|*μ*_*y*_|*j*〉|^2^ + |〈*i*|*μ*_*z*_|*j*〉|^2^], normalized from each departing state and commencing from |−15/2〉 (left). Denotation of the *g*_*z*_ axis (purple) within the solid-state structure at the ground state (right).

CASSCF-SO calculations were performed on molecular structures obtained from single crystal XRD studies (see ESI† for details). We approximate a calculated *U*_eff_ for the dication of 1-Dy as the energy of the first Kramers doublet with significant transverse *g*_*x*_ and/or *g*_*y*_ values (>1), giving 589 cm^−1^ at the 5^th^ excited state (Fig. 5 and Table S9†), which is in excellent agreement with the measured data. The ground state *g*_*z*_ value is aligned along Cp*_centroid_⋯Dy–F_ax_, and the CF states up to the 5^th^ excited state are essentially pure ±*m*_*J*_ functions due to the lack of strong off-axis interactions and the relatively symmetrical distribution of the five equatorially-bound fluorobenzenes, such that their contribution to the anisotropy axis cancels;^72,73^ the retention of purity is reminiscent of the predicted electronic structure of the theoretical [DyO]^+^ cation.^6^ As stated above the *U*_eff_ and *T*_H_ of 1-Dy are similar to the range of values (*U*_eff_ = 354(34)–453(72) cm^−1^ and *T*_H_ = 9–11 K) observed for the distorted pentagonal bipyramidal Dy(iii) complexes [Dy(OR)(X)(THF)_5_][BPh_4_] (R = CMe_3_, SiMe_3_, Ph; X = Cl, Br), but in those cases Orbach magnetic relaxation proceeds *via* only the second excited state,^65^*versus* the 5^th^ excited state here. Similar trends have been seen for the aforementioned Dy(iii) mono-(imidazolin-2-iminato) complexes [Dy{N=C(NRCH)_2_}(sol)_5_][BPh_4_]_2_, which exhibit *U*_eff_ values between 614–860 cm^−1^ and *T*_H_ from 7–14 K.^47^

We have previously investigated the effects of equatorial halobenzene binding on the CF of SMMs in the Dy(iii) bent cations, [Dy(Cp^R^)_2_(XPh)_*n*_]^+^, and we showed that halogen substitution changes the magnitude of the transverse field.^28,29^ To understand how the fluorobenzene ligands affect the magnetic anisotropy of 1-Dy here, we performed CASSCF-SO calculations on model complexes using the metrical parameters from the single crystal XRD structure of 1-Dy as a starting point (see ESI Fig. S40–S43 and Tables S9–S13†). The calculated *U*_eff_ values of hypothetical [Dy(Cp*)]^2+^ and [Dy(Cp*)(FPh)]^2+^ dications, with only the axially-bound fluorobenzene retained in the latter, are 1071 cm^−1^ and 1505 cm^−1^, respectively. This highlights the constructive role of the axially-bound *trans*-fluorobenzene ligand that can vastly increase *U*_eff_*cf.* [Dy(Cp*)]^2+^, as well as the destructive role of the equatorial fluorobenzene ligands that reduce *U*_eff_ down to 589 cm^−1^. While these significant changes in *U*_eff_ with different dispositions of neutral solvent molecules clearly demonstrates the shortcomings of the classical electrostatic model, the observation that the equatorial neutral solvent molecules do not significantly mix the ±*m*_*J*_ states is remarkable.^72,73^ Although the [Dy{OC(CF_3_)_3_}_2_(FPh)_5_]^+^ cation of 3-Dy contains weakly-bound fluorinated alkoxides, its predicted *U*_eff_ value (1275 cm^−1^) is competitive with many axial Dy(iii) alk/aryloxide SMMs with more strongly donating axial and equatorial ligands^64^ (current record *U*_eff_ = 1687 cm^−1^ for [Dy(OAd)_2_(18-crown-6)][I_3_],^74^ though a preprint on a Dy(iii) bis-amide SMM reports *U*_eff_ = 1843(11) cm^−1^ (ref. 66)) thus the deliberate synthesis of haloarene analogues of ether- and pyridine-bound axial Dy alk/aryloxide complexes should also be worthwhile targets for the SMM community. Finally, the *U*_eff_ value for the [Dy(Cp′′′)(Cp*)(FPh)_2_]^+^ cation in the Dy analogue of 4-Y is predicted to be 674 cm^−1^, due to a combination of its bent Cp′′′_centroid_⋯Dy⋯Cp*_centroid_ motif and equatorially-bound fluorobenzenes; 4-Dy was not targeted as it would not give notable SMM properties compared to other leading Dy(iii) bis-Cp^R^ SMMs.^13–16,29^

**Fig. 6:**
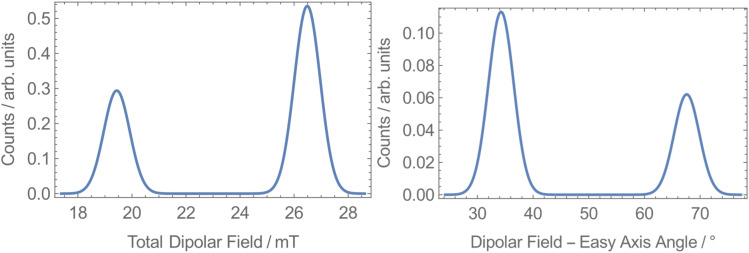
Dipolar field calculations for 1-Dy at all sites showing the dipolar field strength (left) and angle to the easy-axis (right) at a cutoff radius of 160 Å.

To explore the origin of the two different QTM rates, we calculated the dipolar fields at each unique Dy site in 1-Dy. The space group *P*2_1_/*n* has 4 molecules in the unit cell, with two Dy sites lying on the edge (1 and 3 in Fig. S44†) and two within the cell (2 and 4 in Fig. S44†). Sites 1 and 3 (and 2 and 4) are related by inversion, while sites 1 and 2 (and 3 and 4) are related by a C_2_ rotation, which has been shown to give rise to different local dipolar fields.^75^ We built a model of the crystal structure containing unit cells up to a cutoff radius of 160 Å from a reference central cell and then selected one of the four Dy sites inside the reference cell. Assuming all molecules within the cutoff radius were fully magnetised along their easy-axes in the direction of an external field, we calculated the total dipolar field at the selected Dy site in the reference cell. This was repeated for many random orientations of the lattice relative to the external field and for the other three Dy sites in the reference cell. We find that at each site the distribution of dipolar fields is bimodal: 27 mT at 34° from the easy-axis and 19 mT at 68° from the easy-axis (Fig. 6). While our model is too crude to directly predict the different QTM rates, the presence of two different internal dipolar fields is consistent with the observation of two distinct QTM rates.

## Conclusion

To summarise, we have synthesised and characterised a new class of half-sandwich Ln SMMs, where the magnetic anisotropy and resultant magnetic properties are dominated by only one anionic ligand. A relatively modest *U*_eff_ value is found for 1-Dy as it only possesses one anionic Cp* ligand in the fluorobenzene-bound half-sandwich Dy(iii) dication [Dy(Cp*)(FPh)_6_]^2+^. The fluorine atom of one of the neutral fluorobenzene ligands is effectively *trans*- to the Cp*_centroid_, which contributes to the axial CF whilst introducing a smaller transverse CF than that imposed by the second Cp* ligand in bent bis-Cp* Dy(iii) SMMs. *Ab initio* studies of theoretical [Dy(Cp*)]^2+^ and [Dy(Cp*)(FPh)]^2+^ dications confirm that the *U*_eff_ of 1-Dy is enhanced by the axial fluorobenzene and diminished by the equatorial fluorobenzenes, but, crucially, the transverse CF introduced by the five equatorially-bound fluorobenzenes is weak and the ±*m*_*J*_ states maintain high purity. As a result, 1-Dy shows open magnetic hysteresis up to 14 K, which is comparable to the best-performing Dy(iii) bis-Cp* complexes reported to date^27,28,76–78^ and the most similar pentagonal bipyramidal Dy(iii) complexes, *e.g.* [Dy(OR)(X)(THF)_5_][BPh_4_] (R = CMe_3_, SiMe_3_, Ph; X = Cl, Br),^63^ as well as the related Dy(iii) mono-(imidazolin-2-iminato) complexes [Dy{N=C(NRCH)_2_}(sol)_5_][BPh_4_]_2_.^47^ We have also demonstrated the facile displacement of fluorobenzene from 1-Y in a salt metathesis reaction to give a Ln metallocenium cation, [Y(Cp′′′)(Cp*)(FPh)_2_]^+^, that could not be isolated by standard abstraction methodologies. We envisage that extending the synthetic methods herein to a wider range of [Ln(L)(XPh)_*n*_]^2+^ dications (L = anionic ligand, X = halogen) will provide complexes with interesting properties, which can be exploited as useful starting materials to hitherto unknown heteroleptic Ln complexes, including high temperature Ln SMMs with no equatorially-bound ligands, such as solvent-free [Dy(Cp^R^)_2_]^+^ cations.

## Methods

### General

All manipulations were performed in an inert argon atmosphere with rigorous exclusion of oxygen and water using Schlenk line and glovebox techniques. The solvents *n*-hexane, toluene and benzene were dried by refluxing over potassium and stored over potassium mirrors. Pentane was dried by refluxing over NaK and stored over a potassium mirror. Fluorobenzene and *ortho*-difluorobenzene were dried by refluxing over CaH_2_ and was stored over 4 Å molecular sieves. Tetrahydrofuran (THF) was dried by refluxing over potassium and stored over 4 Å molecular sieves. All solvents were degassed before use. The starting materials [Y(BH_4_)_3_(THF)_3_],^49^ [Dy(BH_4_)_3_(THF)_3_],^49^ KCp*,^50^ KCp′′′^55^ and PhF-Al{OC(CF_3_)_3_}_3_ ^79^ were prepared according to literature methods. [CPh_3_][{Al[OC(CF_3_)_3_]_3_}_2_(μ-F)] was synthesised by a modification of the published procedure.^51^ The reagent [CPh_3_][PF_6_] was purchased from Sigma-Aldrich and was used as received.

^1^H (400 MHz), ^13^C (126 MHz), ^11^B (128 MHz),^19^F (376 MHz) and ^29^Si DEPT90 (80 MHz) NMR spectra were obtained on a Bruker Avance III 400 or 500 MHz spectrometer at 298 K and were referenced to the solvent used, or to external SiMe_4_ (^1^H, ^13^C, ^29^Si), H_3_BO_3_/D_2_O (^11^B) or C_7_H_5_F_3_/CDCl_3_ (^19^F). ATR-IR spectra were recorded on a Bruker Alpha spectrometer with Platinum-ATR module. Elemental analysis was carried out by Mr Martin Jennings and Mrs Anne Davies at the Microanalytical Service, Department of Chemistry, The University of Manchester. NMR and ATR-IR spectra are compiled in the ESI,† together with details of single crystal and powder XRD, SQUID magnetometry, and DFT and CASSCF-SO calculations.

### Preparation of [Y(Cp*)(FPh)_6_][{Al[OC(CF_3_)_3_]_3_}_2_(μ-F)]_2_ (1-Y)

Fluorobenzene (30 mL) was added to a mixture of 2-Y (0.508 g, 2.0 mmol) and [CPh_3_][{Al[OC(CF_3_)_3_]_3_}_2_(μ-F)] (6.906 g, 4.0 mmol). The reaction mixture was heated to 70 °C and stirred for 30 min. The solution was concentrated *in vacuo* to 5 mL; *n*-hexane (50 mL) was then added, and the mixture vigorously stirred to form a yellow oil. The supernatant was decanted and fluorobenzene (30 mL) was added to the crude product; the mixture was stirred for 10 min. The product collected as the bottom layer of a biphasic solution; the top layer was decanted, and the addition of fluorobenzene and separation of the layers was repeated. The remaining yellow solution was layered with *n*-hexane (30 mL) and stored at room temperature to give bright yellow crystals. The supernatant was decanted, and residual solvent removed to give 1-Y (5.002 g, 1.3 mmol, 64%). Anal. calcd for C_206_H_123_Al_8_F_233_O_24_Y_2_: C, 31.71; H, 1.59. Found: C, 29.23; H, 0.96. The formation of biphasic solutions in fluorobenzene together with rapid fluorobenzene exchange dynamics precluded the collection of meaningful solution NMR data. FTIR (ATR, microcrystalline): *ν̃* = 2964 (w, C–H stretch), 2875 (w, C–H stretch), 1585 (w), 1486 (m), 1453 (w), 1354 (m, C–O stretch), 1301 (s), 1274 (s, C–F stretch), 1239 (s), 1211 (s), 1174 (s), 1116 (m), 1106 (s, C–F stretch), 1067 (w), 1019 (w), 970 (s), 896 (w), 863 (m), 808 (w), 767 (m, C–F stretch), 748 (s), 725 (s), 705 (w), 674 (w), 635 (m), 569 (m, C–F stretch), 536 (s), 471 (w), 450 (s) cm^−1^.

### Preparation of [Dy(Cp*)(FPh)_6_][{Al[OC(CF_3_)_3_]_3_}_2_(μ-F)]_2_ (1-Dy)

Complex 1-Dy was prepared following analogous synthetic and work-up procedures to 1-Y from 2-Dy (0.537 g, 1.5 mmol) and [CPh_3_][{Al[OC(CF_3_)_3_]_3_}_2_(μ-F)] (5.179 g, 3.0 mmol). The product 1-Dy was isolated as orange crystals (2.273 g, 0.6 mmol). A second crop of 1-Dy was obtained from the combined fluorobenzene supernatant solutions (1.162 g, 0.3 mmol). Total yield = 3.435 g, 0.9 mmol, 58%. Anal. Calcd for C_206_H_123_Al_8_Dy_2_F_233_O_24_: C, 31.12; H, 1.56. Found: C, 28.61; H, 1.33. The formation of biphasic solutions in fluorobenzene together with rapid fluorobenzene exchange dynamics and the paramagnetism of 1-Dy precluded the collection of meaningful solution NMR data. FTIR (ATR, microcrystalline): *ν̃* = 2955 (w, C–H stretch), 2929 (w, C–H stretch), 1585 (w), 1486 (m), 1453 (w), 1354 (m, C–O stretch), 1301 (s), 1268 (s, C–F stretch), 1241 (s), 1211 (s), 1176 (s), 1118 (m), 1108 (s, C–F stretch), 1067 (w), 1019 (w), 970 (s), 896 (w), 861 (m), 834 (w), 808 (w), 769 (m, C–F stretch), 748 (s), 725 (s), 705 (w), 676 (w), 635 (m), 569 (m, C–F stretch), 536 (s), 485 (w), 450 (s) cm^−1^.

### Preparation of [Y(Cp*)(BH_4_)_2_]_∞_ (2-Y)

THF (40 mL) was added to a mixture of [Y(BH_4_)_3_(THF)_3_] (2.099 g, 6.0 mmol) and KCp* (1.046 g, 6.0 mmol), and the reaction mixture was stirred at room temperature for 48 h. Following the removal of THF under vacuum, toluene (40 mL) was used to extract the crude product, and filtration gave a colourless solution. The solvent was removed *in vacuo* to give a colourless oil, which was heated (150 °C) under vacuum (10^−3^ mbar) for 6 h. Hot toluene (50 mL) was used to extract the product, which was filtered. The solvent was removed under vacuum and the product dissolved in benzene (30 mL) and stored at 6 °C to afford colourless crystals. The supernatant was decanted, and the residual solvent was removed *in vacuo* to give 2-Y (0.865 g, 3.4 mmol). The supernatant was concentrated under vacuum to *ca.* 10 mL to yield an additional crop of 2-Y as colourless microcrystals (0.254 g, 1.0 mmol). Total yield = 1.119 g, 4.4 mmol, 73%. Anal. calcd for C_10_H_23_B_2_Y: C, 47.32; H, 9.13. Found: C, 47.00; H, 9.18. ^1^H NMR (400.13 MHz, C_6_D_6_, 298 K): *δ* = 2.06 (s, 15H, Cp-C(C*H*_3_)), 0.66 (br q, 8H, ^1^*J*_BH_ = 84.6 Hz, BH_4_). ^13^C{^1^H} NMR (125.79 MHz, C_6_D_6_, 298 K): *δ* = 123.5 (Cp-*C*(CH_3_)), 11.7 (Cp-C(*C*H_3_)). ^11^B{^1^H} NMR (160.48 MHz, C_6_D_6_, 298 K): *δ* = –22.0 (*B*H_4_). ^11^B NMR (128.38 MHz, C_6_D_6_, 298 K): *δ* = –22.0 (p, ^1^*J*_BH_ = 84.6 Hz, *B*H_4_). FTIR (ATR, microcrystalline): *ν̃* = 2970 (w, C–H stretch), 2941 (w, C–H stretch), 2910 (m, C–H stretch), 2859 (m, C–H stretch), 2499 (s, B–H_t_ stretch), 2277 (s, B–H_b_ stretch), 2203 (s, B–H_b_ stretch), 2133 (s, B–H_b_ stretch), 1485 (w), 1455 (w), 1432 (w), 1381 (w), 1204 (s), 1116 (s), 1093 (m), 1026 (m), 802 (w), 676 (w), 592 (w), 434 (s) cm^−1^.

### Preparation of [Dy(Cp*)(BH_4_)_2_]_∞_ (2-Dy)

Complex 2-Dy was prepared following analogous synthetic procedures to 2-Y from [Dy(BH_4_)_3_(THF)_3_] (2.117 g, 5.0 mmol) and KCp* (0.872 g, 5.0 mmol). The product was crystallised from toluene (10 mL) as bright yellow crystals after storage at −35 °C. The supernatant was decanted, and residual solvent removed *in vacuo* to afford 2-Dy (1.138 g, 3.2 mmol, 64%). A hexameric form that contains two lattice toluene molecules (2-Dy·0.33C_6_H_5_CH_3_) was also identified in this crop by single crystal XRD. Anal. calcd for C_10_H_23_B_2_Dy: C, 36.68; H, 7.08. Found: C, 35.45; H, 7.32. *μ*_eff_ product = 10.46 *μ*_B_ (Evans method, C_6_D_6_, 298 K). The paramagnetism of 2-Dy precluded the assignment of its ^1^H, ^13^C{^1^H} and ^11^B{^1^H} NMR spectra. FTIR (ATR, microcrystalline): *ν̃* = 2959 (w, C–H stretch), 2945 (w, C–H stretch), 2908 (m, C–H stretch), 2859 (m, C–H stretch), 2491 (s, B–H_t_ stretch), 2277 (s, B–H_b_ stretch), 2216 (m, B–H_b_ stretch), 2127 (s, B–H_b_ stretch), 1490 (w), 1451 (w), 1432 (w), 1379 (w), 1300 (w), 1262 (m), 1214 (m), 1196 (s), 1114 (m), 1097 (s), 1023 (s), 863 (w), 802 (m), 730 (m), 694 (w), 590 (w), 464 (w) cm^−1^.

### Preparation of [Dy{OC(CF_3_)_3_}_2_(FPh)_5_][{Al[OC(CF_3_)_3_]_3_}_2_(μ-F)] (3-Dy)

Fluorobenzene (5 mL) was added to a mixture of 2-Dy (0.107 g, 0.3 mmol) and [CPh_3_][Al{OC(CF_3_)_3_}_4_] (0.726 g, 0.6 mmol) at room temperature. The reaction mixture was heated to 70 °C for 30 minutes, the solvent was removed *in vacuo* and the resultant yellow oil washed with hexane (5 mL). The crude product was dissolved in fluorobenzene (1 mL) and layered with hexane (5 mL) to give several crystals of 3-Dy.

### Preparation of [Y(Cp′′′)(Cp*)(FPh)_2_][{Al[OC(CF_3_)_3_]_3_}_2_(μ-F)] (4-Y)

Fluorobenzene (10 mL) was added to a mixture of 1-Y (0.948 g, 0.25 mmol) and KCp′′′ (0.080 g, 0.25 mmol). The yellow reaction mixture was stirred at room temperature for 7 days. The solution was concentrated *in vacuo* to *ca.* 5 mL; *n*-hexane (5 mL) was then added, and the mixture was vigorously stirred to form a light yellow solution and an off-white solid. The supernatant was separated by filtration and concentrated *in vacuo* to *ca.* 4 mL; the mixture was stored at −35 °C for 3 days to afford light yellow crystals. The supernatant was decanted, and the residual solvent was removed under vacuum to give 4-Y (0.312 g, 0.17 mmol, 57%). Anal. calcd for C_60_H_54_Al_2_F_57_O_6_Si_3_Y: C, 33.04; H, 2.50. Found: C, 31.46; H, 2.21. ^1^H NMR (400.13 MHz, C_6_H_5_F with a C_6_D_6_/C_6_H_5_F insert, 298 K): *δ* = 7.45 (s, 2H, Cp-C*H*), 1.90 (s, 15H, Cp-C*H*_3_), 0.30 (s, 9H, Si(C*H*_3_)_3_), 0.26 (s, 18H, Si(C*H*_3_)_3_). ^13^C{^1^H} NMR (125.79 MHz, C_6_H_5_F with a C_6_D_6_/C_6_H_5_F insert, 298 K): *δ* = 141.1 (Cp-*C*Si(CH_3_)_3_), 140.3 (Cp-*C*H), 134.6 (Cp-*C*Si(CH_3_)_3_), 125.8 (Cp-*C*(CH_3_)), 11.5 (Cp-C(*C*H_3_)) 1.0 (Si(*C*H_3_)_3_), 0.3 (Si(*C*H_3_)_3_). ^19^F NMR (376.46 MHz, C_6_H_5_F with a C_6_D_6_/C_6_H_5_F insert, 298 K): *δ* = −75.4 (s, [{Al[OC(C*F*_3_)_3_]_3_}_2_(μ-F)]), −113.7 (br. s, C_6_H_5_*F*), −184.5 (s, [{Al[OC(CF_3_)_3_]_3_}_2_(μ-*F*)]). ^29^Si DEPT90 NMR (80 MHz, C_6_H_5_F with a C_6_D_6_/C_6_H_5_F insert, 298 K): *δ* = −8.37 (*Si*(CH_3_)_3_), −8.64 (*Si*(CH_3_)_3_). FTIR (ATR, microcrystalline): *ν̃* = 2961 (w, C–H stretch), 2923 (w, C–H stretch), 1580 (w), 1484 (m), 1354 (m, C–O stretch), 1301 (s), 1276 (s, C–F stretch), 1241 (s), 1215 (s), 1169 (s), 1114 (m), 1087 (m), 1069 (w), 1019 (w), 972 (s), 935 (m), 896 (w), 859 (m), 834 (s), 773 (m), 752 (s), 725 (s), 705 (w), 676 (w), 633 (s), 569 (m), 536 (s), 507 (w), 483 (w), 450 (s), 425 (m) cm^−1^.

### [CPh_3_][{Al[OC(CF_3_)_3_]_3_}_2_(μ-F)]

Prepared by a modification of the published procedure.^49^*Ortho*-difluorobenzene (50 mL) was added to a mixture of [CPh_3_][PF_6_] (3.657 g, 9.4 mmol) and PhF-Al{OC(CF_3_)_3_}_3_ (15.604 g, 18.8 mmol); an immediate effervescence and formation of a dark yellow solution was observed. The reaction mixture was stirred at room temperature for 16 h. The solvent was removed *in vacuo*, and the remaining solid washed with pentane (3 × 30 mL). The residual solvent was removed under vacuum and the product was obtained as a bright yellow powder (15.537 g, 9.0 mmol, 96%). The identity of the product was confirmed by comparison to literature NMR spectroscopic data.

## Data availability

Correspondence and requests for materials should be directed to N. F. C. and D. P. M. Crystallographic data for the structures reported in this article have been deposited at the Cambridge Crystallographic Data Centre, under deposition numbers CCDC 2365779 (1-Y), 2365780 (1-Dy), 2365781 (2-Y), 2365782 (2-Dy), 2365783 (2-Dy·0.33C_6_H_5_CH_3_), 2365784 (3-Dy), 2365785 (4-Y). Raw research data files supporting this publication are available from Figshare at https://figshare.com/articles/dataset/_b_A_six-legged_piano_stool_dysprosium_single-molecule_magnet_b_/26105386?file=47251777. Apart from the data sets mentioned, all other data supporting the findings of this study are available within the article and ESI.†

## Author contributions

S. C. C. and D. P. M. provided the original concept. S. C. C. synthesised and characterised the complexes. S. C. C. collected and finalised the SCXRD data. S. C. C. collected the PXRD data sets; G. F. S. W. solved and refined the PXRD datasets. S. C. C. performed DFT and *ab initio* calculations. S. C. C. and G. K. G. collected the magnetic data. S. C. C., W. J. A. B. and G. K. G. interpreted the magnetic data. A. M. designed the dipolar field model, wrote the code and performed the calculations. N. F. C. supervised the magnetism and computational components. D. P. M. supervised the synthetic component and directed the research. S. C. C., W. J. A. B., G. K. G., N. F. C. and D. P. M. wrote the manuscript, with contributions from all authors.

## Conflicts of interest

The authors declare no competing interests.

## Supplementary Material

SC-OLF-D4SC06661H-s001

SC-OLF-D4SC06661H-s002
