# Supplementary material for: A fluorobenzene-bound dysprosium half-sandwich dication single-molecule magnet
Source: Chem Sci. 2024 Dec 4;16(2):610–20. doi: 10.1039/d4sc06661h (PMC11616726; doi:10.1039/d4sc06661h)
Supplement: SC-016-D4SC06661H-s001 [file SC-016-D4SC06661H-s001.pdf]

Supplementary Information (SI) for Chemical Science.
This journal is © The Royal Society of Chemistry 2024

Supporting Information for

A fluorobenzene-bound dysprosium half-sandwich dication single-molecule magnet

Sophie C. Corner,[a] William J. A. Blackmore,[a] Gemma K. Gransbury,[a] Andrea Mattioni,[a] George F. S. Whitehead,[a] Nicholas F. Chilton[a,b] and David P. Mills[a]**

[a]Department of Chemistry, The University of Manchester, Oxford Road, Manchester, M13 9PL, U.K.

[b]Research School of Chemistry, The Australian National University, Building 137, Sullivans Creek Road, Canberra, ACT, 2601, Australia

*Email: nicholas.chilton@anu.edu.au, david.mills@manchester.ac.uk.

Contents

1. **Infrared spectroscopy** S2
2. **DFT calculations** S5
3. **Powder X-ray diffraction** S8
4. **NMR spectroscopy** S11
5. **Single crystal X-ray diffraction** S17
6. **Magnetic studies** S32
7. **CASSCF-SO calculations** S45
8. **Dipolar calculations** S55
9. **References** S57

1. Infrared spectroscopy

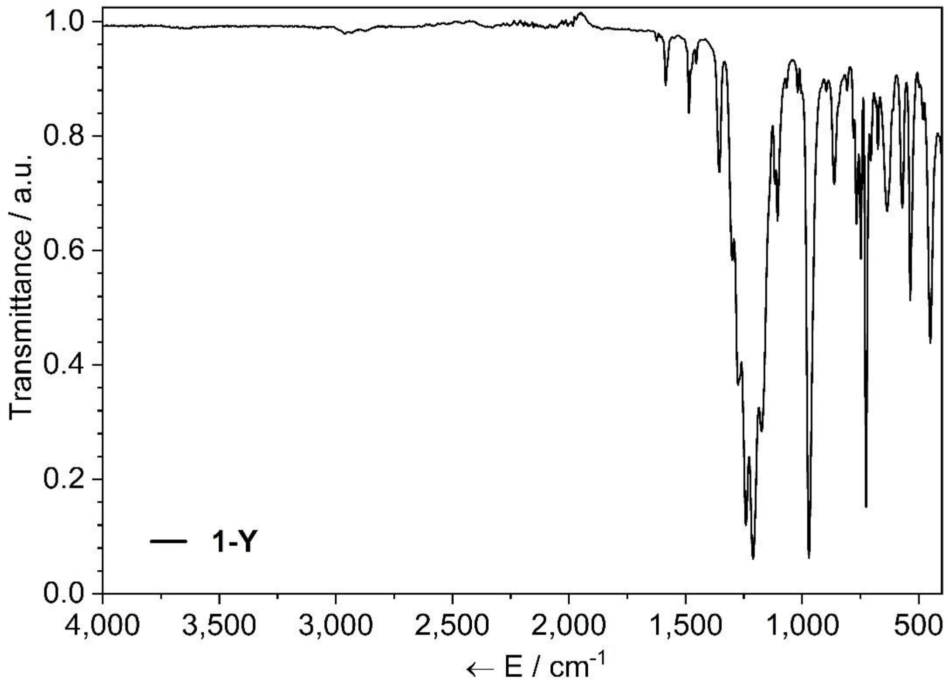

Figure S1. ATR-IR spectrum of **1-Y**, recorded as a microcrystalline powder.

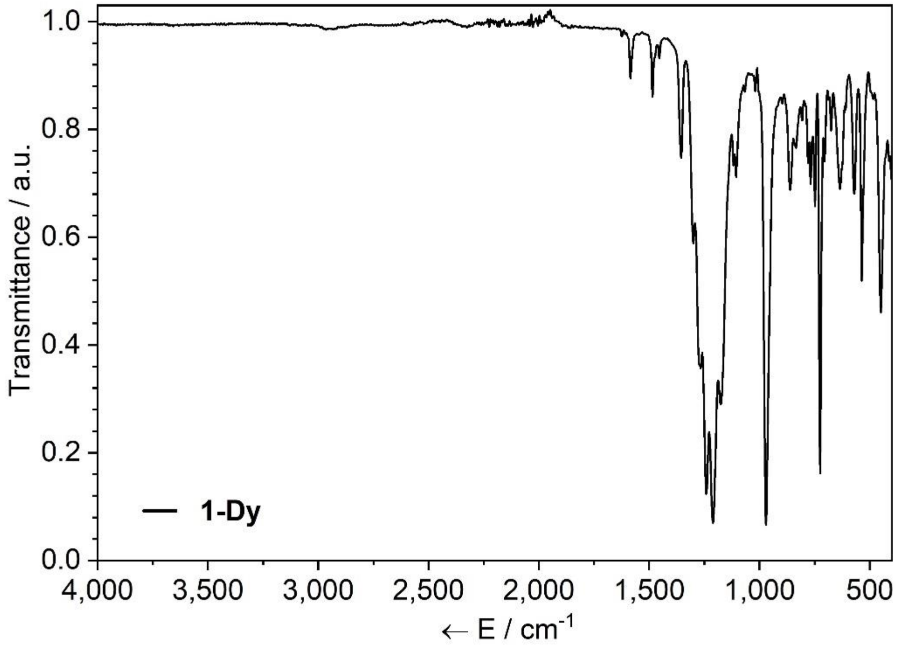

Figure S2. ATR-IR spectrum of **1-Dy**, recorded as a microcrystalline powder.

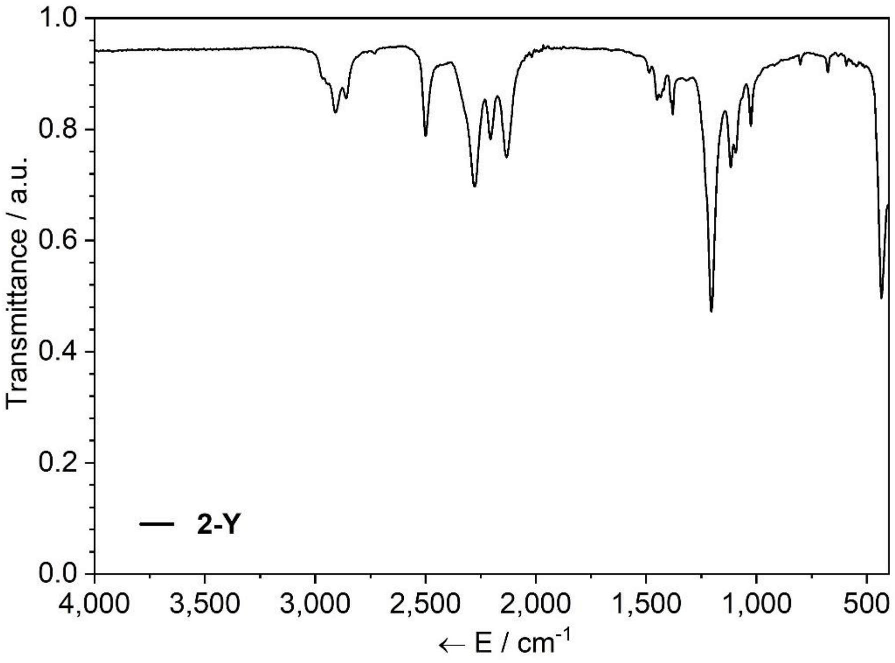

Figure S3. ATR-IR spectrum of **2-Y**, recorded as a microcrystalline powder.

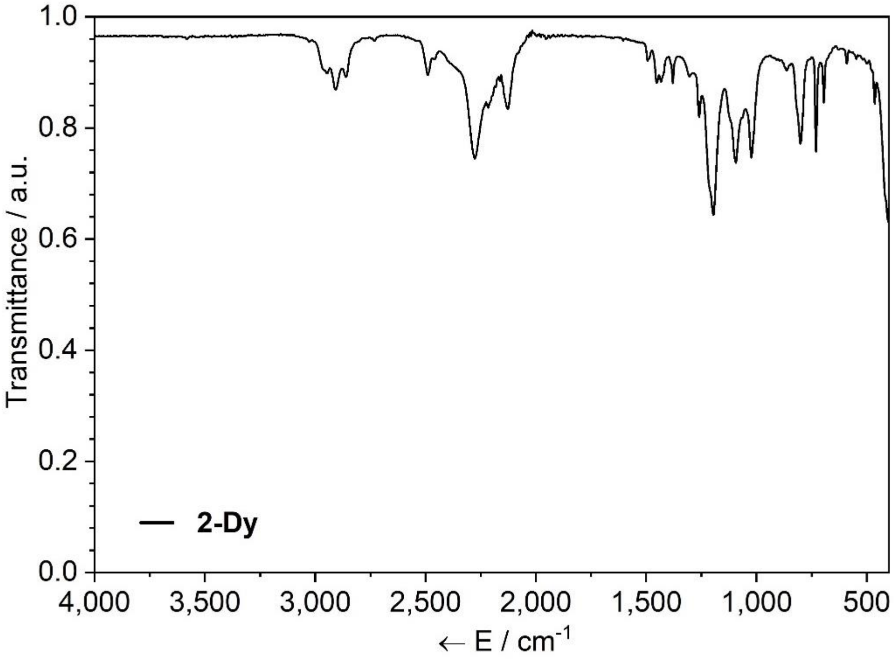

Figure S4. ATR-IR spectrum of **2-Dy**, recorded as a microcrystalline powder.

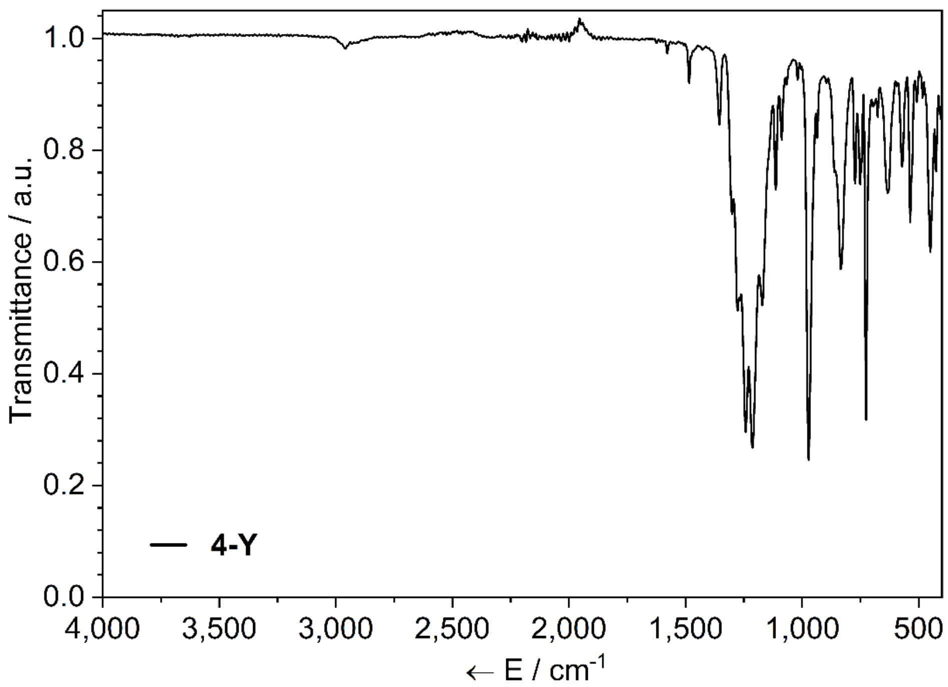

Figure S5. ATR-IR spectrum of **4-Y**, recorded as a microcrystalline powder.

2. DFT calculations

DFT calculations were performed using the Orca 5.0.3 software package.[1–3] The PBE0 hybrid functional, augmented by Stefan Grimme's D4 dispersion correction,[4,5] the Def2-TZVP basis set,[6–8] and the RIJCOSX approximation were employed throughout. The initial coordinates were obtained through the unoptimized XRD data of **1-Y**. Atom coordinates were verified as minima on the potential energy surface by the absence of imaginary vibrational modes.

Table S1. Calculated atomic coordinates for $[Y(Cp^*)(FPh)_6]^{2+}$.

Atom	X	Y	Z	Atom	x	y	z
39	16.21419	15.89801	22.66978	1	13.17375	9.44035	23.29003
9	16.15039	14.72383	24.78344	6	14.87675	10.63853	23.79714
9	16.04256	13.58358	22.02707	1	15.14064	10.04367	24.66307
9	18.44051	14.82834	22.87786	6	15.65770	11.73812	23.46301
9	17.56509	17.23137	24.08963	1	16.52533	12.01274	24.04631
9	14.83636	17.24533	24.03976	6	19.20613	13.98482	23.67103
9	13.85618	15.15539	22.56437	6	19.19910	12.64564	23.36309
6	15.25322	17.42079	20.87574	1	18.64299	12.27442	22.51202
6	15.71721	16.25385	20.21335	6	19.94913	11.80275	24.17441
6	17.13189	16.21305	20.33568	1	19.97401	10.74163	23.95908
6	17.54072	17.36010	21.06730	6	20.67195	12.31592	25.24256
6	16.37945	18.10392	21.40291	1	21.25881	11.65232	25.86540
6	13.84872	17.92311	20.90863	6	20.66219	13.67894	25.50136
1	13.56034	18.30017	21.89293	1	21.23743	14.08286	26.32546
1	13.13899	17.15069	20.61397	6	19.91814	14.54079	24.70607
1	13.72799	18.75328	20.20680	1	19.90002	15.60556	24.89422
6	14.88129	15.30929	19.41767	6	18.19790	17.53765	25.29789
1	13.89091	15.16488	19.85338	6	19.39413	18.20879	25.22354
1	15.35905	14.33446	19.31428	1	19.82041	18.48752	24.26905
1	14.73088	15.69904	18.40677	6	20.03015	18.50753	26.42273
6	18.04211	15.20680	19.71380	1	20.97356	19.03897	26.40436
1	17.61611	14.20157	19.71686	6	19.46294	18.13324	27.63230
1	19.00755	15.16751	20.21893	1	19.96544	18.37155	28.56153
1	18.23658	15.46715	18.66931	6	18.24855	17.46410	27.65434
6	18.95107	17.77012	21.33106	1	17.79792	17.17855	28.59710
1	19.57212	16.94685	21.69081	6	17.59203	17.15752	26.46988
1	18.99833	18.57917	22.05943	1	16.63786	16.65274	26.47992
1	19.41499	18.14071	20.41290	6	14.31045	17.90583	25.15874
6	16.33707	19.43341	22.07978	6	14.97981	19.00970	25.62568
1	17.15507	19.56909	22.78920	1	15.87406	19.37267	25.13873
1	15.39578	19.59069	22.60846	6	14.45733	19.63728	26.74978
1	16.42178	20.23669	21.34189	1	14.95783	20.51084	27.14883
6	16.06382	14.14729	26.05797	6	13.30564	19.15570	27.35437
6	17.06540	13.29125	26.44233	1	12.90623	19.65374	28.22911
1	17.90067	13.07187	25.79170	6	12.65733	18.04612	26.83339
6	16.96752	12.73522	27.71144	1	11.74868	17.67798	27.29417
1	17.73960	12.05567	28.05091	6	13.15893	17.40007	25.71051
6	15.89907	13.04803	28.53888	1	12.65622	16.54555	25.27871
1	15.83391	12.60897	29.52670	6	12.71181	14.88300	23.30492
6	14.91609	13.92451	28.10455	6	12.77649	13.89534	24.25748
1	14.08290	14.17342	28.75046	1	13.67761	13.31612	24.40679
6	14.98881	14.49246	26.83864	6	11.63037	13.65638	25.00480
1	14.23396	15.18026	26.48423	1	11.64387	12.88154	25.76172
6	15.28435	12.45851	22.35502	6	10.47653	14.39194	24.77672
6	14.21146	12.15869	21.55367	1	9.58585	14.19496	25.36040
1	13.97658	12.76252	20.68764	6	10.45557	15.36969	23.79201
6	13.44772	11.05446	21.90722	1	9.55250	15.93819	23.60740
1	12.59348	10.78474	21.29888	6	11.59072	15.62983	23.03475
6	13.77493	10.30117	23.02557	1	11.60091	16.39314	22.26848

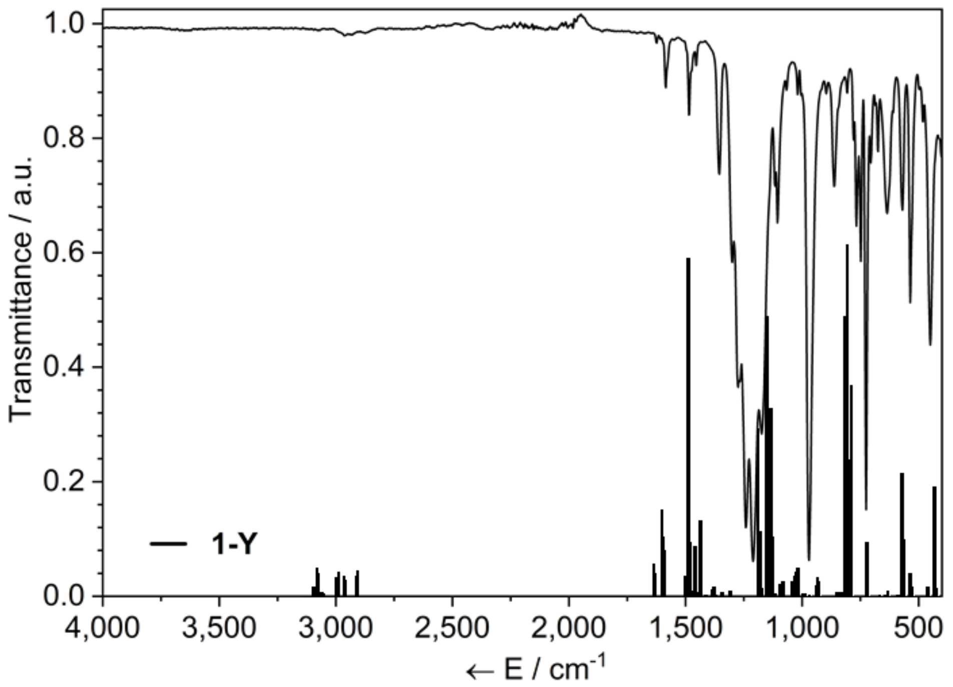

Figure S6. Overlay of the simulated IR spectrum of $[Y(Cp^*)(FPh)_6]^{2+}$ and the measured ATR-IR spectrum of **1-Y**.

3. Powder X-ray diffraction

Data collection. Powder X-ray diffraction (PXRD) data of microcrystalline samples of **1-Dy,** mounted with a minimum amount of Fomblin® onto a 0.1 mm glass fibre, were collected at 100 K using a Rigaku FR-X rotating anode single crystal X-ray diffractometer using Cu Kα radiation (λ = 1.5418 Å) with a Hypix-6000HE detector and an Oxford Cryosystems nitrogen flow gas system. Data were collected between 3–70 °θ, with a detector distance of 150 mm and a beam divergence of 1.0 mRad.[9] X-ray data were collected using CrysAlisPro software.[10]

Data processing. The instrument was calibrated using the collected data, with the instrument model refined using diffraction peak positions measured at multiple detector angles. The data were reduced and integrated using CrysAlisPro software.[10] Le Bail profile analysis was performed using JANA2020 software.[11] Initial unit call parameters were taken from the corresponding single crystal data.

Data analysis. The data are presented in Figures S7 and S8 and Table S2. The peaks of the diffractograms are generally in excellent agreement with the peak positions calculated, providing confidence that **1-Dy** show a high phase purity. The Le Bail refinement indicates a contraction of the unit cell; the difference in relative peak position increases with angle, indicating a significant unit cell difference rather than an artefact of the data processing. The unit cell volume reduction is 485 $Å^3$, or approximately the loss of 6 non-hydrogen atoms per asymmetric unit. This indicates that the lattice hexane is lost upon drying or grinding the crystal, which is in accord with elemental analysis data.

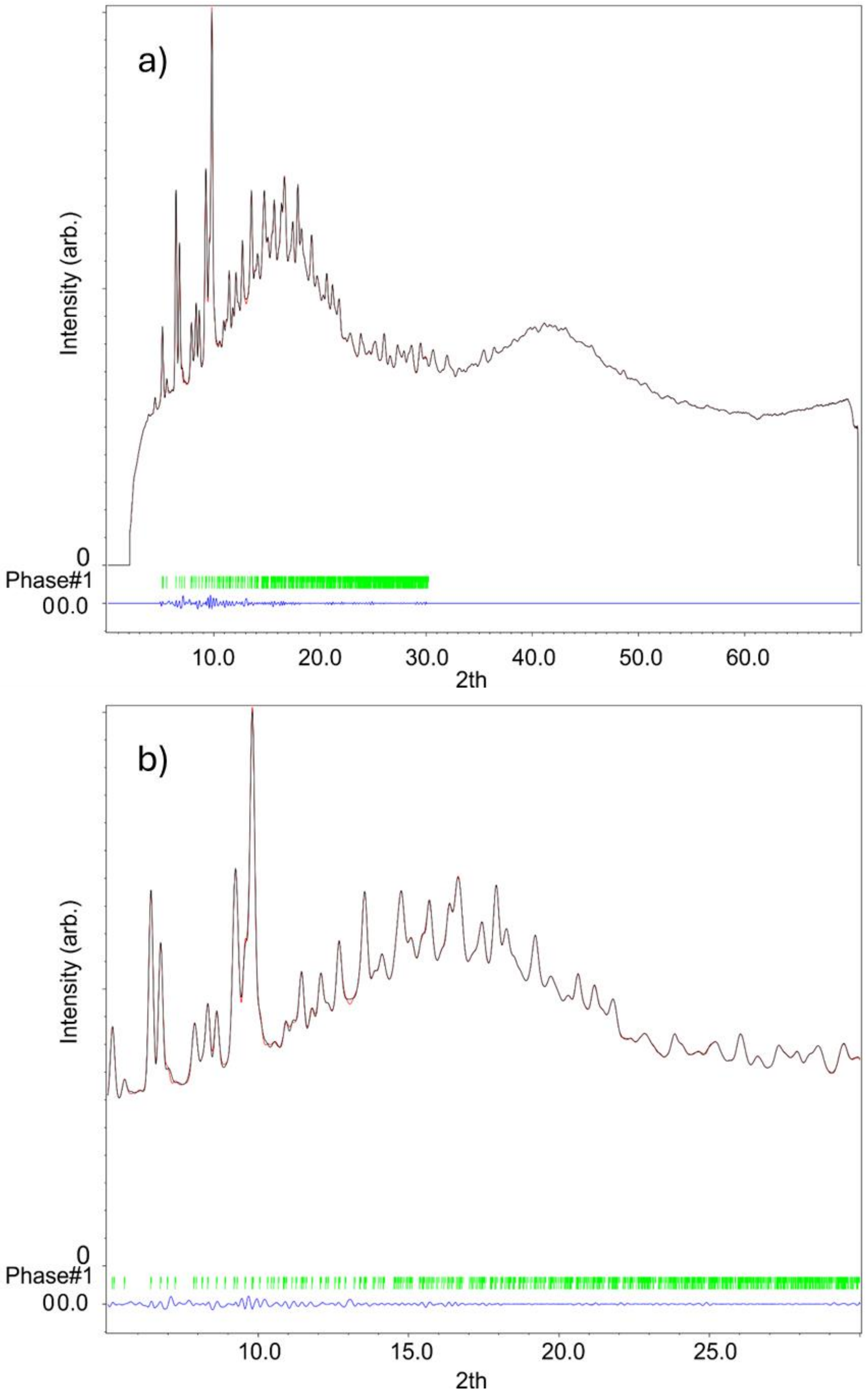

Figure S7. (a) Full PXRD pattern for **1-Dy** at 100 K. (b) Selected range for Le Bail profile fitting. Experimental powder X-ray diffraction pattern (black), expected from SCXRD data (red), difference (blue) and reflection positions (green).

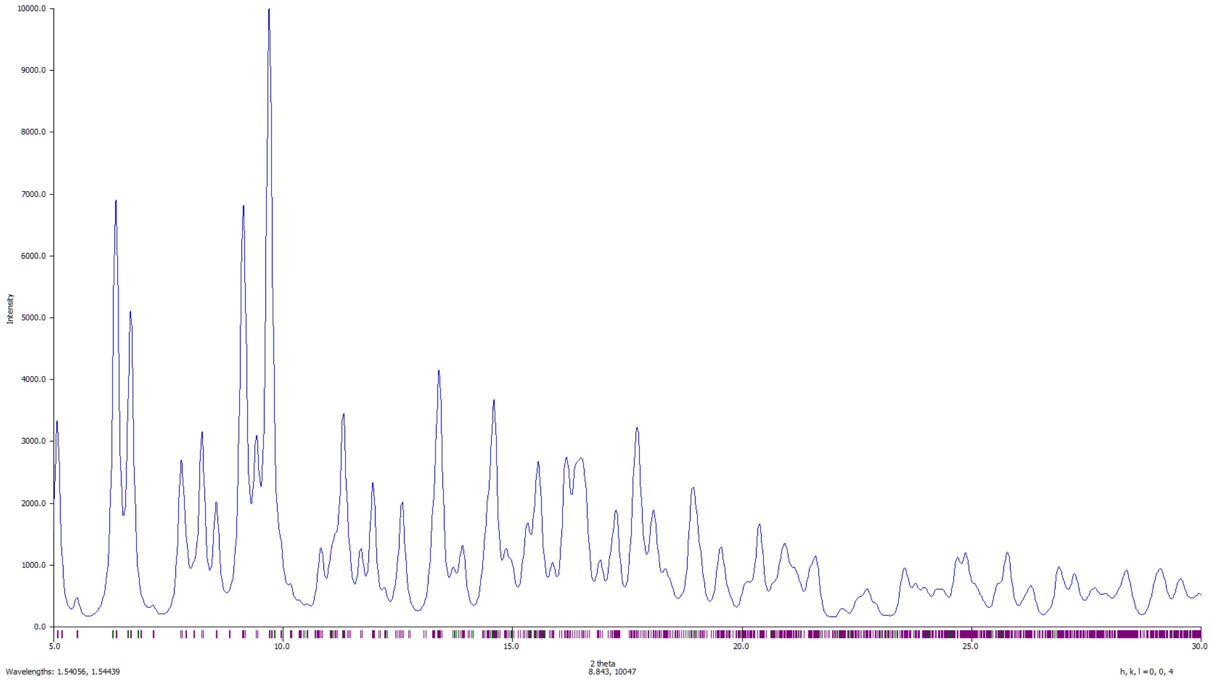

Figure S8. Calculated PXRD pattern for **1-Dy** from SCXRD data (FWHM 0.15).[12]

Table S2. Unit cell values obtained from Le Bail refinement results for **1-Dy** with those expected from SCXRD data.

**Dataset**	***a***	***b***	***c***	***α***	***β***	***γ***
**PXRD**	18.0557(7)	18.9876(8)	39.812(2)	90	94.866(2)	90
**SCXRD**	18.2139(3)	19.3140(4)	40.1803(6)	90	94.8490(10)	90

4. NMR spectroscopy

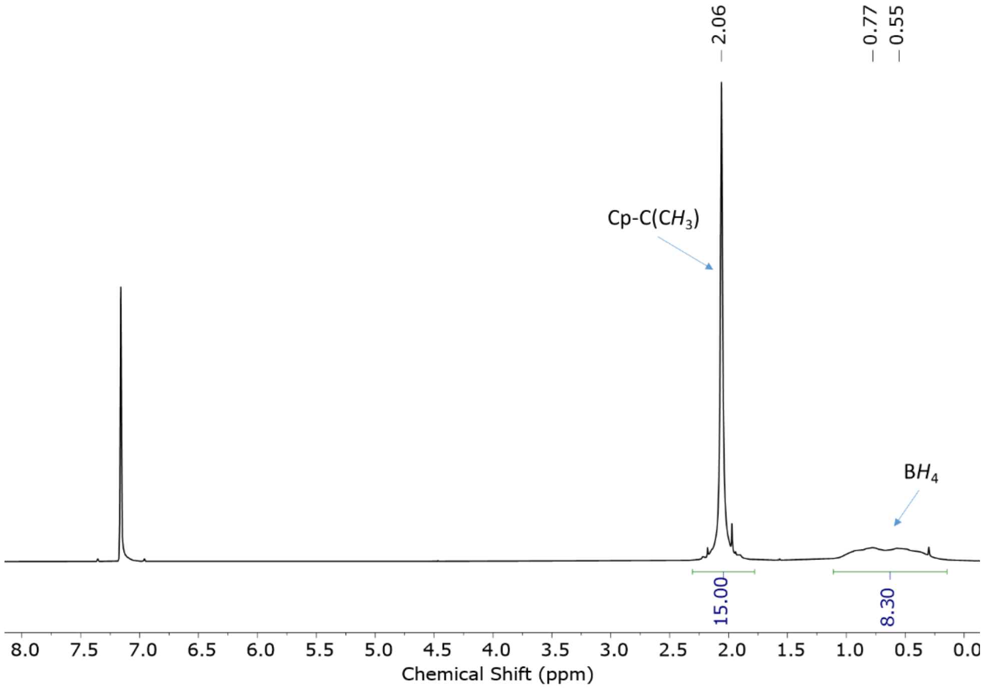

Figure S9. ^{1}H NMR spectrum of **2-Y** (400 MHz) in C_6D_6.

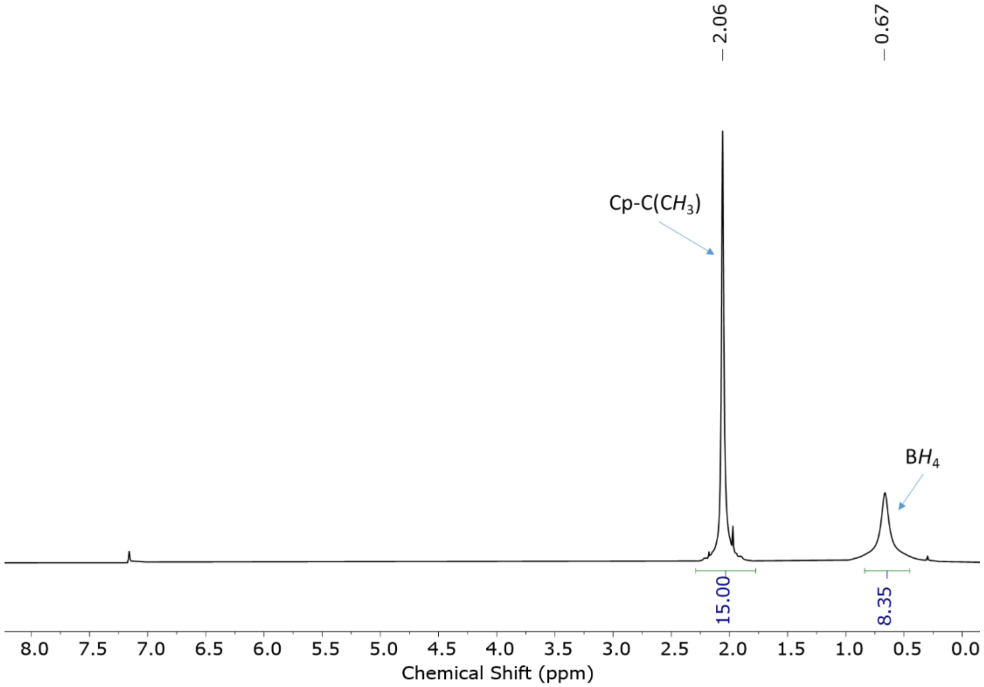

Figure S10. ^{1}H{^{11}B} NMR spectrum of **2-Y** (400 MHz) in C_6D_6.

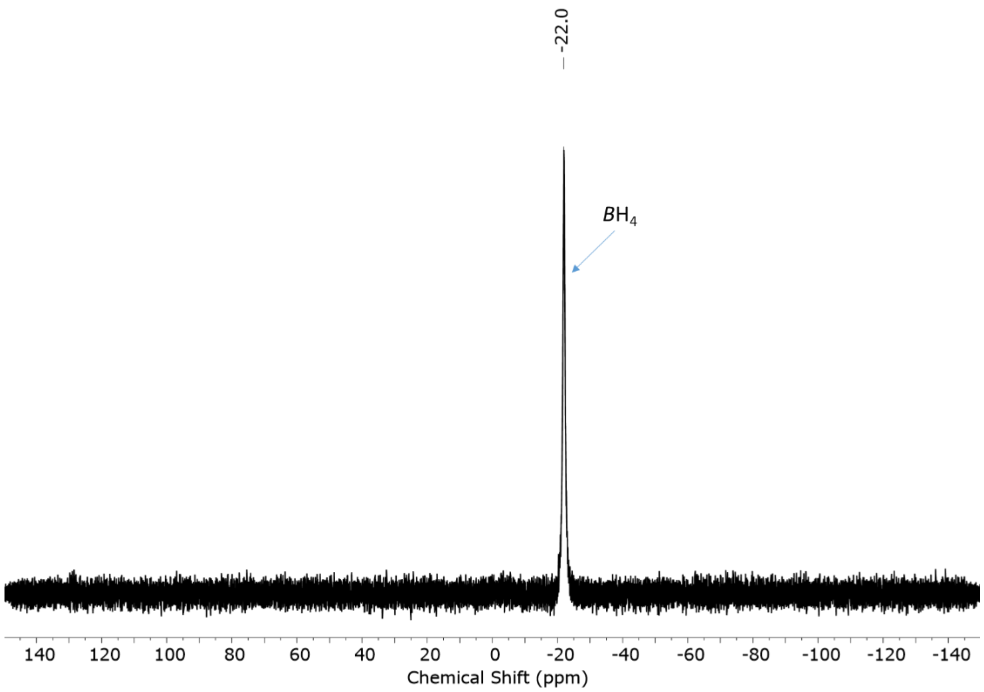

Figure S11. $^{11}B\{^{1}H\}$ NMR spectrum of **2-Y** (160 MHz) in C_6D_6.

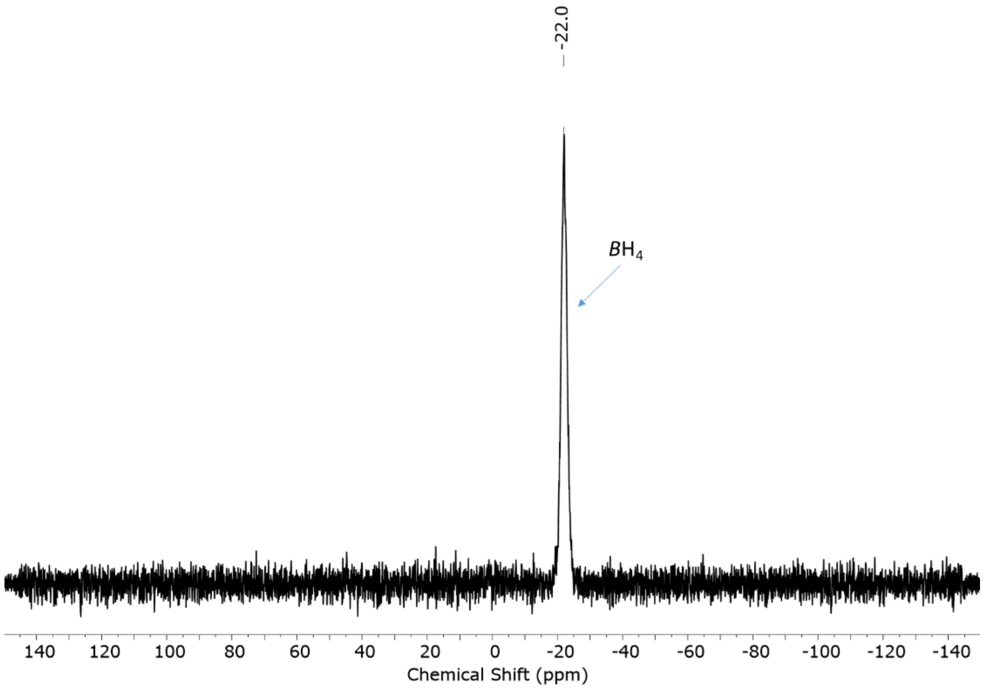

Figure S12. ^{11}B NMR spectrum of **2-Y** (160 MHz) in C_6D_6.

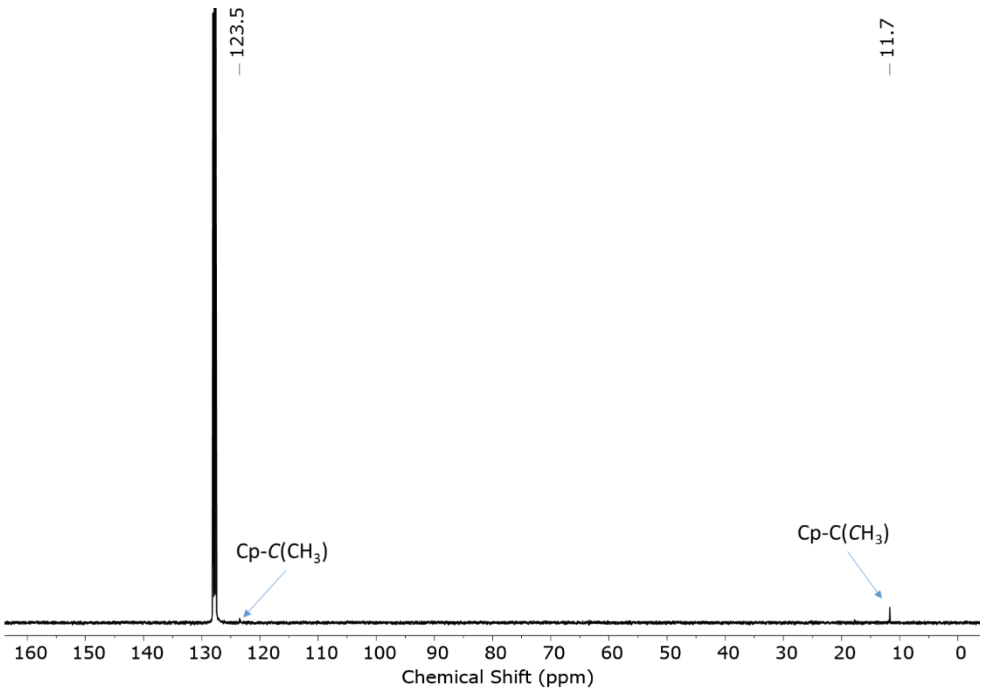

Figure S13. $^{13}C\{^{1}H\}$ NMR spectrum of **2-Y** (126 MHz) in C_6D_6.

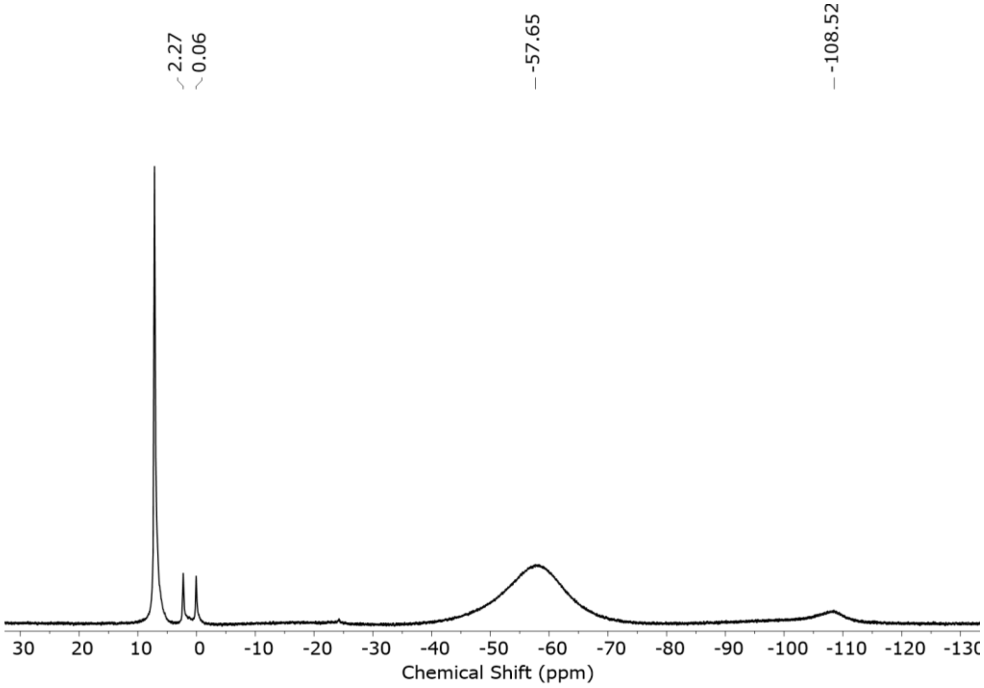

Figure S14. ^{1}H NMR spectrum of **2-Dy** (400 MHz) in C_6D_6; full spectral range 200 to –200 ppm.

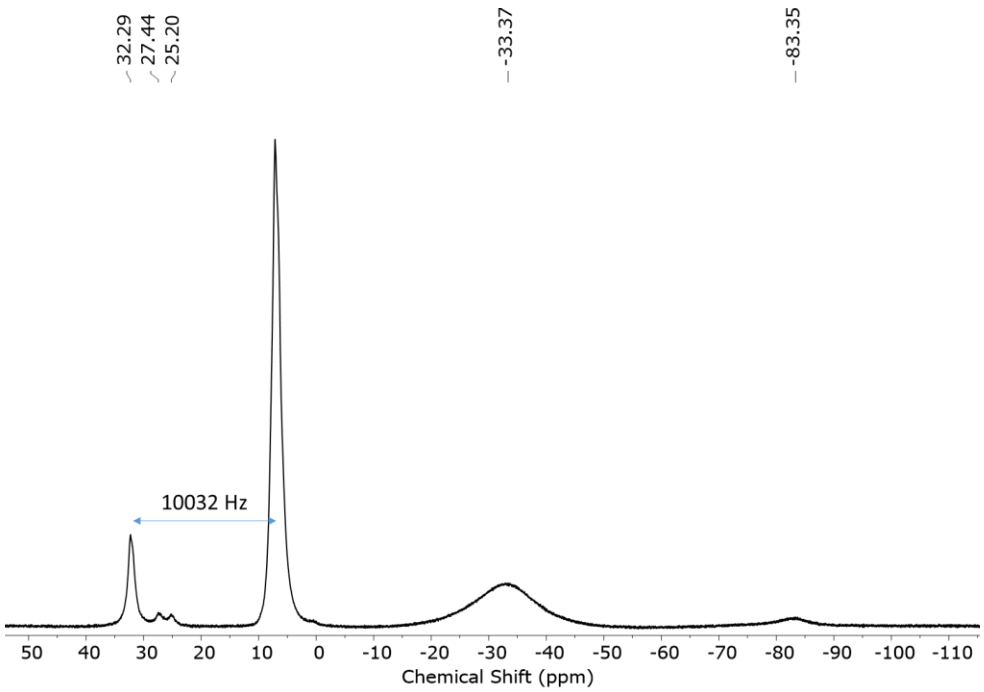

Figure S15. ^{1}H NMR spectrum of **2-Dy** (400 MHz) in C_6D_6 with a C_6D_6 insert; full spectral range 200 to –200 ppm.

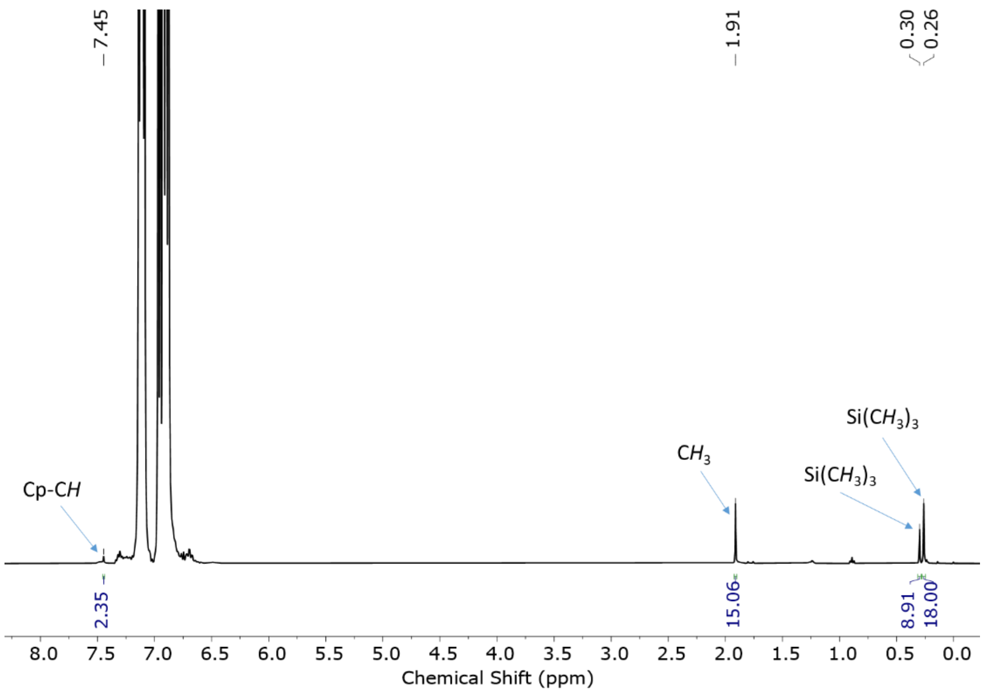

Figure S16. ^{1}H NMR spectrum of **4-Y** (400 MHz) in C_6H_5F with a C_6D_6/C_6H_5F insert.

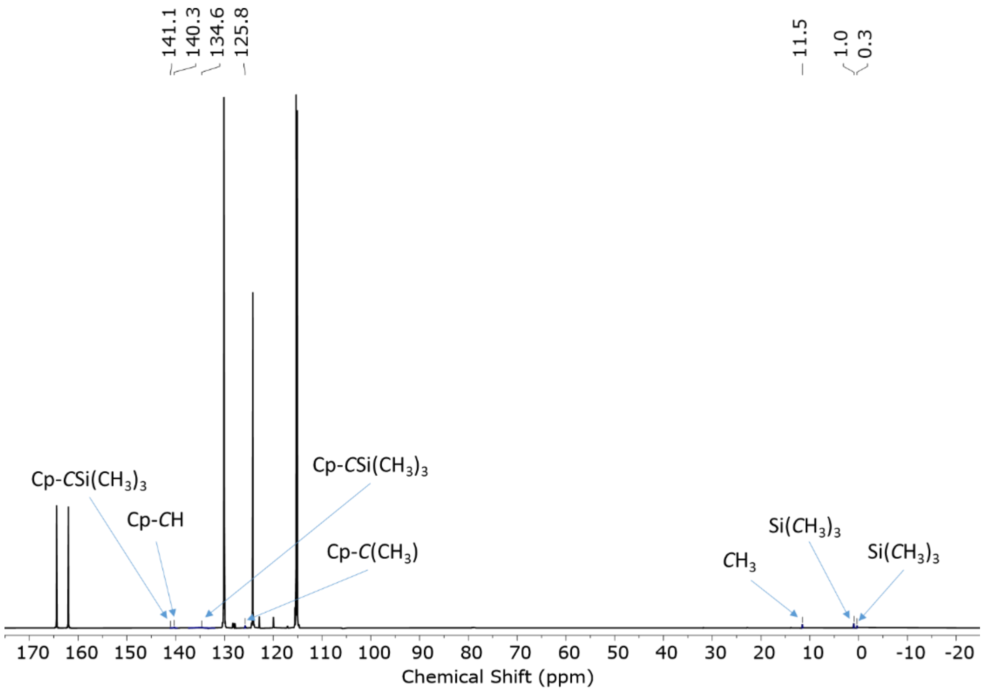

Figure S17. $^{13}C\{^{1}H\}$ NMR spectrum of **4-Y** (126 MHz) in C_6H_5F with a C_6D_6/C_6H_5F insert.

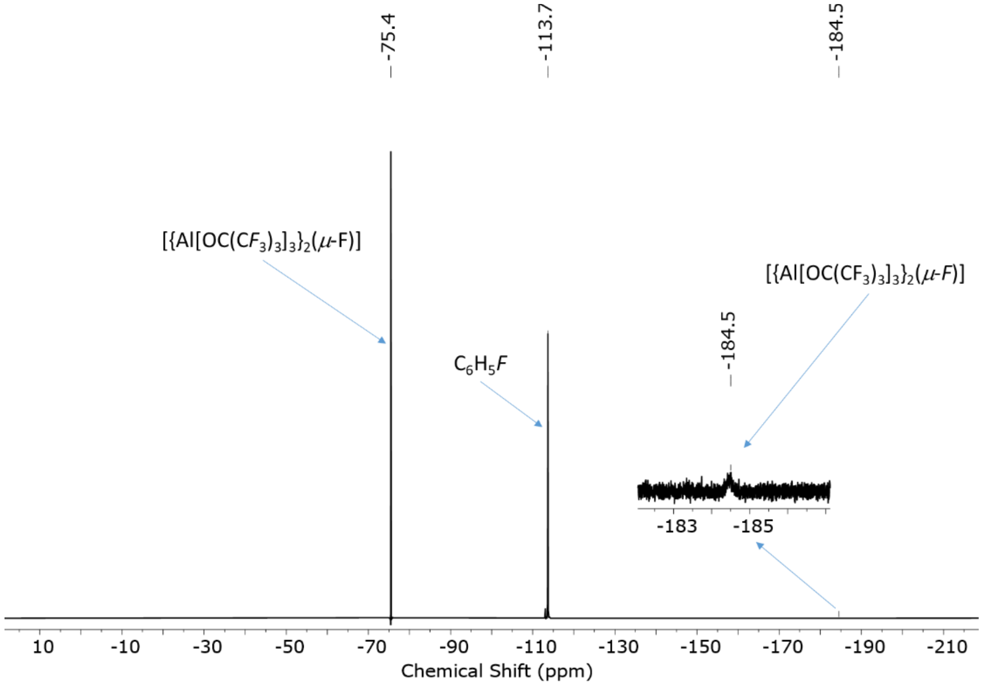

Figure S18. ^{19}F NMR spectrum of **4-Y** (376 MHz) in C_6H_5F with a C_6D_6/C_6H_5F insert.

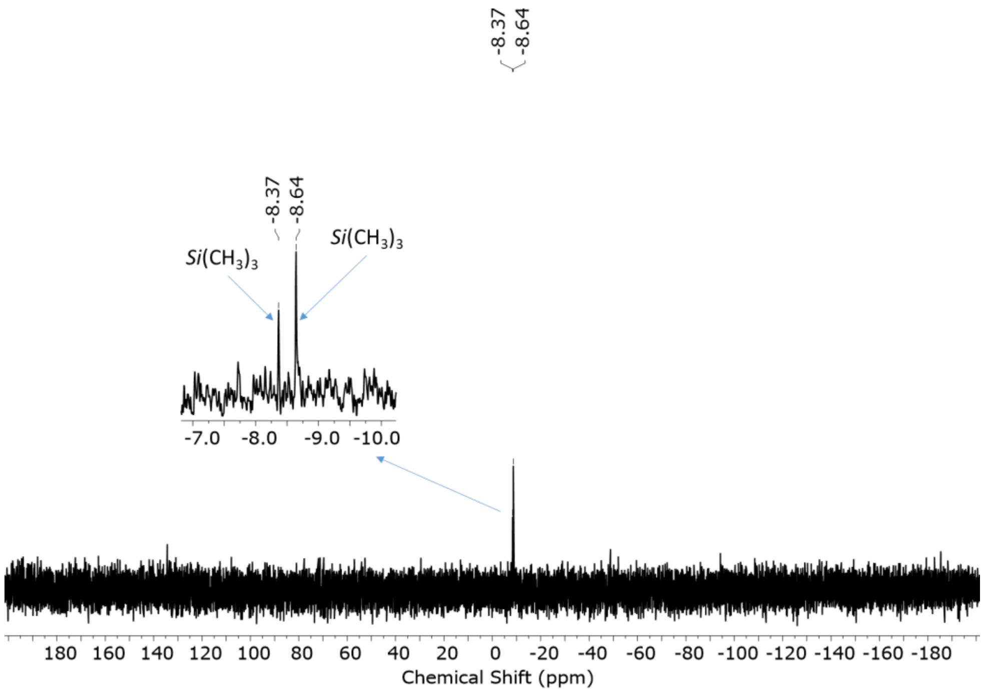

Figure S19. ^{29}Si NMR spectrum of **4-Y** (80 MHz) in C_6H_5F with a C_6D_6/C_6H_5F insert.

5. Single crystal X-ray diffraction

Single crystal X-ray diffraction (SCXRD) data were collected using a dual wavelength Rigaku FR-X rotating anode diffractometer using CuKα (λ = 1.54146 Å) radiation, equipped with an AFC-11 4-circle goniometer, VariMAX^{TM} microfocus optics and a Hypix-6000HE detector (**1-Dy 2-Y**, **2-Dy·0.33C$_6$H$_5$CH$_3$**, **3-Dy** and **4-Y**) or a Rigaku XtalLAB Synergy-S diffractometer equipped with a HyPix 6000HE photon counting pixel array detector with a mirror-monochromated Mo Kα X-ray source (**1-Y** and **2-Dy**). A temperature of 100(2) K was set by using an Oxford Cryosystems 800 plus nitrogen flow gas system. Data were collected and reduced using CrysAlisPro v42.[10] Absorption correction was performed using empirical methods (SCALE3 ABSPACK) based upon symmetry-equivalent reflections combined with measurements at different azimuthal angles. The structures for were solved and refined against all F^2 values using Shelx-2018/3 implemented through Olex2 v1.5.[13,14] ORTEP-3[15] and POV-Ray[16] were employed for molecular graphics.

Selected bond lengths and angles for **1-Dy**: Dy(1)···Cp*$_{centroid}$: 2.275(3) Å; Dy(1)–F(1): 2.393(4) Å; Dy(1)–F(2): 2.427(3) Å; Dy(1)–F(3): 2.388(4) Å; Dy(1)–F(4): 2.363(5) Å; Dy(1)–F(5): 2.433(4) Å; Dy(1)–F(6): 2.411(3); C(11)–F(1): 1.404(6) Å; C(17)–F(2): 1.410(7) Å; C(23)–F(3): 1.422(7) Å; C(29)–F(4): 1.367(7) Å; C(35)–F(5): 1.415(7) Å, C(41)–F(6): 1.438(7) Å; Cp*$_{centroid}$···Dy(1)–F(1): 176.28(11)°; Cp*$_{centroid}$···Dy(1)–F(2): 101.65(10)°; Cp*$_{centroid}$···Dy(1)–F(3): 103.22(12)°; Cp*$_{centroid}$···Dy(1)–F(4): 106.54(14)°; Cp*$_{centroid}$···Dy(1)–F(5): 103.32(11)°; Cp*$_{centroid}$···Dy(1)–F(6): 103.71(10)°; F(1)–Dy(1)–F(2): 74.86(12)°; F(1)–Dy(1)–F(3): 76.96(14)°; F(1)–Dy(1)–F(4): 77.1(2)°; F(1)–Dy(1)–F(5): 78.67(13)°; F(1)–Dy(1)–F(6): 73.94(12)°; F(2)–Dy(1)–F(3): 70.47(12)°; F(3)–Dy(1)–F(4): 70.8(2)°; F(4)–Dy(1)–F(5): 69.3(2)°; F(5)–Dy(1)–F(6): 69.35(12)°; F(6)–Dy(1)–F(2): 68.43(12)°; Dy(1)–F(1)–C(11): 149.2(3)°; Dy(1)–F(2)–C(17): 139.3(3)°; Dy(1)–F(3)–C(23):.166.6(4)°; Dy(1)–F(4)–C(29): 166.3(4)°; Dy(1)–F(5)–C(35): 154.5(3)°; Dy(1)–F(6)–C(41): 146.0(3)°.

Selected bond lengths and angles for **4-Y**: Y(1)···Cp‴$_{centroid}$: 2.3100(4) Å; Y(1)···Cp*$_{centroid}$: 2.3703(4) Å; Y(1)–F(1): 2.367(12) Å; Y(1)–F(2): 2.370(13) Å; F(1)–C(25): 1.38(2) Å; F(2)–C(31): 1.40(2) Å; Cp‴$_{centroid}$···Y(1)···Cp*$_{centroid}$: 138.49(2)°; Cp‴$_{centroid}$···Y(1)–F(1): 107.45(5)°; Cp‴$_{centroid}$···Y(1)–F(2): 108.0(6)°; Cp*$_{centroid}$···Y(1)–F(1): 102.0(6)°; Cp*$_{centroid}$···Y(1)–F(2): 105.4(7)°; F(1)–Y(1)–F(2): 79.3(6)°; Y(1)–F(1)···C(25): 161.3(2)°; Y(1)–F(2)···C(31): 162.1(2)°.

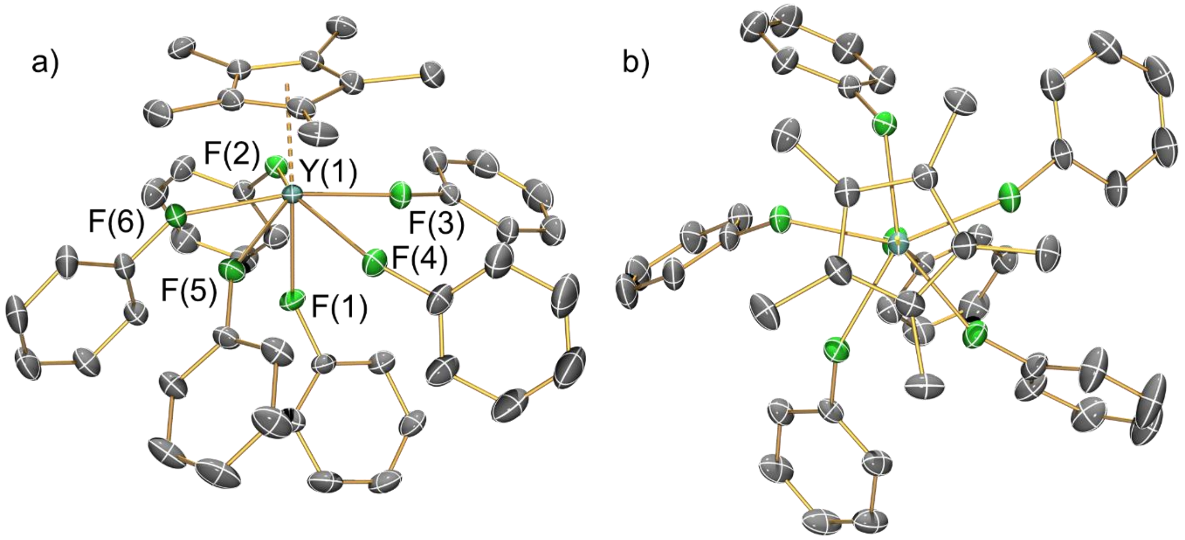

Figure S20. SCXRD structure of the dication of **1-Y** with selected atom labelling (Y: cyan, C: grey, F: green). Displacement ellipsoids set at 30% probability levels; the hydrogen atoms, lattice solvents and counteranions have been omitted for clarity. Selected bond lengths and angles: Y(1)···Cp*$_{centroid}$: 2.2704(4) Å; Y(1)–F(1): 2.382(3) Å; Y(1)–F(2): 2.385(3) Å; Y(1)–F(3): 2.376(3) Å; Y(1)–F(4): 2.357(3) Å; Y(1)–F(5): 2.400(3) Å; Y(1)–F(6): 2.393(2); C(11)–F(1): 1.423(5) Å; C(17)–F(2): 1.407(5) Å; C(23)–F(3): 1.425(5) Å; C(29)–F(4): 1.410(5) Å; C(35)–F(5): 1.417(5) Å, C(41)–F(6): 1.413(5) Å; Cp*$_{centroid}$···Y(1)–F(1): 175.46(7)°; Cp*$_{centroid}$···Y(1)–F(2): 101.27(7)°; Cp*$_{centroid}$···Y(1)–F(3): 103.96(8)°; Cp*$_{centroid}$···Y(1)–F(4): 107.31(9)°; Cp*$_{centroid}$···Y(1)–F(5): 103.61(7)°; Cp*$_{centroid}$···Y(1)–F(6): 103.85(7)°; F(1)–Y(1)–F(2): 74.58(10)°; F(1)–Y(1)–F(3): 76.63(10)°; F(1)–Y(1)–F(4): 77.16(11)°; F(1)–Y(1)–F(5): 78.63(11)°; F(1)–Y(1)–F(6): 73.10(9)°; F(2)–Y(1)–F(3): 71.29(10)°; F(3)–Y(1)–F(4): 69.95(11)°; F(4)–Y(1)–F(5): 68.24(10)°; F(5)–Y(1)–F(6): 69.37(10)°; F(6)–Y(1)–F(2): 69.01(9)°.

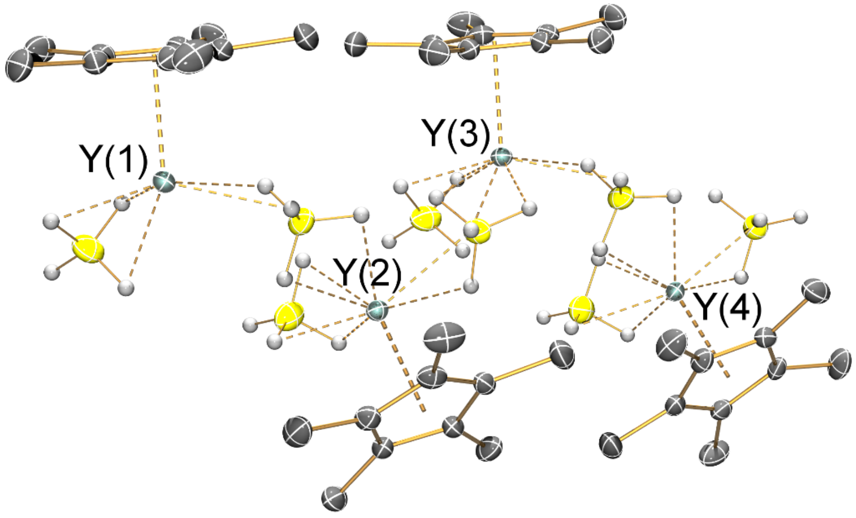

Figure S21. SCXRD structure of **2-Y** with selected atom labelling (Y: cyan, C: grey, B: yellow). Displacement ellipsoids set at 30% probability levels; hydrogen atoms, apart from those on the boron atoms, have been omitted for clarity. Selected bond lengths and angles: Y(1)···Cp*$_{centroid}$: 2.2829(13) Å; Y(1)–B(1): 2.460(4) Å; Y(1)–B(2): 2.654(4) Å; Y(1)–B(8): 2.738(4) Å; Y(1)···Y(2): 5.1097(5) Å; Y(1)···Y(4): 5.1605(5) Å; Cp*$_{centroid}$···Y(1)–B(1): 121.84(9)°; Cp*$_{centroid}$···Y(1)–B(2): 111.47(9)°; Cp*$_{centroid}$···Y(1)–B(8): 112.83(9); Y(1)–B(2)–Y(2): 144.0(2)°; Y(1)–B(8)–Y(4): 146.2(2)°.

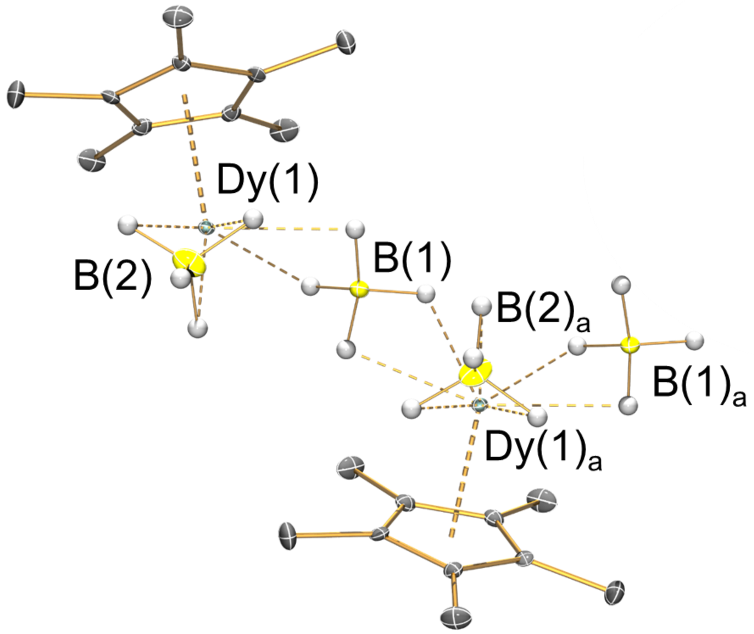

Figure S22. SCXRD structure of **2-Dy** with selected atom labelling (Dy: cyan, C: grey, B: yellow). Displacement ellipsoids set at 30% probability levels; the lattice solvents and the hydrogen atoms, apart from those on the boron atoms, have been omitted for clarity. Selected bond lengths and angles: Dy(1)···$Cp^*_{centroid}$: 2.294(2) Å, Dy(1)–B(1): 2.641(8) Å; Dy(1)–B(2): 2.466(7) Å; Dy(1)···Dy(2): 5.0411(5) Å; $Cp^*_{centroid}$···Dy(1)–B(1): $Cp^*_{centroid}$···Dy(1)–B(2): 123.6(2)°; Dy(1)–B(1)–$Dy(1)_a$: 139.0(3)°; B(1)–$Dy(1)_a$–$B(1)_a$: 107.27(14)°.

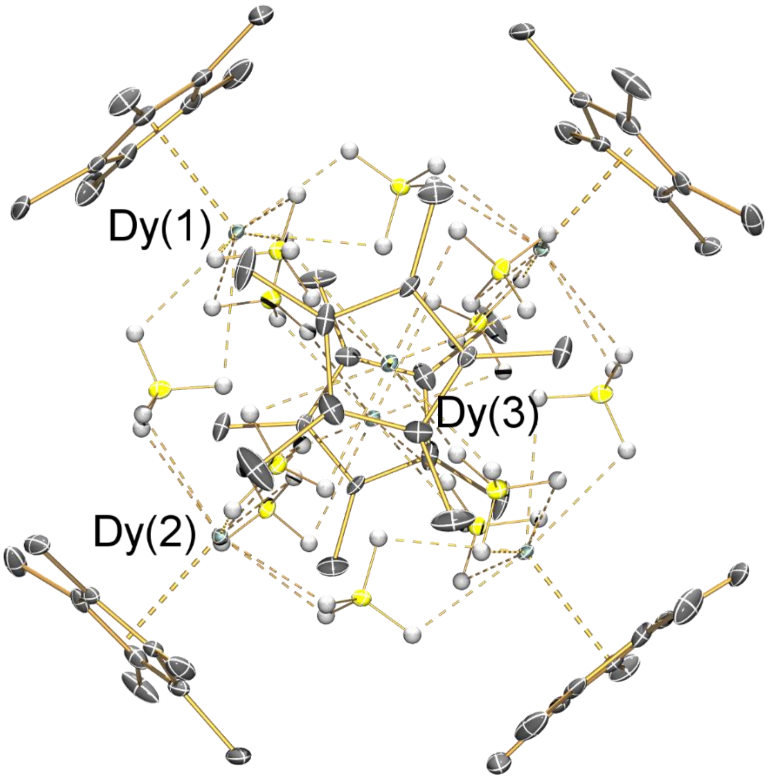

Figure S23. SCXRD structure of **2-Dy·0.33$C_6H_5CH_3$** with selected atom labelling (Dy: cyan, C: grey, B: yellow). Displacement ellipsoids set at 30% probability levels; the lattice solvents and the hydrogen atoms, apart from those on the boron atoms, have been omitted for clarity. Selected ranges of bond lengths and angles: Dy(1)···Cp*$_{centroid}$: 2.329(4) Å; Dy(1)–B(1): 2.817(10) Å; Dy(1)–B(2); 2.681(15) Å; Dy(1)–B(3): 2.681(12) Å; Dy(1)–B(6): 2.907(12) Å; Dy(1)···Dy(2): 5.1171(6) Å; Dy(1)···Dy(2)$_a$: 5.1487(7) Å; Dy(1)···Dy(3): 5.1131(6) Å; Dy(1)···Dy(3)$_a$: 5.4172(8) Å; Cp*$_{centroid}$···Dy(1)–B(1): 111.0(2)°; Cp*$_{centroid}$···Dy(1)–B(2): 112.9(2)°, Cp*$_{centroid}$···Dy(1)–B(3): 112.3(3)°; Cp*$_{centroid}$···Dy(1)–B(6): 117.2(2)°; Cp*$_{centroid}$···Dy(3)–B(5)$_a$: 110.3(3)°; Dy(1)–B(1)–Dy(2): 139.8(4)°; Dy(1)–B(2)–Dy(3): 135.5(4)°; Dy(1)–B(3)–Dy(3): 136.3(5)°; Dy(1)–B(6)–Dy(2): 132.0(4)°.

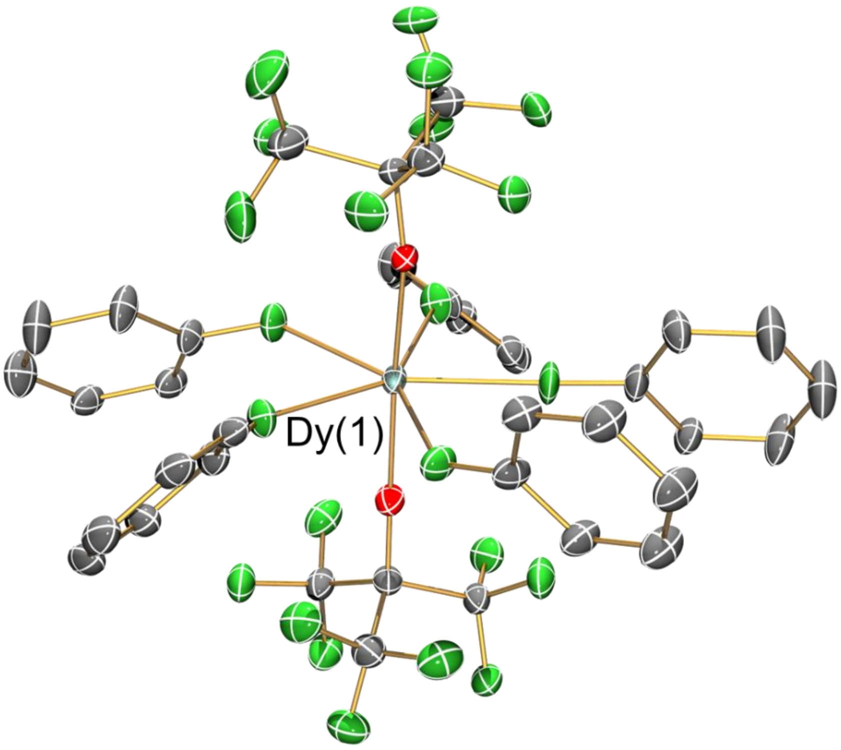

Figure S24. SCXRD structure of **3-Dy** with selected atom labelling (Dy: cyan, C: grey, F: fluorine, O: red). Displacement ellipsoids set at 30% probability levels; hydrogen atoms and the counteranion have been omitted for clarity. Selected ranges of bond lengths and angles: Dy(1)–O(1): 2.081(6) Å; Dy(1)–O(2): 2.088(4) Å; Dy(1)–F(19): 2.359(2) Å; Dy(1)–F(20): 2.370(4) Å; Dy(1)–F(21): 2.351(5) Å; Dy(1)–F(22): 2.367(4) Å; Dy(1)–F(23): 2.337(5) Å; O(1)–Dy(1)–O(2): 176.5(2)°; F(19)–Dy(1)–F(20): 72.0(10)°; F(20)–Dy(1)–F(21): 71.8(2)°; F(21)–Dy(1)–F(22): 72.1(2)°; F(22)–Dy(1)–F(23): 74.7(2)°; F(23)–Dy(1)–F(19): 69.4(10)°; O(2)–Dy(1)–F(19): 88.4(11)°; O(2)–Dy(1)–F(20): 88.9(2)°; O(2)–Dy(1)–F(21): 90.7(2)°; O(2)–Dy(1)–F(22): 91.0(2)°; O(2)–Dy(1)–F(23): 88.3(2)°; O(1)–Dy(1)–F(19): 93.5(11)°; O(1)–Dy(1)–F(20): 94.5(2)°; O(1)–Dy(1)–F(21): 89.5(2)°; O(1)–Dy(1)–F(22): 85.8(2)°; O(1)–Dy(1)–F(23): 89.5(2)°;

Table S3. Crystallographic data for **1-Ln**.

	**1-Y**	**1-Dy**
Formula	$C_{206}H_{123}Al_8F_{233}O_{24}Y_2$	$C_{206}H_{123}Al_8Dy_2F_{233}O_{24}$
molecular mass, g mol^{-1}	7802.70	7949.88
cryst size, mm	0.35 × 0.297 × 0.248	0.164 × 0.144 × 0.098
cryst syst	Monoclinic	Monoclinic
space group	$P2_1/n$	$P2_1/n$
collection temperature, K	100.00(10)	99.9(2)
a, Å	18.06498(10)	18.2139(3)
b, Å	19.02813(13)	19.3140(4)
c, Å	39.8336(2)	40.1803(6)
α, °	90	90
β, °	94.9425(5)	94.8490(10)
γ, °	90	90
V, Å^3	13641.59(14)	14084.2(4)
Z	2	2
ρ_{calcd}, g cm^{-3}	1.900	1.875
μ, mm^{-1}	2.878	5.042
no. of reflections made	149565	148391
no. of unique reflns, R_{int}	29278, 0.0372	29078, 0.1105
no. of reflns with $F^2 > 2\sigma(F^2)$	25953	21765
transmn coeff range	0.286-1.000	0.715-1.000
R, R_w[a] ($F^2 > 2\sigma(F^2)$)	0.1186, 0.3773	0.0910, 0.2580
R, R_w[a] (all data)	0.1243, 0.3889	0.1074, 0.2804
S[a]	2.149	1.195
parameters, restraints	3277, 15976	3277, 16088
max., min. diff map, e Å^{-3}	2.634, –1.514	1.305, –1.211

[a] Conventional $R = \Sigma||F_o| - |F_c||/\Sigma|F_o|$; $R_w = [\Sigma w(F_o^2 - F_c^2)^2/\Sigma w(F_o^2)^2]^{1/2}$; $S = [\Sigma w(F_o^2 - F_c^2)^2$/no. data – no. params)]$^{1/2}$ for all data.

Table S4. Crystallographic data for **2-Ln**.

	**2-Y**	**2-Dy**
Formula	$C_{40}H_{92}B_8Y_4$	$C_{10}H_{23}B_2Dy$
molecular mass, g mol^{-1}	1015.25	327.40
cryst size, mm	0.067 × 0.055 × 0.048	0.254 × 0.228 × 0.206
cryst syst	Monoclinic	Monoclinic
space group	$P2_1/c$	$P2_1/c$
collection temperature, K	99.97(12)	100.00(10)
a, Å	16.9654(2)	8.3735(3)
b, Å	21.7125(2)	20.8167(5)
c, Å	14.55590(10)	8.5037(3)
α, °	90	90
β, °	90.7350(10)	117.930(5)
γ, °	90	90
V, Å^3	5361.39(9)	1309.61(9)
Z	4	4
ρ_{calcd}, g cm^{-3}	1.258	1.661
μ, mm^{-1}	5.759	30.145
no. of reflections made	33940	15015
no. of unique reflns, R_{int}	10851, 0.0358	2738, 0.0685
no. of reflns with $F^2 > 2\sigma(F^2)$	8342	2516
transmn coeff range	0.933-1.000	0.477-1.000
R, R_w[a] ($F^2 > 2\sigma(F^2)$)	0.0332, 0.0852	0.0485, 0.1319
R, R_w[a] (all data)	0.0475, 0.0913	0.0518, 0.1347
S[a]	0.983	1.018
parameters, restraints	585, 1624	148, 272
max., min. diff map, e Å^{-3}	0.466, –0.776	2.019, –1.846

[a] Conventional $R = \Sigma||F_o| - |F_c||/\Sigma|F_o|$; $R_w = [\Sigma w(F_o^2 - F_c^2)^2/\Sigma w(F_o^2)^2]^{1/2}$; $S = [\Sigma w(F_o^2 - F_c^2)^2/\text{no. data} - \text{no. params})]^{1/2}$ for all data.

Table S5. Crystallographic data for **2-Dy·0.33$C_6H_5CH_3$** and **3-Dy**.

	**2-Dy·0.33$C_6H_5CH_3$**	**3-Dy**
Formula	$C_{74}H_{154}B_{12}Dy_6$	$C_{62}H_{25}Al_2DyF_{78}O_8$
molecular mass, g mol^{-1}	2148.68	2596.28
cryst size, mm	0.115 × 0.106 × 0.054	0.161 x 0.084 x 0.08
cryst syst	Triclinic	monoclinic
space group	$P\bar{1}$	$P2_1/c$
collection temperature, K	99.99(10)	103(5)
a, Å	15.6166(3)	21.7517(7)
b, Å	16.3417(5)	20.9203(7)
c, Å	21.7358(5)	18.7796(5)
α, °	90.725(2)	90
β, °	110.278(2)	93.564(3)
γ, °	117.066(3)	90
V, Å^3	4536.0(2)	8529.2(5)
Z	2	4
ρ_{calcd}, g cm^{-3}	1.573	2.022
μ, mm^{-1}	26.167	6.988
no. of reflections made	18473	42310
no. of unique reflns, R_{int}	18473	16771, 0.0918
no. of reflns with $F^2 > 2\sigma(F^2)$	15021	8763
transmn coeff range	0.891-1.000	0.006-0.099
R, R_w[a] ($F^2 > 2\sigma(F^2)$)	0.0532, 0.1447	0.0807, 0.2111
R, R_w[a] (all data)	0.0660, 0.1525	0.1512, 0.2544
S[a]	0.893	0.956
parameters, restraints	1205, 5863	1883, 5946
max., min. diff map, e Å^{-3}	2.915, –2.081	1.049, –1.904

[a] Conventional $R = \Sigma||F_o| - |F_c||/\Sigma|F_o|$; $R_w = [\Sigma w(F_o^2 - F_c^2)^2/\Sigma w(F_o^2)^2]^{1/2}$; $S = [\Sigma w(F_o^2 - F_c^2)^2$/no. data – no. params)]$^{1/2}$ for all data.

Table S6. Crystallographic data for **4-Y**.

	**4-Y**
Formula	$C_{60}H_{54}Al_2F_{57}O_6Si_3Y$
molecular mass, g mol^{-1}	2181.17
cryst size, mm	0.096 × 0.069 × 0.053
cryst syst	triclinic
space group	$P\bar{1}$
collection temperature, K	100.0(3)
a, Å	16.6426(5)
b, Å	16.7663(4)
c, Å	18.4889(3)
α, °	105.461(2)
β, °	104.692(2)
γ, °	113.530(3)
V, Å^3	4163.3(2)
Z	2
ρ_{calcd}, g cm^{-3}	1.740
μ, mm^{-1}	3.232
no. of reflections made	46838
no. of unique reflns, R_{int}	15169, 0.0691
no. of reflns with $F^2 > 2\sigma(F^2)$	11860
transmn coeff range	0.916-1.000
R, R_w[a] ($F^2 > 2\sigma(F^2)$)	0.0906, 0.2566
R, R_w[a] (all data)	0.1057, 0.2741
S[a]	1.021
parameters, restraints	1889, 7027
max., min. diff map, e Å^{-3}	1.513, –1.467

[a] Conventional $R = \Sigma||F_o| - |F_c||/\Sigma|F_o|$; $R_w = [\Sigma w(F_o^2 - F_c^2)^2/\Sigma w(F_o^2)^2]^{1/2}$; $S = [\Sigma w(F_o^2 - F_c^2)^2/\text{no. data} - \text{no. params})]^{1/2}$ for all data.

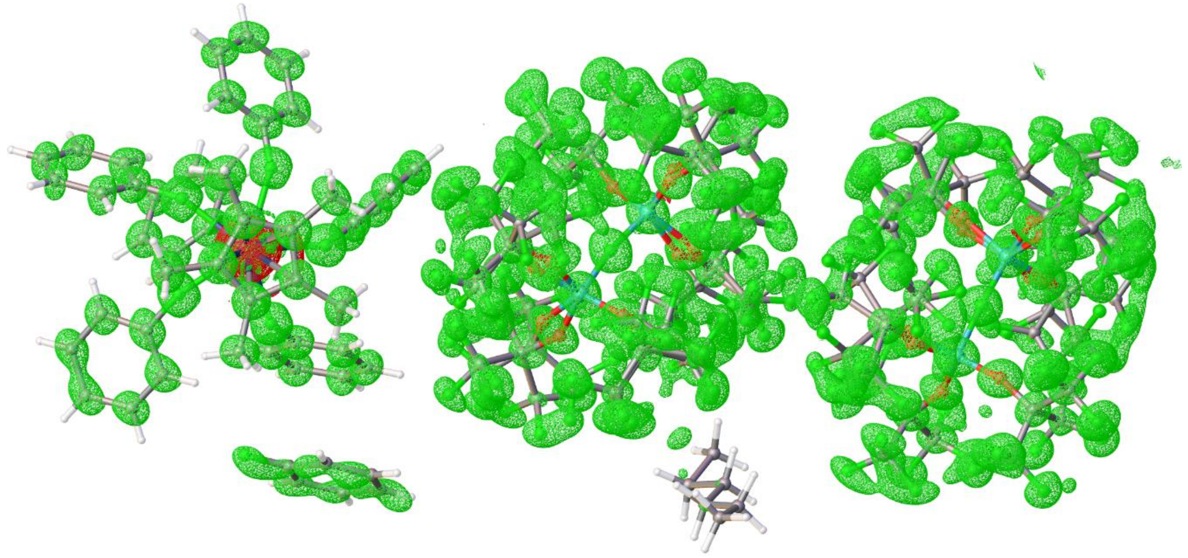

Figure S25. Electron density plot of $2F_o–F_c$ for **1-Y** @ 2 $e^-/Å^3$.

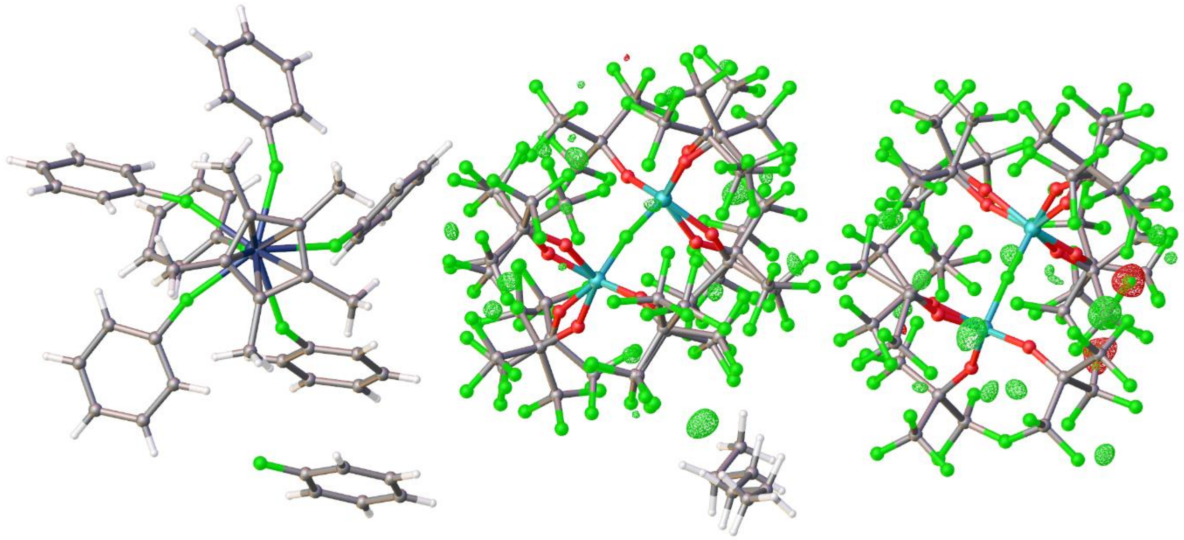

Figure S26. Electron density plot of $F_o–F_c$ for **1-Y** @ 1 $e^-/Å^3$

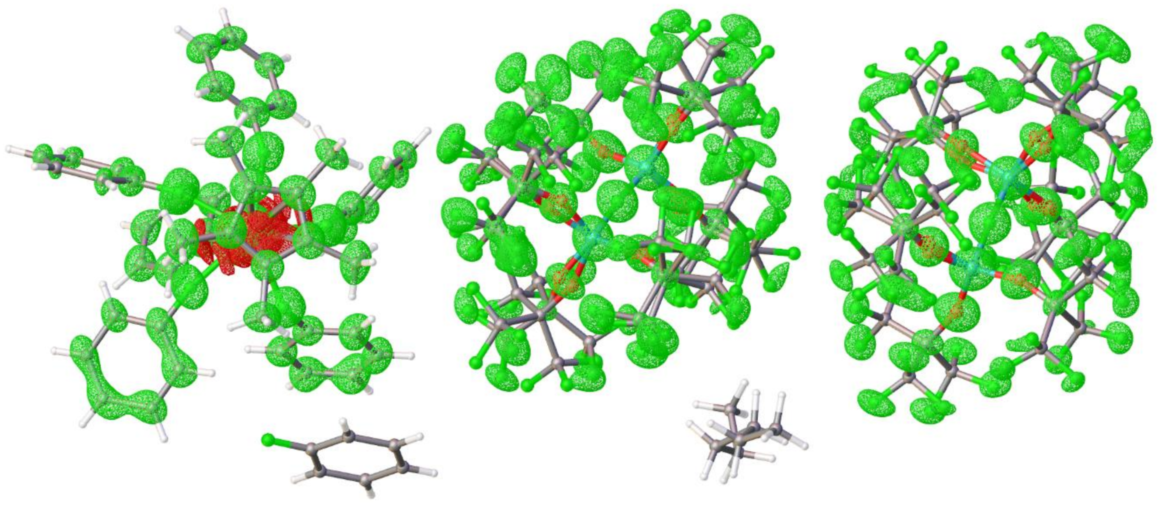

Figure S27. Electron density plot of $2F_o–F_c$ for **1-Dy** @ 2 $e^{–}/Å^3$.

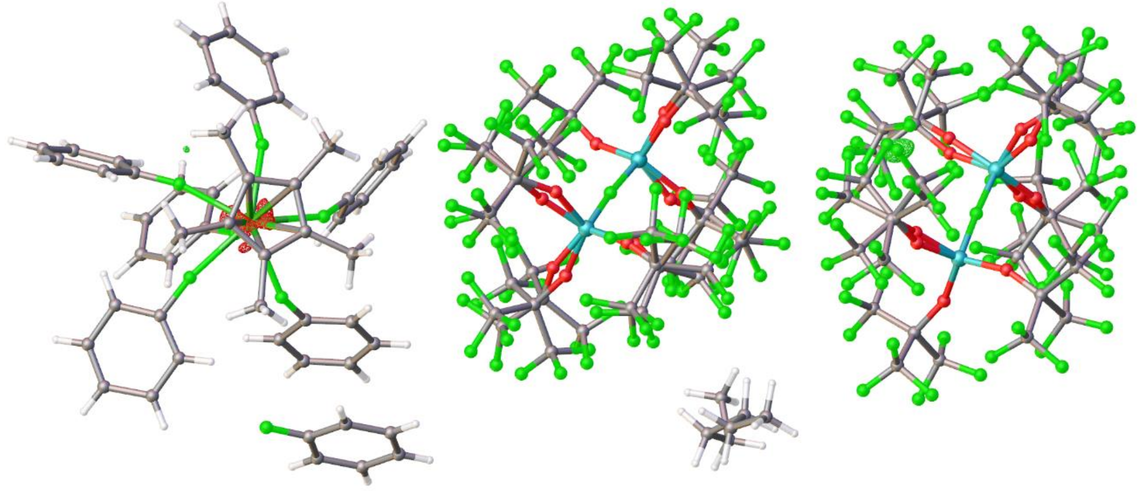

Figure S28. Electron density plot of $F_o–F_c$ for **1-Dy** @ 1 $e^{–}/Å^3$

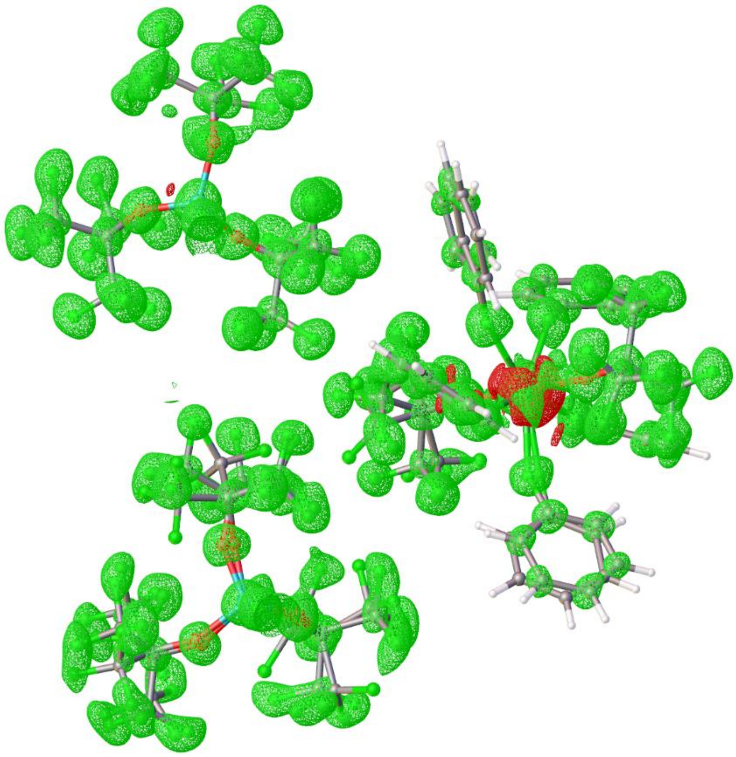

Figure S29. Electron density plot of $2F_o$–F_c for **3-Dy** @ 2.2 e$^–$/Å^3.

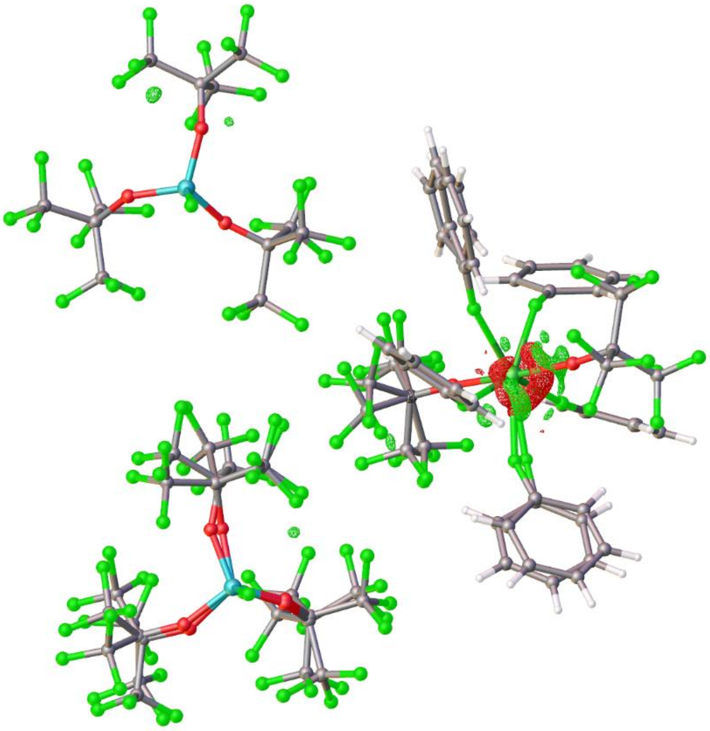

Figure S30. Electron density plot of F_o-F_c for **3-Dy** @ 1 e$^–$/Å^3

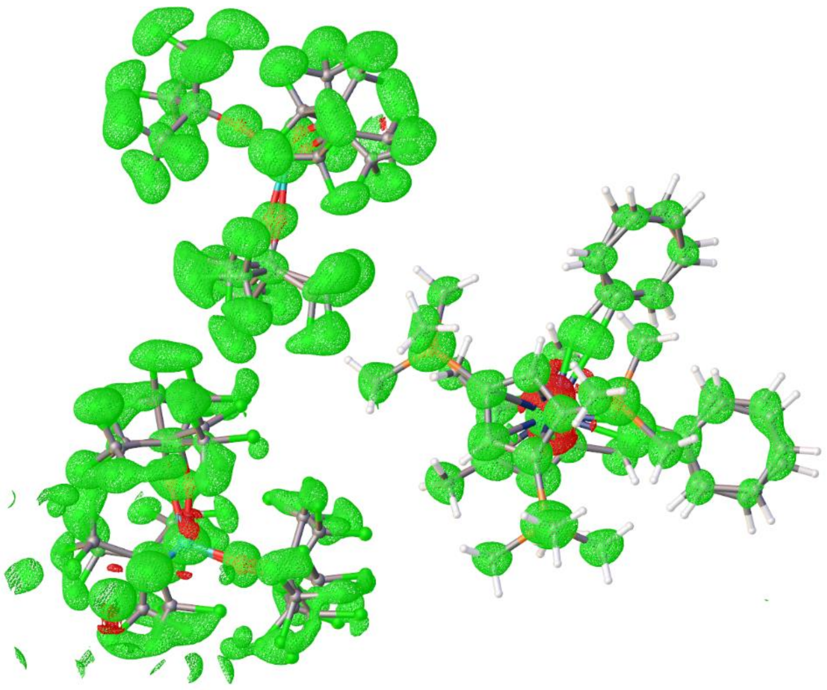

Figure S31. Electron density plot of $2F_o$–F_c for **4-Y** @ 2 e$^-$/Å^3.

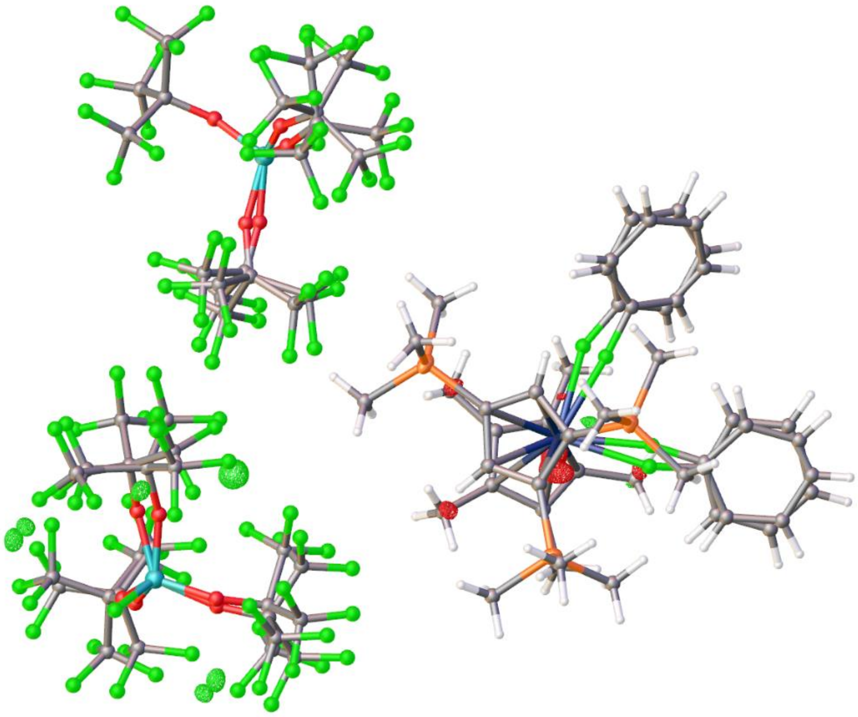

Figure S32. Electron density plot of F_o–F_c for **4-Y** @ 1 e$^-$/Å^3.

6. Magnetic studies

Magnetic measurements were performed using a Quantum Design MPMS3 superconducting quantum interference device (SQUID) magnetometer. A crystalline sample of **1-Dy** (31.8 mg) was crushed with a mortar and pestle under an inert atmosphere, and then loaded into a borosilicate glass NMR tube along with eicosane (17.5 mg), which was then evacuated and flame-sealed to give an ampoule with a length of *ca.* 3 cm. The eicosane was melted by heating the tube gently with a low-power heat gun to immobilize the crystallites. The ampoule was mounted in the centre of a drinking straw using friction by wrapping it with Kapton tape, and the straw was then fixed to the end of the sample rod. The measurements were corrected for the diamagnetism of the straw, borosilicate tube and eicosane using calibrated blanks, for the shape of the sample using Quantum Design Geometry Simulator (correction factor 1.032 for 5 mm measurements and 1.040 for 1 mm measurements), and for the intrinsic diamagnetism of the sample, which was estimated as the molecular weight (g mol^{-1}) multiplied by -0.5×10^{-6} cm^3 K mol^{-1}.

The equilibrium magnetic susceptibility was measured under a 0.1 T field on cooling in temperature settle mode, at 5 K min^{-1} from 300–100 K and 1 K min^{-1} from 100–1.8 K. Equilibrium susceptibility measurements were performed using VSM mode with 5 mm vibrational amplitude and 2 s averaging time (Fig. S33).

Hysteresis measurements were performed between ± 4 T at 2 K, and ± 3 T at temperatures of 4–26 K using VSM mode with a 1 mm vibration amplitude and 2 s averaging time. All field-swept measurements were performed in continuous sweep mode with a sweep rate of 22 Oe s^{-1} across the entire field range. The field was corrected using a Pd reference sample measured under the same conditions and interpolated using B-spline fitting functions.[17]

Alternating frequency (AC) susceptibility measurements were recorded at temperatures between 18–52.5 K, using 8 frequencies per decade between 0.1–1000 Hz with a 5 Oe oscillating field. Due to instrumental limitations, 750 and 1000 Hz frequencies were measured with a 2 Oe oscillating field. Averages were performed for 2 s or for 10 cycles, whichever was longer. The high frequency end of the data was trimmed at low temperatures ($T \leq 36$ K). AC data were fit to the Generalised Debye model (Equation S1) in CC-FIT2 to extract relaxation rates and distributions.[18,19]

$$\chi(\omega) = \chi_S + (\chi_T - \chi_S)\frac{1}{1 + \left(i\omega\tau_{Debye}\right)^{1-\alpha}}$$

Equation S1

where $\tau_{\mathrm{Debye}}^{-1}$ is the relaxation rate, ω is the angular frequency of the AC field and χ_T and χ_S are the isothermal and adiabatic susceptibilities, respectively. There is good agreement between the model and the data (Figs. S34 and S35). The resultant parameters are shown in Table S7.

Magnetisation decay measurements were performed at temperatures between 2–12 K by magnetising the sample at 3 T for a minimum of 15 mins, then measuring continuously (VSM mode, 1 mm vibrational amplitude, 0.5 s averaging time) while sweeping the field to calibrated zero field (set +22.5 Oe). For the 14 and 16 K sweeps, the moment of the first measured point at target field is less than 10% of the saturation value, and therefore these data were discarded. The T ≤ 10 K data was fitted in CC-FIT2 to a double exponential model:[18,19]

$$M(t) = M_{\mathrm{eq}} + (M_0 - M_{\mathrm{eq}})\left(a \cdot \exp\left[-\left(\frac{t}{\tau_1^*}\right)^{\beta_1}\right] + (1-a)\cdot \exp\left[-\left(\frac{t}{\tau_2^*}\right)^{\beta_2}\right]\right)$$

Equation S2

where τ_1^* and τ_2^* are the "characteristic relaxation times" for the two relaxation pathways, M_0 and $M_{\mathrm{eq}} \equiv 0$ are the initial and equilibrium magnetisation, respectively, the stretch parameters $0 < \beta_1$,

$\beta_2 \leq 1$ describe the multi-exponential character, and a is the fraction. The data was cut at 1% of M_0.[19] There is excellent agreement between the model and the data (Figs. S36 and S37). The 12 K data was unable to be fit to Equation S2 and was best fit with a single stretched exponential model:[18,19]

$$M(t) = M_{\mathrm{eq}} + \left(M_0 - M_{\mathrm{eq}}\right) \exp\left[-\left(\frac{t}{\tau^*}\right)^{\beta}\right]$$

Equation S3

with $M_{\mathrm{eq}} \equiv 0$. There is good agreement between the model and the data at short times, though there is a deviation at longer times due to either an imperfect calibration, or impurities/interactions. The resultant parameters for all fits are shown in Table S8.

Waveform measurements[20] were performed on **1-Dy** to obtain low frequency in-phase and out-of-phase susceptibilities at temperatures between 2 and 12 K. The magnet was reset before the sample was cooled in zero field and the moment measured as a function of time as a square wave dc field was applied. The magnitude of the field was ± 8 Oe, the sweep rate 700 Oe s^{-1}, with frequencies in the range 50 μHz ≤ ν ≤ 64 MHz. Measurements were performed using VSM mode with a vibrational amplitude of 1 mm and averaging time of 0.5 s. A fixed voltage range of 1 was used to avoid unevenly spaced datapoints due to autoranging delays. In- and out-of-phase susceptibilities were extracted from the Fourier transform of the magnetic field and sample moment using CC-FIT2,[18,19] using a field window of 1 Oe to discard datapoints measured before and after the square wave field was applied. Attempts to fit the resulting AC data at low temperatures to a Double Generalised Debye model

$$\chi(\omega) = \chi_{total} + \frac{\Delta\chi_1}{1 + \left(i\omega\tau_{1Debye}\right)^{1-\alpha_1}} + \frac{\Delta\chi_2}{1 + \left(i\omega\tau_{2Debye}\right)^{1-\alpha_2}}$$

Equation S4

in CCFIT2 was unsuccessful due to the low point density at low frequencies negatively biasing the fit. We therefore performed the fits by eye and found good agreement between the model and the experimental out-phase-susceptibility χ'' data in the temperature range $2 \leq T \leq 8$ K (Fig. S38). The ratio of the two processes were determined from the ratio $\Delta\chi_1/\Delta\chi_2$. At higher temperatures, we were not able to unequivocally distinguish two relaxation pathways. We fit the 10 and 12 K data to a single Generalised Debye model in Equation S1 (Fig. S38). The resultant parameters for all fits are shown in Table S9.

The temperature dependent relaxation profile was fitted to the model:

$$\log\left[\tau^{-1}\right] = \log\left[10^{-A} e^{\frac{U_{\mathrm{eff}}}{T}} + 10^{R} T^{n} + 10^{-Q_{\mathrm{slow,fast}}}\right]$$

Equation S5

The QTM contribution to the fast rate ($\tau_{\mathrm{QTM}} = 10^{-Q_{\mathrm{fast}}}$) was not included in the fit initially. Raman, Orbach and slow QTM ($\tau_{\mathrm{QTM}} = 10^{-Q_{\mathrm{slow}}}$) parameters were determined by fitting the AC susceptibility, slow and unique waveform rates. The Raman and Orbach parameters were then kept fixed while determining the fast QTM parameter by fitting the fast waveform rates along with the AC susceptibility data. There is excellent agreement between the model and the data (Figure 4).

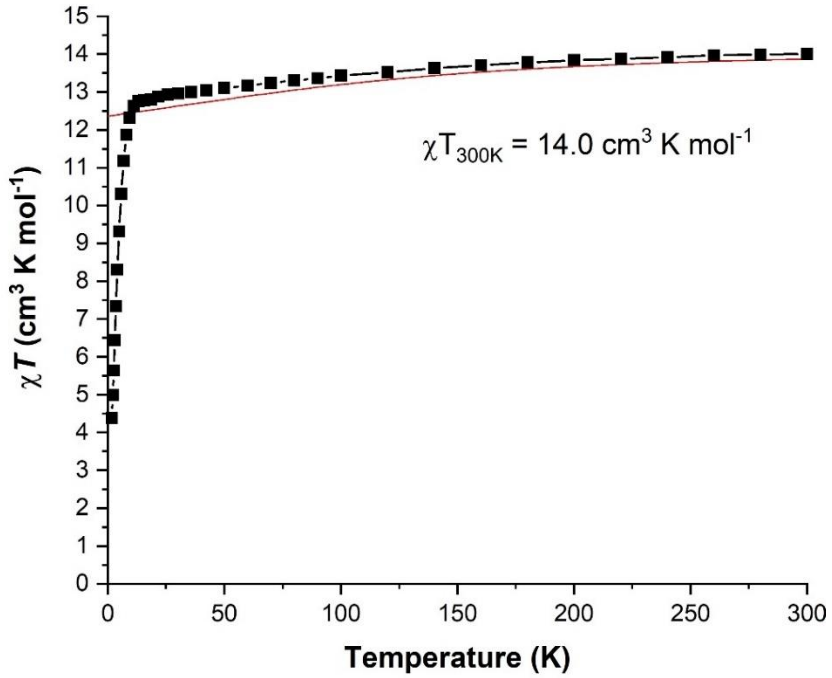

Figure S33. Temperature dependence of the molar magnetic susceptibility (χT) product for powdered **1-Dy** measured under a 0.1 T applied magnetic field (black squares) and predicted values from CASSCF (red line).

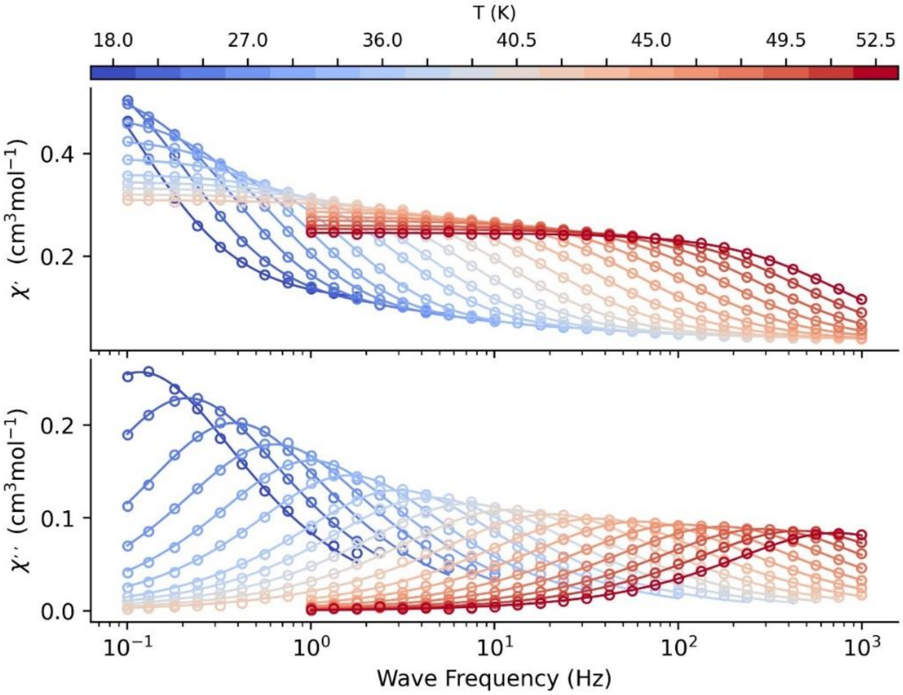

Figure S34. In-phase (top) and out-of-phase (bottom) AC susceptibilities of **1-Dy** in a zero DC field between 18 and 52.5 K. Solid lines are fits to the generalized Debye model in CC-FIT2,[18,19] giving $0.088 \leq \alpha \leq 0.182$.

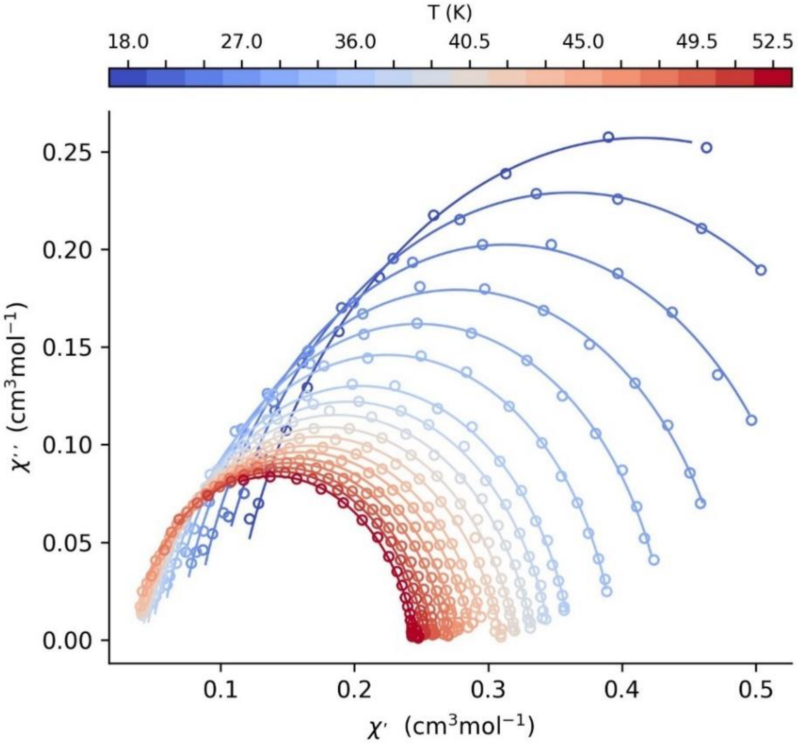

Figure S35. Cole-Cole plot showing fitting of AC data for **1-Dy** in a zero DC field between 18 and 52.5 K. Solid lines are fits to the generalized Debye model in CC-FIT2,[18,19] giving 0.088 ≤ α ≤ 0.182.

Table S7. Best fit parameters to the generalized Debye model for **1-Dy** in zero dc field.

T	τ_{debye}	$\tau_{\mathrm{Debye}}^{\mathrm{err}}$	χ_S	χ_S^{err}	χ_T	χ_T^{err}	α	α^{err}
(K)	(s)		(emu/mol)		(emu/mol)			
18.00	1.36	4.60E-02	1.08E-01	3.03E-03	7.22E-01	1.38E-02	1.13E-01	1.29E-02
21.00	7.49E-01	7.11E-03	9.15E-02	1.44E-03	6.32E-01	3.48E-03	1.06E-01	5.17E-03
24.00	4.14E-01	3.39E-03	7.99E-02	1.34E-03	5.46E-01	2.37E-03	8.91E-02	5.06E-03
27.00	2.50E-01	1.83E-03	6.89E-02	1.17E-03	4.84E-01	1.64E-03	9.28E-02	4.59E-03
30.00	1.59E-01	8.92E-04	6.18E-02	9.71E-04	4.33E-01	9.72E-04	8.80E-02	3.60E-03
33.00	9.90E-02	6.73E-04	5.52E-02	9.09E-04	3.94E-01	9.65E-04	9.52E-02	4.08E-03
36.00	5.35E-02	3.68E-04	4.78E-02	7.51E-04	3.62E-01	7.74E-04	1.19E-01	3.81E-03
37.50	3.56E-02	2.69E-04	4.38E-02	7.25E-04	3.47E-01	7.52E-04	1.37E-01	3.98E-03
39.00	2.22E-02	1.31E-04	4.04E-02	5.32E-04	3.34E-01	5.12E-04	1.53E-01	2.97E-03
40.50	1.34E-02	6.99E-05	3.82E-02	4.70E-04	3.22E-01	3.94E-04	1.66E-01	2.55E-03
42.00	7.85E-03	5.25E-05	3.61E-02	6.11E-04	3.10E-01	4.42E-04	1.74E-01	3.21E-03
43.50	4.48E-03	2.37E-05	3.42E-02	4.77E-04	2.99E-01	4.50E-04	1.79E-01	2.67E-03
45.00	2.61E-03	1.46E-05	3.24E-02	5.61E-04	2.89E-01	3.98E-04	1.82E-01	2.79E-03
46.50	1.53E-03	1.17E-05	3.19E-02	8.33E-04	2.79E-01	4.39E-04	1.78E-01	3.70E-03
48.00	9.15E-04	8.58E-06	3.12E-02	1.11E-03	2.70E-01	4.25E-04	1.75E-01	4.30E-03
49.50	5.53E-04	5.26E-06	3.05E-02	1.18E-03	2.61E-01	3.18E-04	1.67E-01	3.98E-03
51.00	3.49E-04	4.22E-06	3.20E-02	1.54E-03	2.54E-01	3.05E-04	1.59E-01	4.48E-03
52.50	2.22E-04	5.21E-06	3.23E-02	2.95E-03	2.46E-01	3.44E-04	1.53E-01	6.98E-03

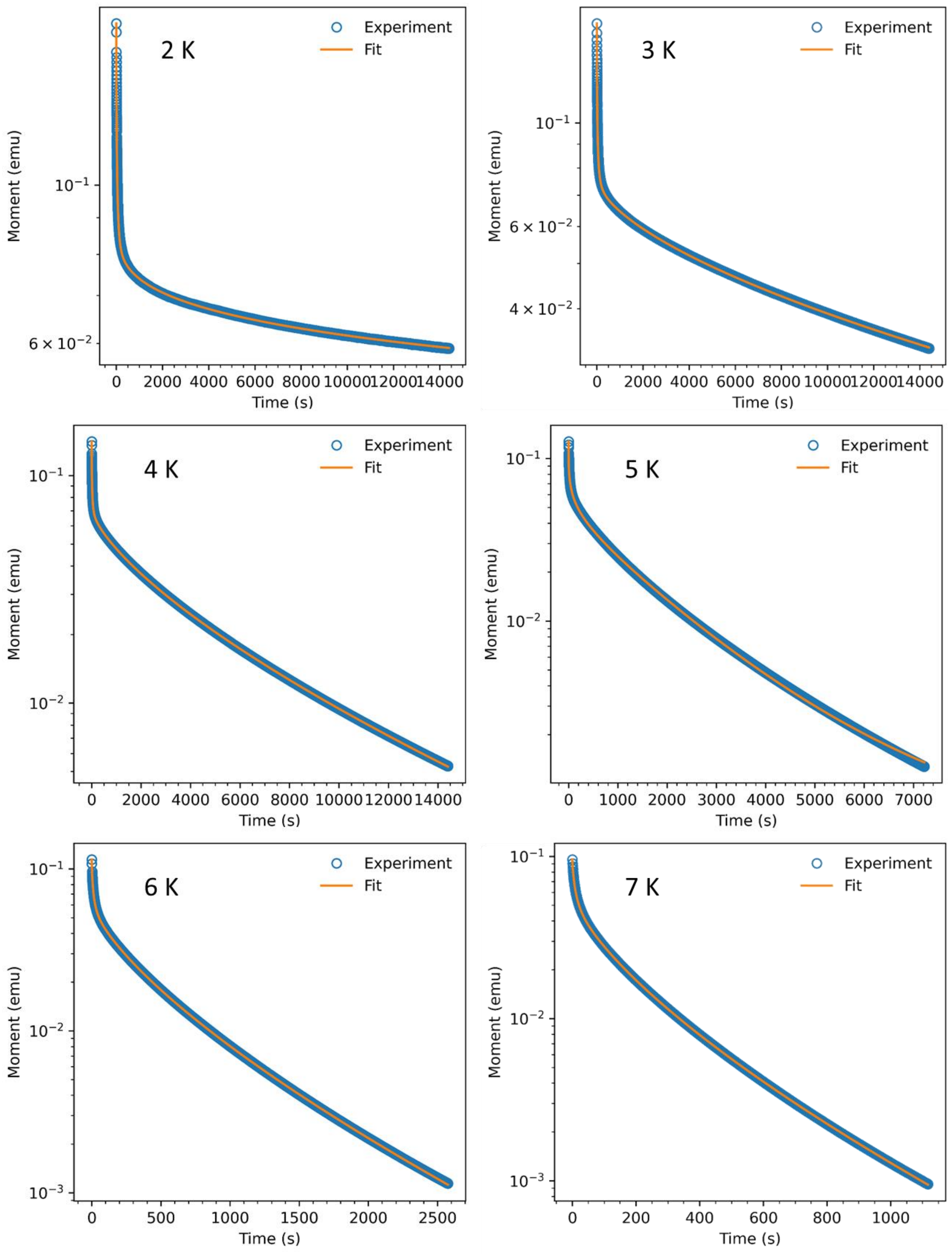

Figure S36. DC decay measurements of **1-Dy** at different indicated temperatures fitted to Equation S2 with the superconducting magnet calibrated such that the real decay field and $M_{eq} \equiv 0$.

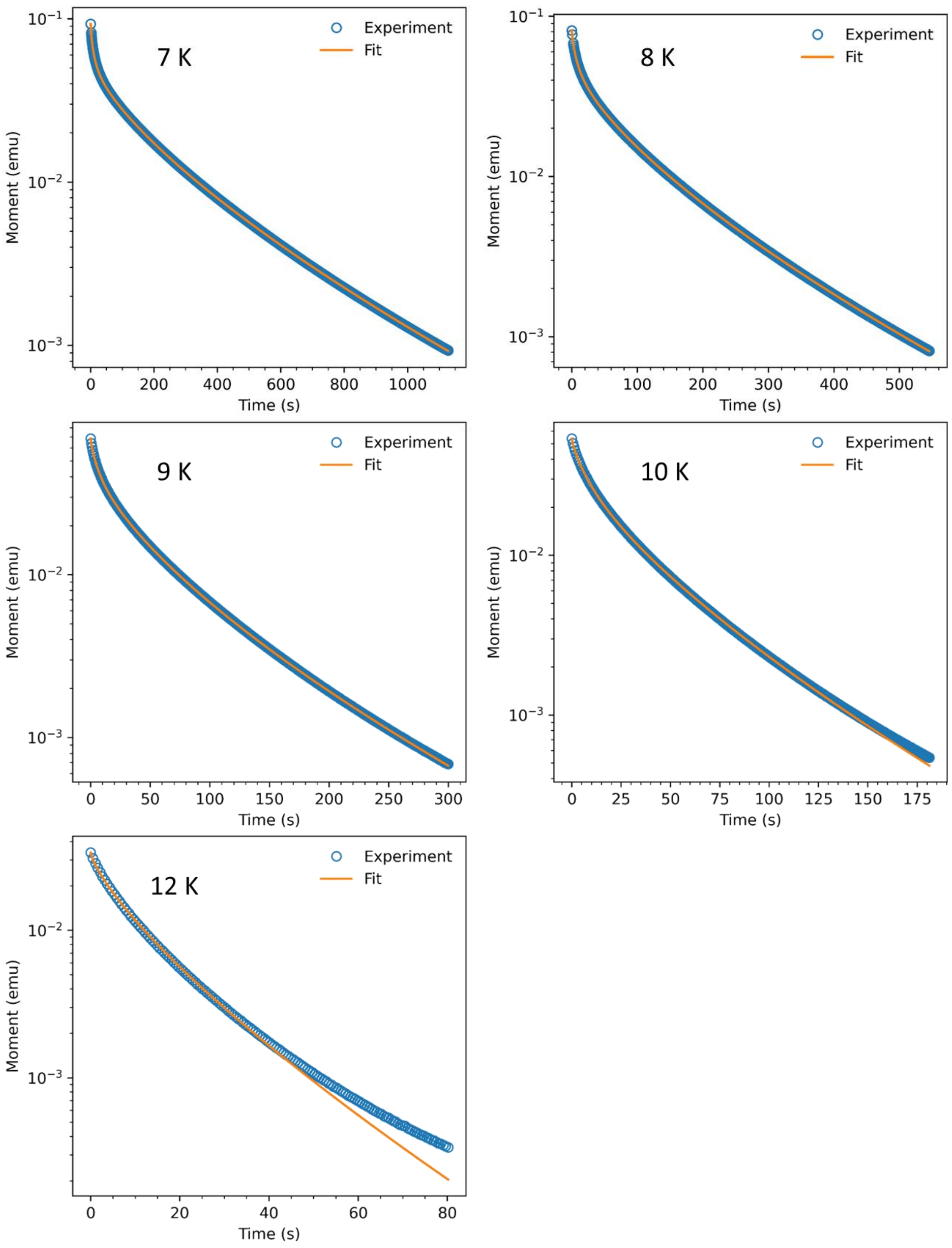

Figure S37. DC decay measurements of **1-Dy** at different indicated temperatures fitted to Equations S2 and S3 with the superconducting magnet calibrated such that the real decay field and $M_{eq} \equiv 0$.

Table S8. Parameters extracted from fitting the dc decay data to Equation S2 ($T \leq 10$ K) and Equation S3 ($T = 12$ K). Values in parentheses represent errors on the fitted parameters. Parameters with no parentheses were fixed.

T (K)	τ_1^* (s)	β_1	τ_2^* (s)	β_2	a	M_{eq} (emu)	M_0 (emu)
2.00	22.2(1)	0.63(1)	3.23(1)E06	0.25(1)	0.44(1)	0	0.17
3.00	14.9(1)	0.53(1)	1.94(1)E04	0.61(1)	0.53(1)	0	0.16
4.00	13.4(1)	0.63(1)	3.63(1)E03	0.70(1)	0.49(1)	0	0.14
5.00	10.3(1)	0.68(1)	1.01(1)E03	0.71(1)	0.47(1)	0	0.12
6.00	8.00(1)	0.68(1)	364(1)	0.71(1)	0.45(1)	0	0.11
7.00	7.53(2)	0.75(1)	149(1)	0.70(1)	0.36(1)	0	0.093
7.00	7.06(1)	0.75(1)	147(1)	0.70(1)	0.37(1)	0	0.096
8.00	5.81(2)	0.79(1)	66.8(2)	0.69(1)	0.30(1)	0	0.082
9.00	5.07(3)	0.96(1)	32.4(3)	0.67(2)	0.17(1)	0	0.069
10.00	6.01(6)	0.92(4)	22.2(7)	0.72(8)	0.18(2)	0	0.054
12.00	-	-	9.31(3)	0.76(2)	0	0	0.034

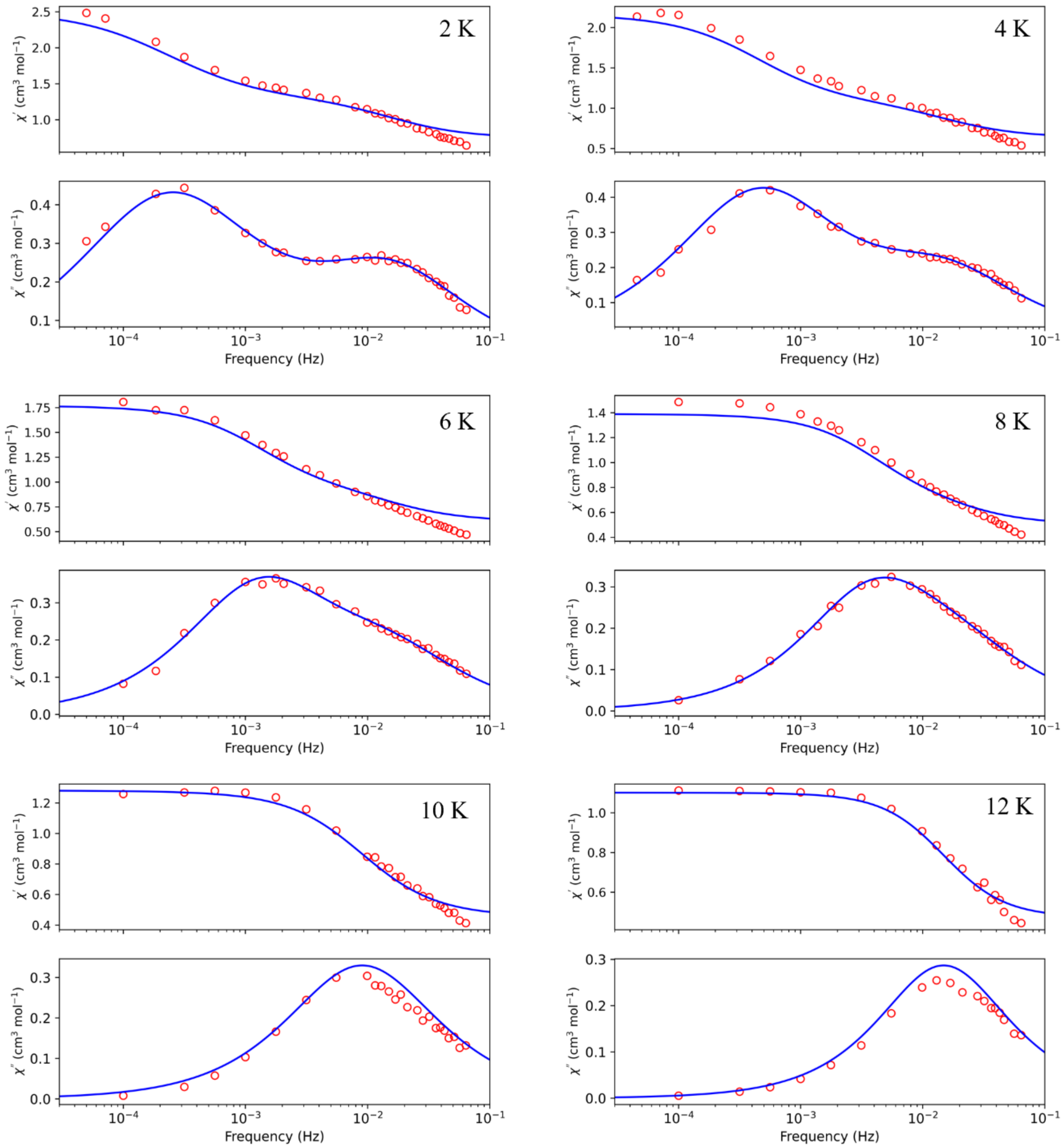

Figure S38. In-phase and out-of-phase susceptibilities extracted from waveform measurements of **1-Dy** at different indicated temperatures. Data at 8 K and below are modelled with Equation S4 and the 10 and 12 K data sets modelled with Equation S1.

Table S9. Parameters extracted from modelling the waveform susceptibility data to Equation S4 ($T \leq 8$ K) and Equation S1 ($T = 10$, 12 K). Values in parentheses represent errors on the fitted parameters. Parameters with no parentheses were fixed.

T (K)	τ_1^* (s)	α_1	τ_2^* (s)	α_2	χ_{total} (cm^3 mol^{-1})	$\Delta\chi_1$ (cm^3 mol^{-1})	$\Delta\chi_2$ (cm^3 mol^{-1})
2.00	10	0.15	670	0.25	0.74	0.53	1.25
4.00	10	0.15	350	0.20	0.63	0.42	1.12
6.00	10	0.15	120	0.15	0.60	0.33	0.84
8.00	8	0.15	40	0.12	0.5	0.24	0.65
10.00	-	-	17.6(9)	0.15(3)	0.45(2)	0	0.83(4)
12.00	-	-	10.7(5)	0.06(4)	0.47(2)	0	0.63(3)

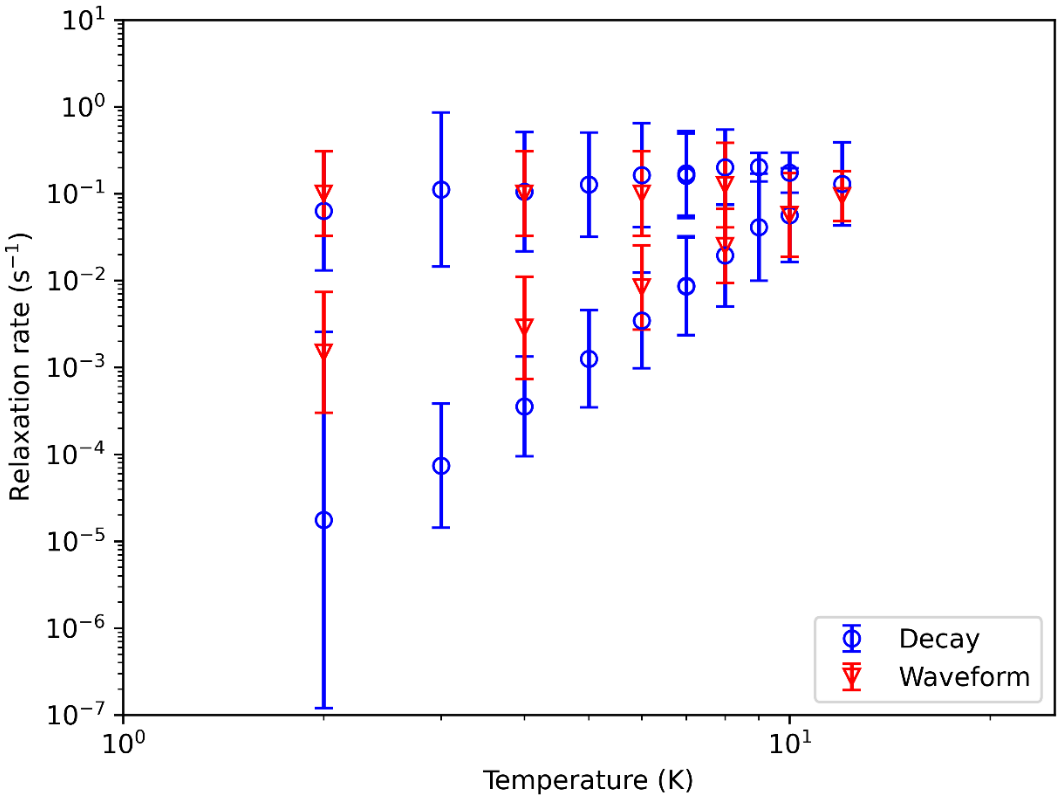

Figure S39. Comparison of relaxation rates extracted from DC decay (blue) and waveform (red) measurements of **1-Dy**.

7. CASSCF-SO calculations

OpenMolcas[21] was used to perform CASSCF-SO calculations on $[Dy(Cp^*)(FPh)_6]^{2+}$, $[Dy(Cp^*)]^{2+}$, $[Dy(Cp^*)(FPh)]^{2+}$, $[Dy\{OC(CF_3)_3\}_2(FPh)_5]^{+}$ and the Dy analogue of $[Y(Cp'')(Cp^*)(FPh)_2]^{+}$ to determine the electronic structure. The molecular geometry from the single crystal XRD structures of **1-Dy**, **3-Dy** and **4-Y** were used with no optimization. Electron integrals were performed in the SEWARD module using basis sets from ANO-RCC library[22–25] with VTZP quality for the Dy atom, VDZP quality for the cyclopentadienyl C atoms and the fluorobenzene F atoms, and VDZ quality for all remaining atoms, employing the second-order DKH Hamiltonian for scalar relativistic effects. Resolution of identity Cholesky decomposition (RICD) of the two-electron integrals with atomic compact Cholesky decomposition (acCD) auxiliary basis sets was employed to reduce computational demand.[26]

The molecular orbitals (MOs) were optimised in state-averaged CASSCF (SA-CASSCF) calculations in the RASSCF module, where the active space was defined by the nine electrons in the seven 4f orbitals of Dy(III). A SA-CASSCF calculation was performed for the lowest 18 sextets, where these states were then mixed by spin orbit coupling in the RASSI module. SINGLE_ANISO was used to decompose the resulting spin-orbit wave functions into the CF Hamiltonian formalism.[27] Diamond was employed for molecular graphics.[28]

Table S10. Electronic structure of $[Dy(Cp^*)(FPh)_6]^{2+}$, calculated with the crystal field parameters obtained from CASSCF-SO using the solid-state geometry of **1-Dy** in zero-field. Each row corresponds to a Kramers doublet.

Energy (cm^{-1})	Energy (K)	g_x	g_y	g_z	Angle[a] (deg)	Wavefunction	$\langle J_z \rangle$
0.00	0.00	0.0002	0.0003	19.89	--	99.9% $\lvert\pm 15/2\rangle$	±7.497
271.54	390.74	0.02	0.02	17.1	1.7	99% $\lvert\pm 13/2\rangle$	±6.480
400.94	576.95	0.4	0.5	14.3	2.1	98% $\lvert\pm 11/2\rangle$	±5.438
472.47	679.89	0.2	0.6	11.7	4.6	97% $\lvert\pm 9/2\rangle$	±4.411
531.72	765.15	0.1	0.5	9.0	6.3	95% $\lvert\pm 7/2\rangle$ + 3% $\lvert\pm 3/2\rangle$	±3.399
588.96	847.51	3.2	3.8	6.1	11.1	89% $\lvert\pm 5/2\rangle$ + 6% $\lvert\pm 1/2\rangle$ + 2% $\lvert\pm 9/2\rangle$ + 2% $\lvert\mp 3/2\rangle$	±2.304
639.37	920.05	2.7	4.7	10.5	88.1	75% $\lvert\pm 3/2\rangle$ + 13% $\lvert\mp 1/2\rangle$ + 6% $\lvert\mp 5/2\rangle$ + 3% $\lvert\pm 7/2\rangle$	±1.021
686.73	988.20	0.5	2.0	17.7	89.5	76% $\lvert\pm 1/2\rangle$ + 17% $\lvert\mp 3/2\rangle$ + 3% $\lvert\pm 5/2\rangle$ + 2% $\lvert\mp 1/2\rangle$	±0.204

[a] The angle between the g_z value of the excited Kramers doublet and the ground Kramers doublet.

Table S11. Electronic structure of $[Dy(Cp^*)]^{2+}$, calculated with the crystal field parameters obtained from CASSCF-SO using the solid-state geometry of **1-Dy** in zero-field. Each row corresponds to a Kramers doublet.

Energy (cm^{-1})	Energy (K)	g_x	g_y	g_z	Angle[a] (deg)	Wavefunction	$\langle J_z \rangle$
0.00	0.00	0.0000	0.0000	20.0	--	100% $\lvert\pm 15/2\rangle$	±7.500
331.37	476.84	0.0001	0.0001	17.2	0.4	99.97% $\lvert\pm 13/2\rangle$	±6.499
537.27	773.12	0.003	0.003	14.5	0.4	99.9% $\lvert\pm 11/2\rangle$	±5.499
691.68	995.33	0.0004	0.006	11.8	0.4	99.9% $\lvert\pm 9/2\rangle$	±4.499
833.25	1199.04	0.005	0.01	9.1	0.9	99.8% $\lvert\pm 7/2\rangle$	±3.498
965.12	1388.80	0.1	0.1	6.4	1.1	99.6% $\lvert\pm 5/2\rangle$	±2.495
1071.10	1541.30	3.2	3.4	3.7	7.1	97% $\lvert\pm 3/2\rangle$ + 3% $\lvert\mp 1/2\rangle$	±1.448
1135.39	1633.82	1.2	7.2	13.8	89.9	97% $\lvert\pm 1/2\rangle$ + 3% $\lvert\mp 3/2\rangle$	±0.453

[a] The angle between the g_z value of the excited Kramers doublet and the ground Kramers doublet.

Table S12. Electronic structure of $[Dy(Cp^*)(FPh)]^{2+}$, calculated with the crystal field parameters obtained from CASSCF-SO using the solid-state geometry of **1-Dy** in zero-field. Each row corresponds to a Kramers doublet.

Energy (cm^{-1})	Energy (K)	g_x	g_y	g_z	Angle[a] (deg)	Wavefunction	$\langle J_z \rangle$
0.00	0.00	0.0000	0.0000	20.0	--	100% $\lvert \pm 15/2 \rangle$	±7.500
467.22	672.33	0.0000	0.0000	17.1	0.4	100% $\lvert \pm 13/2 \rangle$	±6.500
796.71	1146.46	0.002	0.002	14.4	0.4	99.9% $\lvert \pm 11/2 \rangle$	±5.499
1038.27	1494.05	0.001	0.004	11.7	0.5	99.9% $\lvert \pm 9/2 \rangle$	±4.499
1231.94	1772.74	0.01	0.01	9.1	1.5	99.9% $\lvert \pm 7/2 \rangle$	±3.498
1389.75	1999.84	0.06	0.09	6.4	2.2	99.6% $\lvert \pm 5/2 \rangle$	±2.494
1504.66	2165.20	3.4	3.6	3.7	31.2	97% $\lvert \pm 3/2 \rangle$ + 3% $\lvert \mp 1/2 \rangle$	±1.441
1571.03	2260.70	1.1	6.9	14.0	89.8	97% $\lvert \pm 1/2 \rangle$ + 3% $\lvert \mp 3/2 \rangle$	±0.446

[a] The angle between the g_z value of the excited Kramers doublet and the ground Kramers doublet.

Table S13. Electronic structure of $[Dy\{OC(CF_3)_3\}_2(FPh)_5]^+$, calculated with the crystal field parameters obtained from CASSCF-SO using the solid-state geometry of **3-Dy** in zero-field. Each row corresponds to a Kramers doublet.

Energy (cm^{-1})	Energy (K)	g_x	g_y	g_z	Angle[a] (deg)	Wavefunction	$<J_z>$
0.00	0.00	0	0	19.9	--	100% |± 15/2⟩	±7.500
531.60	764.96	0.0004	0.0004	17.0	2.5	99.7% |± 13/2⟩	±6.497
944.40	1358.98	0.002	0.002	14.3	5.2	98% |± 11/2⟩	±5.487
1208.02	1738.33	0.2	0.2	11.8	12.7	92% |± 9/2⟩ + 5% |± 7/2⟩	±4.430
1274.87	1834.53	0.8	2.9	17.2	87.4	74% |± 1/2⟩ + 8% |∓ 1/2⟩ + 7% |∓ 3/2⟩ +7% |± 3/2⟩ + 4% |± 5/2⟩	±0.444
1304.30	1876.88	3.3	4.2	9.0	69.6	49% |± 3/2⟩ + 22% |± 7/2⟩ + 10% |∓ 1/2⟩ + 10% |± 5/2⟩ + 4% |± 1/2⟩ + 2% |± 9/2⟩ + 2% |∓ 3/2⟩	±1.767
1330.96	1915.24	0.3	3.7	9.2	53.9	38% |± 7/2⟩ + 32% |± 3/2⟩ + 18% |± 5/2⟩ + 4% |∓ 5/2⟩ + 2% |± 9/2⟩ + 2% |∓ 1/2⟩ + 2% |∓ 7/2⟩ + 2% |∓ 3/2⟩	±2.207
1356.01	1951.29	0.3	4.2	13.4	60.5	60% |± 5/2⟩ + 30% |± 7/2⟩ + 2% |± 1/2⟩ + 2% |∓ 5/2⟩ + 2% |∓ 7/2⟩	±2.505

[a] The angle between the g_z value of the excited Kramers doublet and the ground Kramers doublet

Table S14. Electronic structure of the Dy analogue of [Y(Cp‴)(Cp*)(FPh)$_2$]$^+$, calculated with the crystal field parameters obtained from CASSCF-SO using the solid-state geometry of **4-Y** in zero-field. Each row corresponds to a Kramers doublet.

Energy (cm^{-1})	Energy (K)	g_x	g_y	g_z	Angle[a] (deg)	Wavefunction	<J_z>
0.00	0.00	0.0000	0.0001	19.9	--	98.0% |± 15/2⟩	±7.461
297.66	428.33	0.005	0.005	17.1	0.8	98.5% |± 13/2⟩	±6.469
524.24	754.37	0.05	0.05	14.5	0.4	97% |± 11/2⟩	±5.519
674.32	970.33	0.6	0.8	11.8	2.3	97% |± 9/2⟩	±4.474
773.54	1113.11	2.8	3.8	8.6	25.5	82% |± 7/2⟩ + 12% |± 3/2⟩ + 2%|∓ 1/2⟩ + 2% |∓ 5/2⟩	±3.036
821.36	1181.92	2.5	6.7	9.5	88.4	46% |± 5/2⟩ + 34% |± 1/2⟩ + 8% |∓ 3/2⟩ + 7% |∓ 7/2⟩ + 2% |± 3/2⟩	±1.052
890.94	1282.04	0.6	1.8	15.3	89.9	38% |± 3/2⟩ + 33% |∓ 5/2⟩ + 9% |∓ 3/2⟩ + 7% |∓ 1/2⟩ + 5% |± 5/2⟩ + 4% |± 7/2⟩ + 2% |∓ 1/2⟩	±0.194
1093.00	1572.81	0.02	0.04	19.7	89.9	38% |± 1/2⟩ + 24% |∓ 3/2⟩ + 15% |∓ 1/2⟩ + 9% |± 3/2⟩ + 8% |± 5/2⟩ + 3% |∓ 5/2⟩ + 2% |∓ 7/2⟩	±0.019

[a] The angle between the g_z value of the excited Kramers doublet and the ground Kramers doublet.

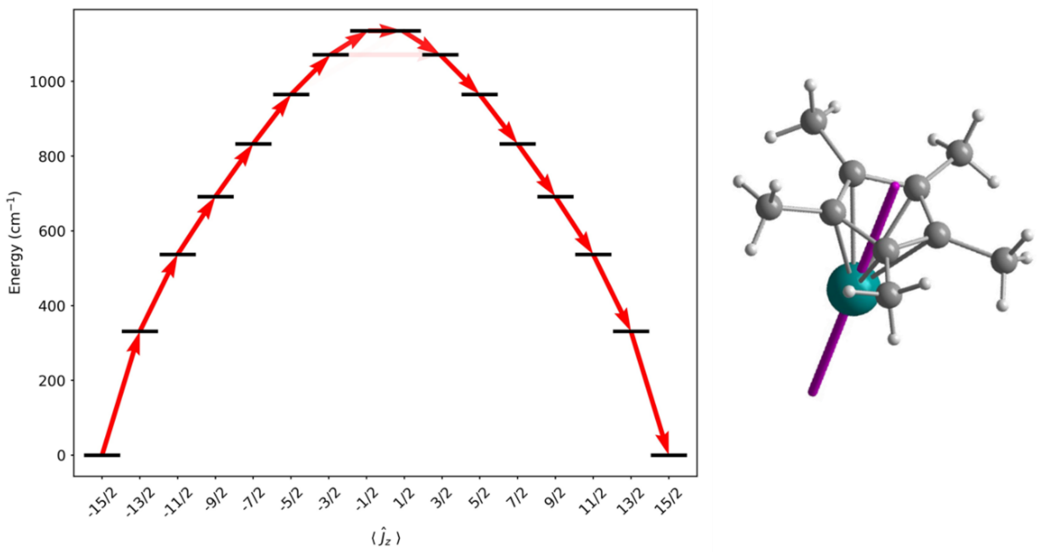

Figure S40. Energy barrier to magnetic relaxation for a model of $[Dy(Cp^*)]^{2+}$. Electronic states from CASSCF-SO calculations, labelled with their dominant m_J composition in the $J = 15/2$ basis. Arrows represent the Orbach relaxation pathway, where the opacity of the arrows is proportional to the transition probability approximated with the average matrix elements of magnetic moment connecting the states, $\gamma_{ij} = (1/3)[|\langle i|\mu_x|j\rangle|^2 + |\langle i|\mu_y|j\rangle|^2 + |\langle i|\mu_z|j\rangle|^2]$, normalized from each departing state and commencing from $|-15/2\rangle$ (left). Denotation of the g_z axis (purple) within the solid-state structure at the ground state (right).

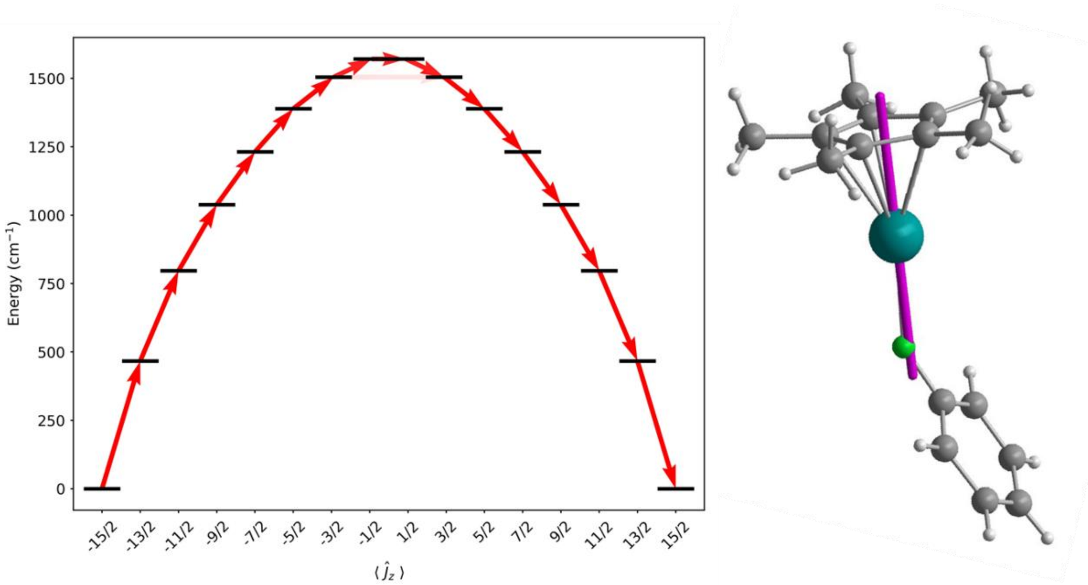

Figure S41. Energy barrier to magnetic relaxation for a model of $[Dy(Cp^*)(PhF)]^{2+}$. Electronic states from CASSCF-SO calculations, labelled with their dominant m_J composition in the J = 15/2 basis. Arrows represent the Orbach relaxation pathway, where the opacity of the arrows is proportional to the transition probability approximated with the average matrix elements of magnetic moment connecting the states, $\gamma_{ij} = (1/3)[|\langle i|\mu_x|j\rangle|^2 + |\langle i|\mu_y|j\rangle|^2 + |\langle i|\mu_z|j\rangle|^2]$, normalized from each departing state and commencing from |–15/2> (left). Denotation of the g_z axis (purple) within the solid-state structure at the ground state (right).

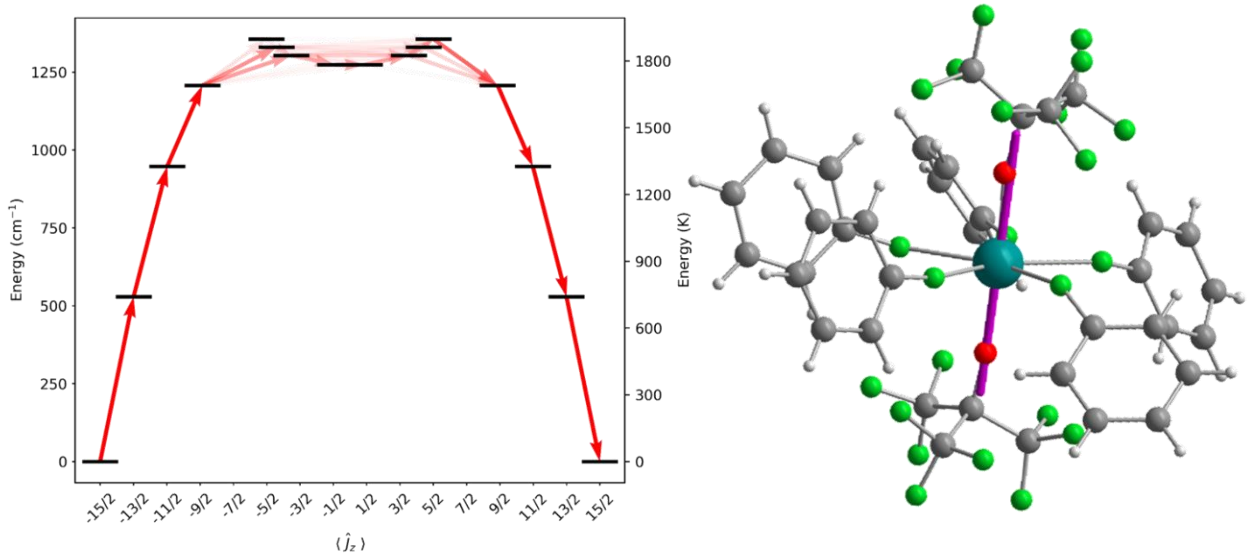

Figure S42. Energy barrier to magnetic relaxation for a model of $[Dy\{OC(CF_3)_3\}_2(FPh)_5]^+$. Electronic states from CASSCF-SO calculations, labelled with their dominant m_J composition in the *J* = 15/2 basis. Arrows represent the Orbach relaxation pathway, where the opacity of the arrows is proportional to the transition probability approximated with the average matrix elements of magnetic moment connecting the states, $\gamma_{ij} = (1/3)[|\langle i|\mu_x|j\rangle|^2 + |\langle i|\mu_y|j\rangle|^2 + |\langle i|\mu_z|j\rangle|^2]$, normalized from each departing state and commencing from |–15/2> (left). Denotation of the g_z axis (purple) within the solid-state structure at the ground state (right).

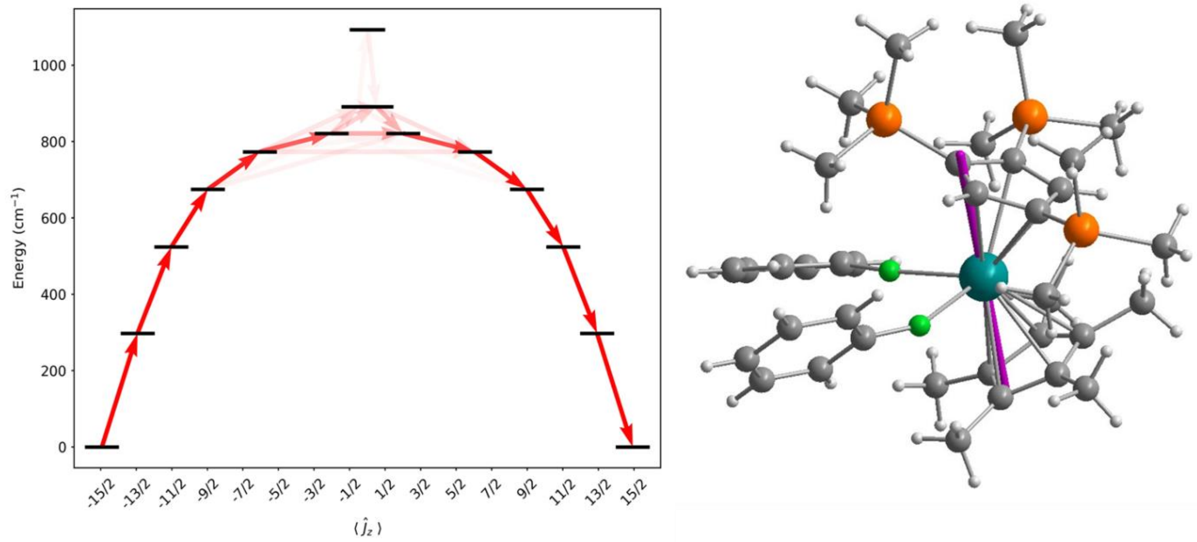

Figure S43. Energy barrier to magnetic relaxation for a model of the Dy analogue of $[Y(Cp''')(Cp^*)(FPh)_2]^+$. Electronic states from CASSCF-SO calculations, labelled with their dominant m_J composition in the J = 15/2 basis. Arrows represent the Orbach relaxation pathway, where the opacity of the arrows is proportional to the transition probability approximated with the average matrix elements of magnetic moment connecting the states, $\gamma_{ij} = (1/3)[|<i|\mu_x|j>|^2 + |<i|\mu_y|j>|^2 + |<i|\mu_z|j>|^2]$, normalized from each departing state and commencing from |–15/2> (left). Denotation of the g_z axis (purple) within the solid-state structure at the ground state (right).

8. Dipolar calculations

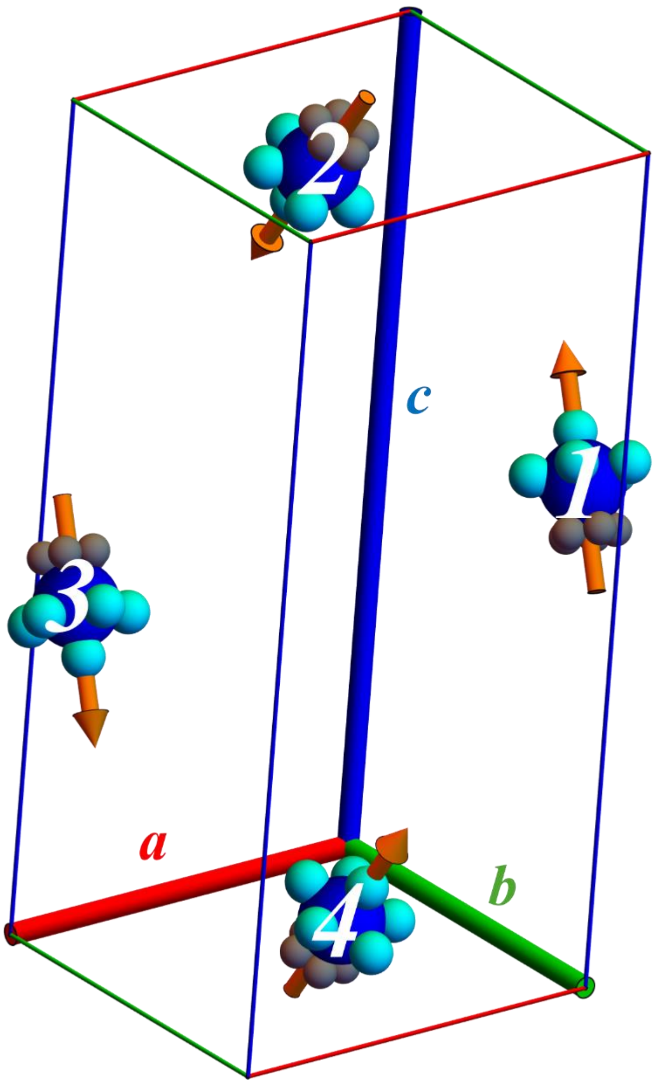

Figure S44. Assignment of molecules in the unit cell of **1-Dy** used in the dipolar calculations.

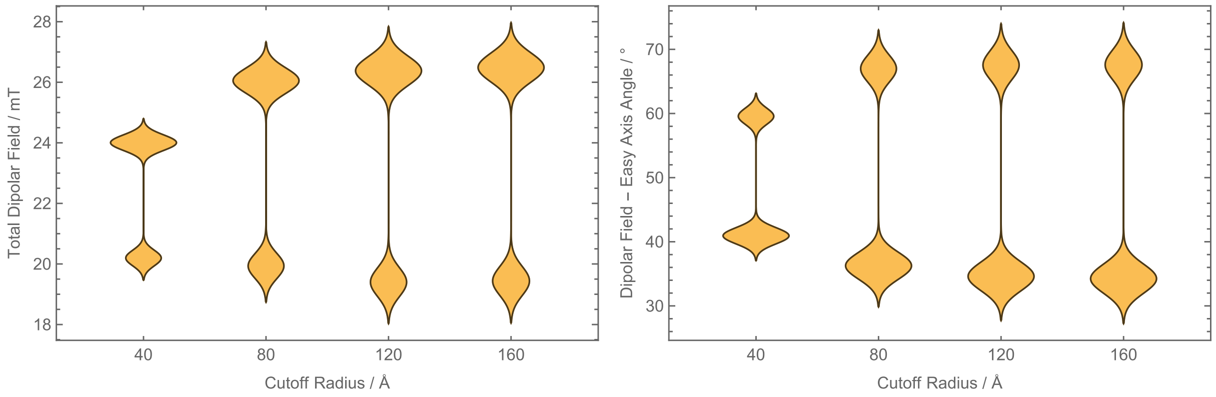

Figure S45. Tests for calculating the internal dipolar fields of **1-Dy** showing that the results are well converged by 160 Å.

9. References

1 F. Neese, *WIREs Comput. Mol. Sci.*, 2012, **2**, 73–78.

2 F. Neese, *WIREs Comput. Mol. Sci.*, 2018, **8**, e1327.

3 S. Lehtola, C. Steigemann, M. J. T. Oliveira and M. A. L. Marques, *SoftwareX*, 2018, **7**, 1–5.

4 E. Caldeweyher, C. Bannwarth and S. Grimme, *J. Chem. Phys.*, 2017, **147**, 034112.

5 E. Caldeweyher, S. Ehlert, A. Hansen, H. Neugebauer, S. Spicher, C. Bannwarth and S. Grimme, *J. Chem. Phys.*, 2019, **150**, 154122.

6 F. Weigend and R. Ahlrichs, *Phys. Chem. Chem. Phys.*, 2005, **7**, 3297–3305.

7 F. Weigend, *Phys. Chem. Chem. Phys.*, 2006, **8**, 1057.

8 D. Andrae, U. Häußermann, M. Dolg, H. Stoll and H. Preuß, *Theor. Chim. Acta*, 1990, **77**, 123–141.

9 E. A. Kabova, C. D. Blundell, C. A. Muryn, G. F. S. Whitehead, I. J. Vitorica-Yrezabal, M. J. Ross and K. Shankland, *CrystEngComm*, 2022, **24**, 4337–4340.

10 *CrysAlis Pro*, Agilent Technologies, Yarnton, England, 2010.

11 V. Petříček, M. Dušek and L. Palatinus, *Zeitschrift für Krist. - Cryst. Mater.*, 2014, **229**, 345–352.

12 C. F. Macrae, I. Sovago, S. J. Cottrell, P. T. A. Galek, P. McCabe, E. Pidcock, M. Platings, G. P. Shields, J. S. Stevens, M. Towler and P. A. Wood, *J. Appl. Crystallogr.*, 2020, **53**, 226–235.

13 G. M. Sheldrick, *Acta Crystallogr. Sect. C Struct. Chem.*, 2015, **71**, 3–8.

14 O. V. Dolomanov, L. J. Bourhis, R. J. Gildea, J. A. K. Howard and H. Puschmann, *J. Appl. Crystallogr.*, 2009, **42**, 339–341.

15 L. J. Farrugia, *J. Appl. Crystallogr.*, 2012, **45**, 849–854.

16 *POV-Ray*, Persistence of Vision Raytracer Pty. Ltd., Williamstown, Australia, 2004.

17 A. Antonov, QuantileRegression package for Mathematica, https://github.com/antononcube/MathematicaForPrediction/blob/master/QuantileRegression.m.

18 D. Reta and N. F. Chilton, *Phys. Chem. Chem. Phys.*, 2019, **21**, 23567–23575.

19 W. J. A. Blackmore, G. K. Gransbury, P. Evans, J. G. C. Kragskow, D. P. Mills and N. F. Chilton, *Phys. Chem. Chem. Phys.*, 2023, **25**, 16735–16744.

20 J. D. Hilgar, A. K. Butts and J. D. Rinehart, *Phys. Chem. Chem. Phys.*, 2019, **21**, 22302–22307.

21 I. Fdez. Galván, M. Vacher, A. Alavi, C. Angeli, F. Aquilante, J. Autschbach, J. J. Bao, S. I. Bokarev, N. A. Bogdanov, R. K. Carlson, L. F. Chibotaru, J. Creutzberg, N. Dattani, M. G. Delcey, S. S. Dong, A. Dreuw, L. Freitag, L. M. Frutos, L. Gagliardi, F. Gendron, A. Giussani, L. González, G. Grell, M. Guo, C. E. Hoyer, M. Johansson, S. Keller, S. Knecht, G. Kovačević, E. Källman, G. Li Manni, M. Lundberg, Y. Ma, S. Mai, J. P. Malhado, P. Å. Malmqvist, P. Marquetand, S. A. Mewes, J. Norell, M. Olivucci, M. Oppel, Q. M. Phung, K. Pierloot, F. Plasser, M. Reiher, A. M. Sand, I. Schapiro, P. Sharma, C. J. Stein, L. K. Sørensen, D. G. Truhlar, M. Ugandi, L. Ungur, A. Valentini, S. Vancoillie, V. Veryazov, O. Weser, T. A. Wesołowski, P. O. Widmark, S. Wouters, A. Zech, J. P. Zobel and R. Lindh, *J. Chem. Theory Comput.*, 2019, **15**, 5925–5964.

22 B. O. Roos, V. Veryazov and P.-O. Widmark, *Theor. Chem. Acc.*, 2004, **111**, 345–351.

23 B. O. Roos, R. Lindh, P. Å. Malmqvist, V. Veryazov and P. O. Widmark, *J. Phys. Chem. A*, 2004, **108**, 2851–2858.

24 B. O. Roos, R. Lindh, P.-Å. Malmqvist, V. Veryazov and P.-O. Widmark, *J. Phys. Chem. A*, 2005, **109**, 6575–6579.

25 B. O. Roos, R. Lindh, P.-Å. Malmqvist, V. Veryazov, P.-O. Widmark and A. C. Borin, *J. Phys. Chem. A*, 2008, **112**, 11431–11435.

26 F. Aquilante, R. Lindh and T. Bondo Pedersen, *J. Chem. Phys.*, 2007, **127**, 114107(1–7).

27 L. F. Chibotaru and L. Ungur, *J. Chem. Phys.*, 2012, **137**, 064112.

28 *Diamond - Crystal and Molecular Structure Visualization*, Crystal Impact - Dr. H. Putz & Dr. K. Brandenburg GbR, Bonn, Germany.
